# Supplementary material for: Functional Analysis of KIT Gene Structural Mutations Causing the Porcine Dominant White Phenotype Using Genome Edited Mouse Models
Source: Front Genet. 2020 Mar 3;11:138. doi: 10.3389/fgene.2020.00138 (PMC7063667; doi:10.3389/fgene.2020.00138)
Supplement: Supplementary file 1 [file DataSheet_1.pdf]

KIT D17/+

```
                                             852                                                                                                                                        991
C01_NJ0512276W_¼¼XM002098-2_2098-kit-FO-tR1    (85) CTTAGACTGTAGTCATTTCCACATAGTATTTCAAACGAA-----------------------------------------------------------------------------------------------------
                                 Kit KO rc   (852) CTTAGACTGTAGTCATTTCCACATAGTATTTCAAACGAAGCGGGGAGAAAGCCTACCGTGACCTTCAGGTCTACATTTGTTATAGTTGAGCAAAATCAAATGGATTGCTAGTTTCAGCCTATCGTGAAAAATTAAGGCTA
                                 Consensus   (852) CTTAGACTGTAGTCATTTCCACATAGTATTTCAAACGAA
                                             992                                                                                                                                        1131
C01_NJ0512276W_¼¼XM002098-2_2098-kit-FO-tR1   (124) --------------------------------------------------------------------------------------------------------------------------------------------
                                 Kit KO rc   (992) TTTTTGTTTATTTCGCACACCCCCAATTATACAATTATAGCTAAAAATTCTATTAACAAGCCTTAGTGGTACTAACATGTGACATTACAAGGTAGGAGTTGTAATGATCATAGAAAACAATTTAGAATCAAGACTGGATA
                                 Consensus   (992)
                                             1132                                                                                                                                       1271
C01_NJ0512276W_¼¼XM002098-2_2098-kit-FO-tR1   (124) --------------------------------------------------------------------------------------------------------------------------------------------
                                 Kit KO rc  (1132) CCAAAGCACCCTGGGTAGACTCATGAGATGGAGAAAGGTACTCACATTTCCTTTGACCACGTAATTCGAATCATTCCTGATGTCTCTGGCTAGCCCGAAATCGCAAATCTTTGTGATCCGCCCGTGAGTGAGGAGGATAT
                                 Consensus  (1132)
                                             1272                                                                                                                                       1411
C01_NJ0512276W_¼¼XM002098-2_2098-kit-FO-tR1   (124) --------------------------------------------------------------------------------------------------------------------------------------------
                                 Kit KO rc  (1272) TCCTGGCTGCCAAATCTCTGTGAATACACTGTTGGGGGAGAAAAGGAAAGACAGCAACAGCCATTCATTTCAGCTCTCCGAGAGAAGATGATTTTAATACTACAATGGTGTTTACATGAAAAAAAAAAAGAAACTTTCGT
                                 Consensus  (1272)
                                             1412                                                                                                                                       1551
C01_NJ0512276W_¼¼XM002098-2_2098-kit-FO-tR1   (124) --------------------------------------------------------TTGTGTTTCACAGGATCGGGGCTCTAATGACTATAATTGCTTTCCAACCCAATTTTAATAAACGTCTTTCTCCCTTCCTTCCAT
                                 Kit KO rc  (1412) TTTAAAGTATTTAGCACACCGAAAATAAAAATTATGGTGCAAGTGATATTCCCGTTTTGTGTTTCACAGGATCGGGGCTCTAATGACTATAATTGCTTTCCAACCCAATTTTAATAAACGTCTTTCTCCCTTCCTTCCAT
                                 Consensus  (1412)                                                         TTGTGTTTCACAGGATCGGGGCTCTAATGACTATAATTGCTTTCCAACCCAATTTTAATAAACGTCTTTCTCCCTTCCTTCCAT
                                             1552            1568
C01_NJ0512276W_¼¼XM002098-2_2098-kit-FO-tR1   (208) TCACAGGAAAATGCATT
                                 Kit KO rc  (1552) TCACAGGAAAATGCATT
                                 Consensus  (1552) TCACAGGAAAATGCATT
```

KIT $^{GtoA/+}$

```
                                                              10        20        30        40        50        60
                                                    tatctcactgttacagttgtgagattttgaagatagACCCCCCCCCCCCCCCATCATCCTAAGGTT
2727.seq(1>3519)                                 →  tatctcactgttacagttgtgagattttgaagatagaccccccccccccccatcatcctaaggtt
KIT110-2651-CKIT-D-5SEQR2-E10.ab(1>187)          ←                                      ACCCCCCCCCCCCCCCATCATCCTAAGGTT

                                                         70        80        90       100       110       120       130
                                                    TCTGGAACCCAGCTGGGGATGATCAGATAGCTATTGGCTTTGTGTCTACCATTGTTCCTCCCTGG
2727.seq(1>3519)                                 →  tctggaacccagctggggatgatcagatagctattggctttgtgtctaccattgttcctccctgg
KIT110-2651-CKIT-D-5SEQR2-E10.ab(1>187)          ←  TCTGGAACCCAGCTGGGGATGATCAGATAGCTATTGGCTTTGTGTCTACCATTGTTCCTCCCTGG

                                                              140       150       160       170       180       190
                                                    GGAGGTGACTGGCCTGGGAGTCGACACTGCTTATCCACTGCTAGCTTTGATACTAAGGCTGCTTC
2727.seq(1>3519)                                 →  ggaggtgactggcctgggagtcgacactgcttatccactgctagctttgatactaaggctgcttc
KIT110-2651-CKIT-D-5SEQR2-E10.ab(1>187)          ←  GGAGGTGACTGGCCTGGGAGTCGACACTGCtTATCCACTGCTAG
KIT110-2651-CKIT-F-5SEQR4-E08.ab(1>460)          ←                                       CTGCTAGCTTTGATACTAAGGCTGCTTC

                                                         200       210       220       230       240       250       260
                                                    TCCTCTCCTTACCACTAAGGGTTTTTTGGTTCTTGTTTTGTTTTTTTTTTTGTTTTTTGTTTTTT
2727.seq(1>3519)                                 →  tcctctccttaccactaagggttttttggttcttgttttgttttttttttttgttttttgtttttt
KIT110-2651-CKIT-F-5SEQR4-E08.ab(1>460)          ←  TCCTCTCCTTACCACTAAGGGTTTTTTGGTTCTTGTTTTGTTTTTTTTTTTGTTTTTTGTTTTTT

                                                              270       280       290       300       310       320
                                                     TTTCCTTGCTATCTTCCTAATAGACTCGTACATAGAAAGAGACGTGACTCCTGCCATCATGGAAG
2727.seq(1>3519)                                 →   tttccttgctatcttcctaatagactcgtacatagaaagagacgtgactcctgccatcatggaag
KIT110-2651-CKIT-F-5SEQR4-E08.ab(1>460)          ←   TTTCCTTGCTATCTTCCTAATAGACTCGTACATAGAAAGAGACGTGACTCCTGCCATCATGGAAG
KIT110-2651-CKIT-D-5SEQF2-E09.ab(1>170)          →                                                ACTCCTGCCATCATGGAAG

                                                         330       340       350       360       370       380       390
                                                    ATGACGAGCTGGCTCTGGACCTGGATGATTTGCTGAGCTTCTCCTACCAGGTGGCCAAGGGCATG
2727.seq(1>3519)                                 →  atgacgagctggctctggacctggatgatttgctgagcttctcctaccaggtggccaagggcatg
KIT110-2651-CKIT-F-5SEQR4-E08.ab(1>460)          ←  ATGACGAGCTGGCTCTGGACCTGGATGATtTGCTGAGCtTCTCCcTA
KIT110-2651-CKIT-D-5SEQF2-E09.ab(1>170)          →  ATGACGAGCTGGCTCTGGACCTGGATGATTTGCTGAGCTTCTCCTACCAGGTGGCCAAGGGCATG

                                                              400       410       420       430       440       450
                                                    GCGTTCCTCGCCTCCAAGAATGTAAGTGGCATGGAGTCTCATGAGAGGAGCACGGCATCCTTTTT
2727.seq(1>3519)                                 →  gcgttcctcgcctccaagaatgtaagtggcatggagtctcatgagaggagcacggcatccttttt
KIT110-2651-CKIT-D-5SEQF2-E09.ab(1>170)          →  GCGTTCCTCGCCTCCAAGAATGTAAGTGGCATGGAGTCTCATGAGAGGAGCACGGCATCCTTTTT

                                                         460       470       480       490       500       510       520
                                                    TTTTTTTTTTTTTTTTTTTTTtaaagccttgaacatttcctagtcctagttagaattcagagtgt
2727.seq(1>3519)                                 →  tttttttttttttttttttttttaaagccttgaacatttcctagtcctagttagaattcagagtgt
KIT110-2651-CKIT-D-5SEQF2-E09.ab(1>170)          →  TTTTTTTTTTTTTTTTTTTTT

                                                              530       540       550       560       570       580
                                                    cattttgaagtgtggtaaccgaaagcaggggaaaatttcgtttcttcacgttccaacttctgtct
2727.seq(1>3519)                                 →  cattttgaagtgtggtaaccgaaagcaggggaaaatttcgtttcttcacgttccaacttctgtct

                                                         590       600       610       620       630       640       650
                                                    ctttggaattcctgttctaatTTCTAAGCTTGAAAGCGCAAACCTGTCTAAATGAGCCTCCATGA
2727.seq(1>3519)                                 →  ctttggaattcctgttctaatttctaagcttgaaagcgcaaacctgtctaaatgagcctccatga
KIT110-2651-CKIT-D-5SEQF1-E07.ab(1>350)          →                       TTCTAaGCTTGAaAGCGCAaACCTGTCTAAATGAGCCTCCATGA

                                                              660       670       680       690       700       710
                                                    ATATTCTTTTATATGCAGTGAGTTCATTTAAAAGCTTTAAGATGTAGGATGCGCTTCTAGAAAAT
2727.seq(1>3519)                                 →  atattcttttatatgcagtgagttcatttaaaagctttaagatgtaggatgcgcttctagaaaat
KIT110-2651-CKIT-D-5SEQF1-E07.ab(1>350)          →  ATATTCTTTTATATGCAGTGAGTTCATTTAAAAGCTTTAAGATGTAGGATGCGCTTCTAGAAAAT
```

720 730 740 750 760 770 780

AGAAGGATAGTGGTTTTATCAGAAAATGGGGGAGCCAGGAGTGTGACTCCCTCACTGTTACTGTG

2727.seq(1>3519) → agaaggatagtggttttatcagaaaatgggggagccaggagtgtgactccctcactgttactgtg
KIT110-2651-CKIT-D-5SEQF1-E07.ab(1>350) → AGAAGGATAGTGGTTTTATCAGAAAATGGGGGAGCCAGGAGTGTGACTCCCTCACTGTTACTGTG

790 800 810 820 830 840

CCTGGTAAGGCATATGACTGTGTATGTGAGACCCACGTACTGTGTATGTGTATGTGACTCACAGA

2727.seq(1>3519) → cctggtaaggcatatgactgtgtatgtgagacccacgtactgtgtatgtgtatgtgactcacaga
KIT110-2651-CKIT-D-5SEQF1-E07.ab(1>350) → CCTGGTAAGGCATATGACTGTGTATGTGAGACCCACGTACTGTGTATGTGTATGTGACTCACAGA

850 860 870 880 890 900 910

GGATCAGGACATGAGAAGGGGTCAGAGAGAGCATGGAGCAGCTAGGGAAAGGATAGAGACGGGAA

2727.seq(1>3519) → ggatcaggacatgagaaggggtcagagagagcatggagcagctagggaaaggatagagacgggaa
KIT110-2651-CKIT-D-5SEQF1-E07.ab(1>350) → GGATCAGGACATGAGAAGGGGTCAGAGAGAGCATGGAGCAGCTAGGGAAAGGATAGAGACGGGAA

920 930 940 950 960 970

GAGAGGGATGGCACGGGATGAAAGTGTTCCGTCTGAGGCGGAAAGAaccaggggaggaaggtgtg

2727.seq(1>3519) → gagagggatggcacgggatgaaagtgttccgtctgaggcggaaagaaccaggggaggaaggtgtg
KIT110-2651-CKIT-D-5SEQF1-E07.ab(1>350) → GAGAGGGATGGCACGGGATGAAAGTGTTCCGTCTGaGGCGGAAAGA

980 990 1000 1010 1020 1030 1040

gccaagagtgaggtcacagtggctcttctagtgacattcccaggtaactaccaagctaaaagaca

2727.seq(1>3519) → gccaagagtgaggtcacagtggctcttctagtgacattcccaggtaactaccaagctaaaagaca

1050 1060 1070 1080 1090 1100

ggctgaaatgaaagaccctcttccttgtgtccttgggagaagacgtcaagttgaagctgtgaaac

2727.seq(1>3519) → ggctgaaatgaaagaccctcttccttgtgtccttgggagaagacgtcaagttgaagctgtgaaac

1110 1120 1130 1140 1150 1160 1170

TTTTTTTTTTTTTTTTTTTTTGGAGAAAACGTTCAAAGAGATGCATACAAAATGAACTTTCATTT

2727.seq(1>3519) → tttttttttttttttttttttggagaaaacgttcaaagagatgcatacaaaatgaactttcattt
KIT110-1-2651-CKIT-D-SEQR1-F02.a(1>663) ←

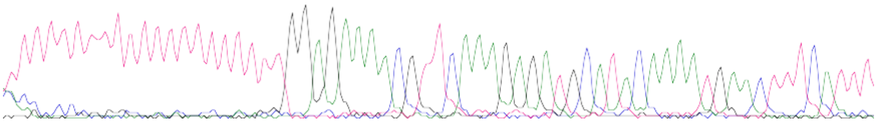

TTTTTTTTTTTTTTTTTTTTTGGAGAAAACGTTCAAAGAGATGCATACAAAATGAACTTTCATTT

1180 1190 1200 1210 1220 1230

TAGAAATGGGATTTGACTATTTATAATGCATTTTCCTGTGAATGGAAGGAAGGGAGAAAGACGTT

2727.seq(1>3519) → tagaaatgggatttgactatttataatgcattttcctgtgaatggaaggaagggagaaagacgtt
KIT110-1-2651-CKIT-D-SEQR1-F02.a(1>663) ←

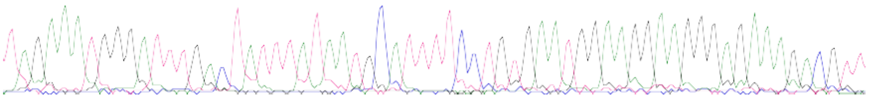

TAGAAATGGGATTTGACTATTTATAATGCATTTTCCTGTGAATGGAAGGAAGGGAGAAAGACGTT

1240 1250 1260 1270 1280 1290 1300

TATTAAAATTGGGTTGGAAAGCAATTATAGTCATTAGAGCCCCGATCCTGTGAAACACAAAACGG

2727.seq(1>3519) → tattaaaattgggttggaaagcaattatagtcattagagccccgatcctgtgaaacacaaaacgg
KIT110-1-2651-CKIT-D-SEQR1-F02.a(1>663) ←

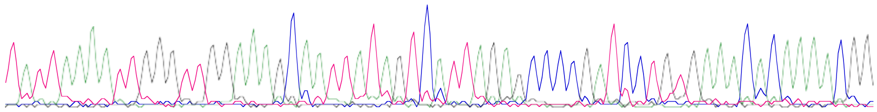

TATTAAAATTGGGTTGGAAAGCAATTATAGTCATTAGAGCCCCGATCCTGTGAAACACAAAACGG

1310 1320 1330 1340 1350 1360

GAATATCACTTGCACCATAATTTTTATTTTCGGTGTGCTAAATACTTTAAAACGAAAGTTTCTTT

2727.seq(1>3519) gaatatcacttgcaccataatttttattttcggtgtgctaaatactttaaaacgaaagtttcttt
KIT110-1-2651-CKIT-D-SEQR1-F02.a(1>663)

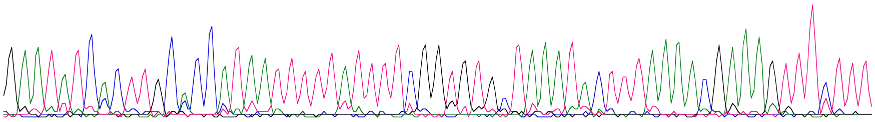

GAATATCACTTGCACCATAATTTTTATTTTCGGTGTGCTAAATACTTTAAAACGAAAGTTTCTTT

1370 1380 1390 1400 1410 1420 1430

TTTTTTTTCATGTAAACACCATTGTAGTATTAAAATCATCTTCTCTCGGAGAGCTGAAATGAATG

2727.seq(1>3519) ttttttttcatgtaaacaccattgtagtattaaaatcatcttctctcggagagctgaaatgaatg
KIT110-1-2651-CKIT-D-SEQR1-F02.a(1>663)

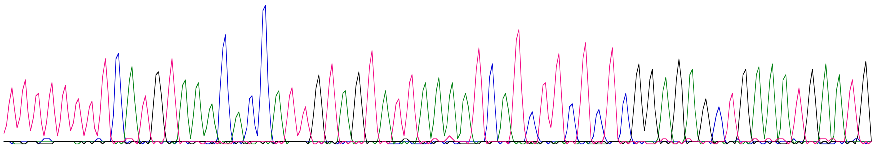

TTTTTTTTCATGTAAACACCATTGTAGTATTAAAATCATCTTCTCTCGGAGAGCTGAAATGAATG

1440 1450 1460 1470 1480 1490

GCTGTTGCTGTCTTTCCTTTTCTCCCCCAACAGTGTATTCACAGAGATTTGGCAGCCAGGAATAT

2727.seq(1>3519) gctgttgctgtctttccttttctcccccaacagtgtattcacagagatttggcagccaggaatat
KIT110-1-2651-CKIT-D-SEQR1-F02.a(1>663)

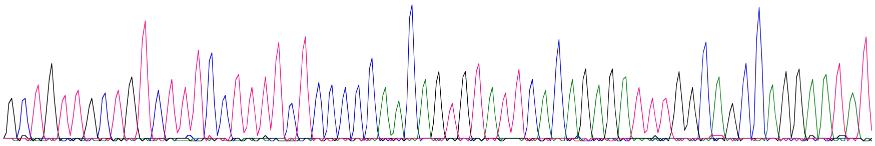

GCTGTTGCTGTCTTTCCTTTTCTCCCCCAACAGTGTATTCACAGAGATTTGGCAGCCAGGAATAT

1500 1510 1520 1530 1540 1550 1560

CCTCCTCACTCACGGGCGGATCACAAAGATTTGCGATTTCGGGCTAGCCAGAGACATCAGGAATG

2727.seq(1>3519) cctcctcactcacgggcggatcacaaagatttgcgatttcgggctagccagagacatcaggaatg
KIT110-1-2651-CKIT-D-SEQR1-F02.a(1>663)

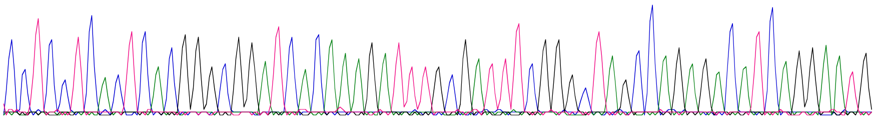

CCTCCTCACTCACGGGCGGATCACAAAGATTTGCGATTTCGGGCTAGCCAGAGACATCAGGAATG

1570 1580 1590 1600 1610 1620

ATTCGAATTACGTKGTCAAAGGAAATRTGAGTACCTTTCTCCATCTCATGAGTCTACCCAGGGTG

2727.seq(1>3519) attcgaattacgttgtcaaaggaaatatgagtacctttctccatctcatgagtctacccagggtg
KIT110-1-2651-CKIT-D-SEQR1-F02.a(1>663)

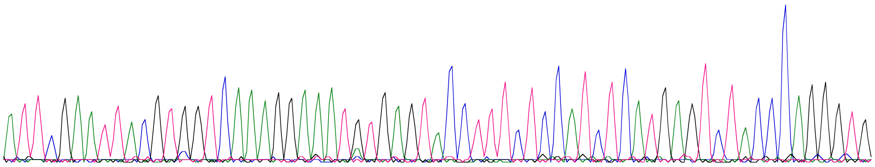

ATTCGAATTACGTGGTCAAAGGAAATGTGAGTACCTTTCTCCATCTCATGAGTCTACCCAGGGTG

KIT110-1-2651-CKIT-D-3INR2-F03.a(1>935) TGAGTACCTTTCTCCATCTCATGAGTCTACCCAGGGTG

1630 1640 1650 1660 1670 1680 1690

CTTTGGTATCCAGTCTTGATTCTAAATTGTTTTCTATGATCATTACAACTCCTACCTTGTAATGT

2727.seq(1>3519) ctttggtatccagtcttgattctaaattgttttctatgatcattacaactcctaccttgtaatgt
KIT110-1-2651-CKIT-D-SEQR1-F02.a(1>663)

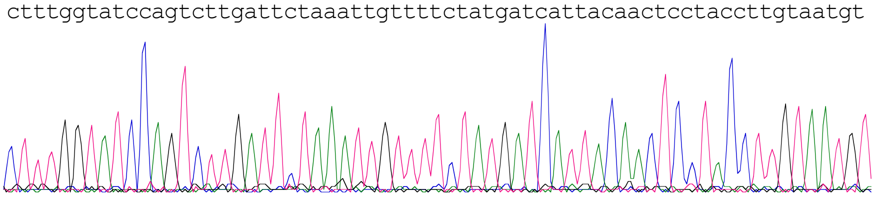

CTTTGGTATCCAGTCTTGATTCTAAATTGTTTTCTATGATCATTACAACTCCTACCTTGTAATGT

1630 1640 1650 1660 1670 1680 1690

CTTTGGTATCCAGTCTTGATTCTAAATTGTTTTCTATGATCATTACAACTCCTACCTTGTAATGT

KIT110-1-2651-CKIT-D-3INR2-F03.a(1>935) ← CTTTGGTATCCAGTCTTGATTCTAAATTGTTTTCTATGATCATTACAACTCCTACCTTGTAATGT

1700 1710 1720 1730 1740 1750

CACATGTTAGTACCACTAAGGCTTGTTAATAGAATTTTTAGCTATAATTGTATAATTGGGGGTGT

2727.seq(1>3519) → cacatgttagtaccactaaggcttgttaatagaatttttagctataattgtataattgggggtgt

KIT110-1-2651-CKIT-D-SEQR1-F02.a(1>663) ←

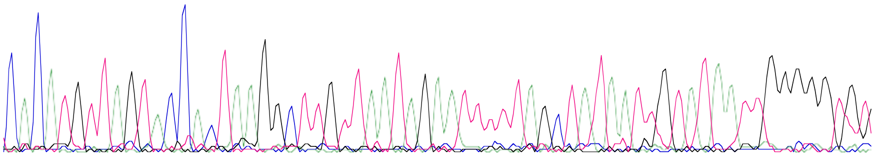

CACATGTTAGTACCACTAAGGCTTGTTAATAGAATTTTTAGCTATAATTGTATAATTGGGGGTGT

KIT110-1-2651-CKIT-D-3INR2-F03.a(1>935) ← CACATGTTAGTACCACTAAGGCTTGTTAATAGAATTTTTAGCTATAATTGTATAATTGGGGGTGT

1760 1770 1780 1790 1800 1810 1820

GCGAAATAAACAAAAATAGCCTTAATTTTTCACGATAGGCTGAAACTAGCAATCCATTTGATTTT

2727.seq(1>3519) → gcgaaataaacaaaaatagccttaatttttcacgataggctgaaactagcaatccatttgatttt

KIT110-1-2651-CKIT-D-SEQR1-F02.a(1>663) ←

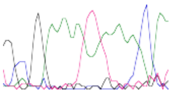

GCGAAATAAACAA

KIT110-1-2651-CKIT-D-3INR2-F03.a(1>935) ← GCGAAATAAACAAAAATAGCCTTAATTTTTCACGATAGGCTGAAACTAGCAATCCATTTGATTTT

1830 1840 1850 1860 1870 1880

GCTCAACTATAACAAATGTAGACCTGAAGGTCACGGTAGGCTTTCTCCCCGCTTCGTTTGAAATA

2727.seq(1>3519) → gctcaactataacaaatgtagacctgaaggtcacggtaggctttctccccgcttcgtttgaaata

KIT110-1-2651-CKIT-D-3INR2-F03.a(1>935) ← GCTCAACTATAACAAATGTAGACCTGAAGGTCACGGTAGGCTTTCTCCCCGCTTCGTTTGAAATA

1890 1900 1910 1920 1930 1940 1950

CTATGTGGAAATGACTACAGTCTAAGATGGTGTTAGAGTTAGCTGTATGCAATACTAGGATGCTT

2727.seq(1>3519) → ctatgtggaaatgactacagtctaagatggtgttagagttagctgtatgcaatactaggatgctt

KIT110-1-2651-CKIT-D-3INR2-F03.a(1>935) ← CTATGTGGAAATGACTACAGTCTAAGATGGTGTTAGAGTTAGCTGTATGCAATACTAGGATGCTT

1960 1970 1980 1990 2000 2010

GGGTCTCTTAATCAATATAGGGCTATTAAGTAGATTCATAAGTAATGGTTGGTTTGTGGGACACA

2727.seq(1>3519) → gggtctcttaatcaatatagggctattaagtagattcataagtaatggttggtttgtgggacaca

KIT110-1-2651-CKIT-D-3INR2-F03.a(1>935) ← GGGTCTCTTAATCAATATAGGGCTATTAAGTAGATTCATAAGTAATGGTTGGTTTGTGGGACACA

2020 2030 2040 2050 2060 2070 2080

GTGAAGTTTTCAAAGTGCCAGCCTTCTGTAACCTCAGAACAAACCTTAGAACATGTTGTCTTCCA

2727.seq(1>3519) → gtgaagttttcaaagtgccagccttctgtaacctcagaacaaaccttagaacatgttgtcttcca

KIT110-1-2651-CKIT-D-3INR2-F03.a(1>935) ← GTGAAGTTTTCAAAGTGCCAGCCTTCTGTAACCTCAGAACAAACCTTAGAACATGTTGTCTTCCA

2090 2100 2110 2120 2130 2140

ATGTTTATAGTGGAAGAAACTAAGGTCCAGTATAGTTCAGTGATTTATATCTGAGATAGCTAGTC

2727.seq(1>3519) → atgtttatagtggaagaaactaaggtccagtatagttcagtgatttatatctgagatagctagtc

KIT110-1-2651-CKIT-D-3INR2-F03.a(1>935) ← ATGTTTATAGTGGAAGAAACTAAGGTCCAGTATAGTTCAGTGATTTATATCTGAGATAGCTAGTC

2150 2160 2170 2180 2190 2200 2210

AGCAGGGTCCACTGGGTAGGGAAGGCCATAACCTCCTAGATATATATTCTTAGGTGCGTTTTTCC

2727.seq(1>3519) → agcagggtccactgggtagggaaggccataacctcctagatatatattcttaggtgcgtttttcc

KIT110-1-2651-CKIT-D-3INR2-F03.a(1>935) ← AGCAGGGTCCACTGGGTAGGGAAGGCCATAACCTCCTAGATATATATTCTTAGGTGCGTTTTTCC

```
                                                        2220      2230      2240      2250      2260      2270
                                              CCTCAGCCCATTTGAACAGTCCATATTTATTCCTGGAGGTCAATTTTTGTGTCTGTCGTGATAAC
2727.seq(1>3519)                          →   cctcagcccatttgaacagtccatatttattcctggaggtcaatttttgtgtctgtcgtgataac
KIT110-1-2651-CKIT-D-3INR2-F03.a(1>935)   ←   CCTCAGCCCATTTGAACAGTCCATATTTATTCCTGGAGGTCAATTTTTGTGTCTGTCGTGATAAC

                                                  2280      2290      2300      2310      2320      2330      2340
                                              GCAGTTCCTTCTCGTTCATCACCGTGGCGGCAGCATCTCTGTTGGTAGGCTGTGCATGACTTGGT
2727.seq(1>3519)                          →   gcagttccttctcgttcatcaccgtggcggcagcatctctgttggtaggctgtgcatgacttggt
KIT110-1-2651-CKIT-D-3INR2-F03.a(1>935)   ←   GCAGTTCCTTCTCGTTCATCACCGTGGCGGCAGCATCTCTGTTGGTAGGCTGTGCATGACTTGGT

                                                        2350      2360      2370      2380      2390      2400
                                              TTTGTAAGGAAGGGTATTGCTGGCTTTATATCCTGGCTCAGTCATACAGTTCTGAAGAGACTGTC
2727.seq(1>3519)                          →   tttgtaaggaagggtattgctggctttatatcctggctcagtcatacagttctgaagagactgtc
KIT110-1-2651-CKIT-D-3INR2-F03.a(1>935)   ←   TTTGTAAGGAAGGGTATTGCTGGCTTTATATCCTGGCTCAGTCATACAGTTCTGAAGAGACTGTC

                                                  2410      2420      2430      2440      2450      2460      2470
                                              AAGGTCAGATTTTTTTTTTCCAGGAGGTTTGATGAAATCAACTTCCTGGCTTAATATTCGAAGGA
2727.seq(1>3519)                          →   aaggtcagattttttttttccaggaggtttgatgaaatcaacttcctggcttaatattcgaagga
KIT110-1-2651-CKIT-D-3INR2-F03.a(1>935)   ←   AAGGTCAGATTTTTTTTTTCCAGGAGGTTTGATGAAATCAACTTCCTGGCTTAATATTCGAAGGA

                                                        2480      2490      2500      2510      2520      2530
                                              AGATGGGAAATGCTTCAGTATTCCAGGATAAAGGAGCAAAATAAGGAAATGGggtgtctgggccc
2727.seq(1>3519)                          →   agatgggaaatgcttcagtattccaggataaaggagcaaaataaggaaatggggtgtctgggccc
KIT110-1-2651-CKIT-D-3INR2-F03.a(1>935)   ←   AGATGGGAAATGCTTCAGTATTCCAGGATAAAGGAGCAAAATAAGGAAATGG
```

KIT $^{Dup/+}$

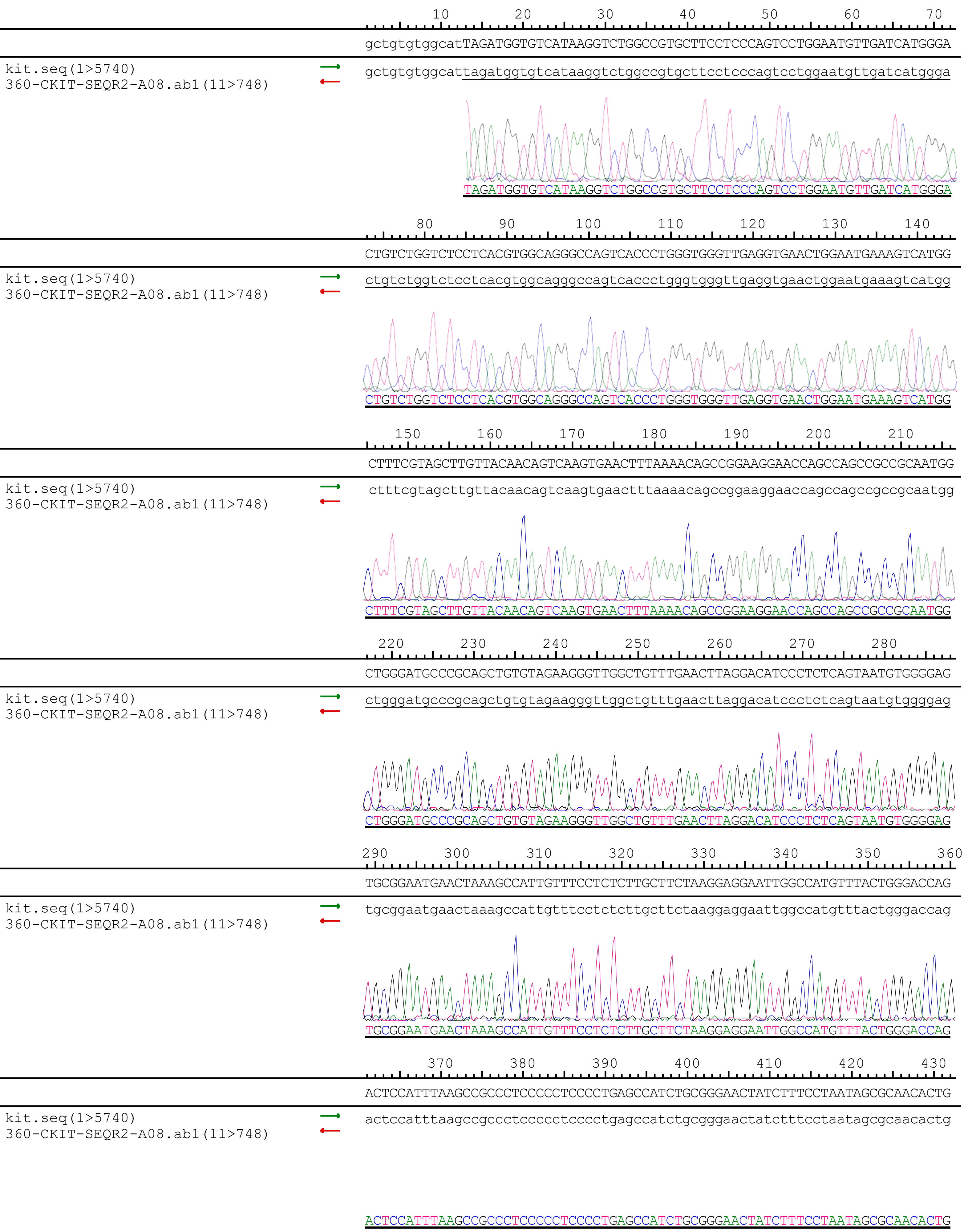
10 20 30 40 50 60 70
gctgtgtggcatTAGATGGTGTCATAAGGTCTGGCCGTGCTTCCTCCCAGTCCTGGAATGTTGATCATGGGA
kit.seq(1>5740)
360-CKIT-SEQR2-A08.ab1(11>748)
gctgtgtggcattagatggtgtcataaggtctggccgtgcttcctcccagtcctggaatgttgatcatggga
TAGATGGTGTCATAAGGTCTGGCCGTGCTTCCTCCCAGTCCTGGAATGTTGATCATGGGA
80 90 100 110 120 130 140
CTGTCTGGTCTCCTCACGTGGCAGGGCCAGTCACCCTGGGTGGGTTGAGGTGAACTGGAATGAAAGTCATGG
kit.seq(1>5740)
360-CKIT-SEQR2-A08.ab1(11>748)
ctgtctggtctcctcacgtggcagggccagtcaccctgggtgggttgaggtgaactggaatgaaagtcatgg
CTGTCTGGTCTCCTCACGTGGCAGGGCCAGTCACCCTGGGTGGGTTGAGGTGAACTGGAATGAAAGTCATGG
150 160 170 180 190 200 210
CTTTCGTAGCTTGTTACAACAGTCAAGTGAACTTTAAAACAGCCGGAAGGAACCAGCCAGCCGCCGCAATGG
kit.seq(1>5740)
360-CKIT-SEQR2-A08.ab1(11>748)
ctttcgtagcttgttacaacagtcaagtgaactttaaaacagccggaaggaaccagccagccgccgcaatgg
CTTTCGTAGCTTGTTACAACAGTCAAGTGAACTTTAAAACAGCCGGAAGGAACCAGCCAGCCGCCGCAATGG
220 230 240 250 260 270 280
CTGGGATGCCCGCAGCTGTGTAGAAGGGTTGGCTGTTTGAACTTAGGACATCCCTCTCAGTAATGTGGGGAG
kit.seq(1>5740)
360-CKIT-SEQR2-A08.ab1(11>748)
ctgggatgcccgcagctgtgtagaagggttggctgtttgaacttaggacatccctctcagtaatgtggggag
CTGGGATGCCCGCAGCTGTGTAGAAGGGTTGGCTGTTTGAACTTAGGACATCCCTCTCAGTAATGTGGGGAG
290 300 310 320 330 340 350 360
TGCGGAATGAACTAAAGCCATTGTTTCCTCTCTTGCTTCTAAGGAGGAATTGGCCATGTTTACTGGGACCAG
kit.seq(1>5740)
360-CKIT-SEQR2-A08.ab1(11>748)
tgcggaatgaactaaagccattgtttcctctcttgcttctaaggaggaattggccatgtttactgggaccag
TGCGGAATGAACTAAAGCCATTGTTTCCTCTCTTGCTTCTAAGGAGGAATTGGCCATGTTTACTGGGACCAG
370 380 390 400 410 420 430
ACTCCATTTAAGCCGCCCTCCCCCTCCCCTGAGCCATCTGCGGGAACTATCTTTCCTAATAGCGCAACACTG
kit.seq(1>5740)
360-CKIT-SEQR2-A08.ab1(11>748)
actccatttaagccgccctccccctcccctgagccatctgcgggaactatctttcctaatagcgcaacactg
ACTCCATTTAAGCCGCCCTCCCCCTCCCCTGAGCCATCTGCGGGAACTATCTTTCCTAATAGCGCAACACTG

440 450 460 470 480 490 500

ATCTTTTCGAGTTAGCACTTGTTCTGAGGGGCATCAAGGCACATTTCAAATGAAACGCCACTTTCCACCCAA

kit.seq(1>5740)
360-CKIT-SEQR2-A08.ab1(11>748)

atcttttcgagttagcacttgttctgaggggcatcaaggcacatttcaaatgaaacgccactttccacccaa

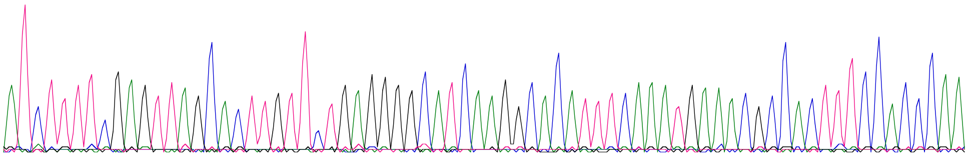

ATCTTTTCGAGTTAGCACTTGTTCTGAGGGGCATCAAGGCACATTTCAAATGAAACGCCACTTTCCACCCAA

510 520 530 540 550 560 570

GTGCTTTTGATTCCTGACAAACCACCTTTGGAAGAGCCATGGGTGCCACTGCAAATTACACCAGCCCTTGGG

kit.seq(1>5740)
360-CKIT-SEQR2-A08.ab1(11>748)

gtgcttttgattcctgacaaaccacctttggaagagccatgggtgccactgcaaattacaccagcccttggg

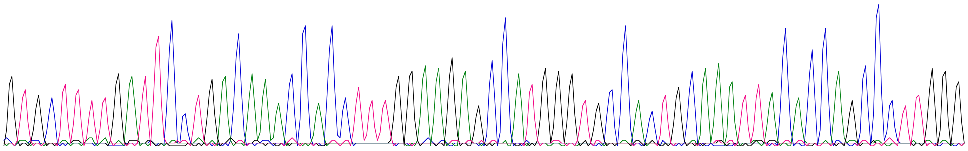

GTGCTTTTGATTCCTGACAAACCACCTTTGGAAGAGCCATGGGTGCCACTGCAAATTACACCAGCCCTTGGG

580 590 600 610 620 630 640

TTGCAGTTGGCTCTAGGGATCCTCAGATCTTCTTCACAGGCTAGCTAGTACTTGCTTTCAGACACACCCCAG

kit.seq(1>5740)
360-CKIT-SEQR2-A08.ab1(11>748)

ttgcagttggctctagggatcctcagatcttcttcacaggctagctagtacttgctttcagacacaccccag

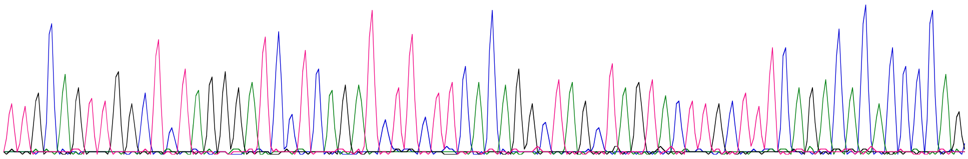

TTGCAGTTGGCTCTAGGGATCCTCAGATCTTCTTCACAGGCTAGCTAGTACTTGCTTTCAGACACACCCCAG

360-CKIT-SEQR1-A07.ab1(1>825)

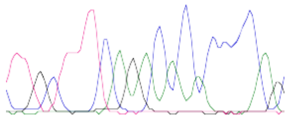

TTGCTTTCAGACACACCCCAG

650 660 670 680 690 700 710 720

CCGAAGAGAAACATTGCAGGCAGTGTTGTACCTCATTCTCGAAATGATTTACCTGGGGAAACTGAGGTATAA

kit.seq(1>5740)
360-CKIT-SEQR2-A08.ab1(11>748)

ccgaagagaaacattgcaggcagtgttgtacctcattctcgaaatgatttacctggggaaactgaggtataa

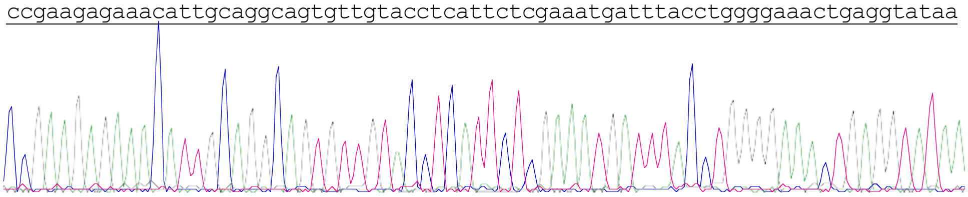

CCGAAGAGAAACATTGCAGGCAGTGTTGTACCTCATTCTCGAAATGATTTACCTGGGGAAACTGAGGTATAA

360-CKIT-SEQR1-A07.ab1(1>825)

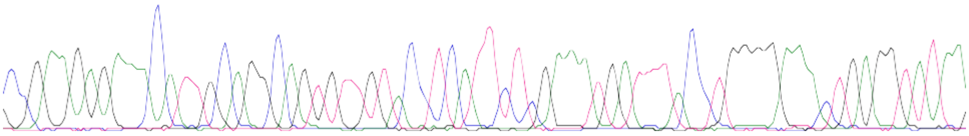

CCGAAGAGAAACATTGCAGGCAGTGTTGTACCTCATTCTCGAAATGATTTACCTGGGGAAACTGAGGTATAA

730 740 750 760 770 780 790

GCCCCATCTTGATTTTTGACCACTCTTCCTTGTCTTCAGGATTCTGCAACAGTTGAGCGGGCCCCATTTGCT

kit.seq(1>5740)
360-CKIT-SEQR2-A08.ab1(11>748)

gccccatcttgatttttgaccactcttccttgtcttcaggattctgcaacagttgagcgggccccatttgct

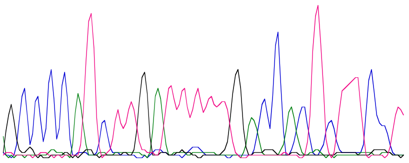

GCCCCATCTTGATTTTTGACCACTCTTCCT

730 740 750 760 770 780 790

GCCCCATCTTGATTTTTGACCACTCTTCCTTGTCTTCAGGATTCTGCAACAGTTGAGCGGGCCCCATTTGCT

360-CKIT-SEQR1-A07.ab1(1>825)

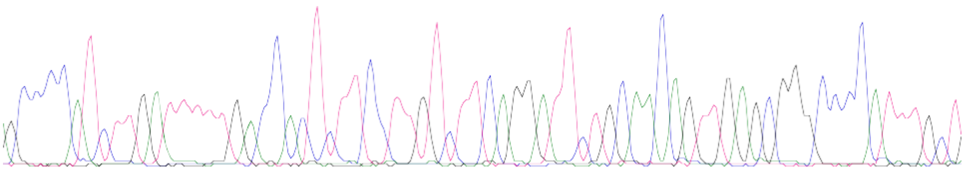

GCCCCATCTTGATTTTTGACCACTCTTCCTTGTCTTCAGGATTCTGCAACAGTTGAGCGGGCCCCATTTGCT

800 810 820 830 840 850 860

GGGTTTTGTCACCAAGCCGCTGTATTTGACCGTGGGCTCTTTCTCTGCAGATTTACTCCAACTTGGCAAACT

kit.seq(1>5740) gggttttgtcaccaagccgctgtatttgaccgtgggctctttctctgcagatttactccaacttggcaaact
360-CKIT-SEQR1-A07.ab1(1>825)

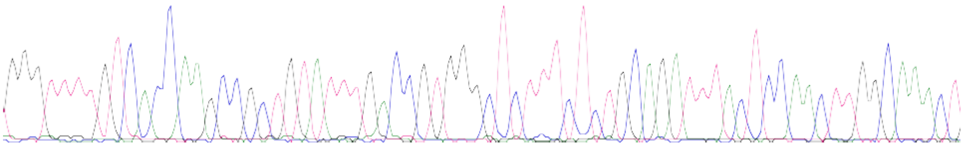

GGGTTTTGTCACCAAGCCGCTGTATTTGACCGTGGGCTCTTTCTCTGCAGATTTACTCCAACTTGGCAAACT

870 880 890 900 910 920 930

GCAACCCCAACCCAGAGAACCCCGTGGTGGTGGACCATTCCGTGAGGGTCAACTCGGTGGGCAGCAGCGCCT

kit.seq(1>5740) gcaaccccaacccagagaaccccgtggtggtggaccattccgtgagggtcaactcggtgggcagcagcgcct
360-CKIT-SEQR1-A07.ab1(1>825)

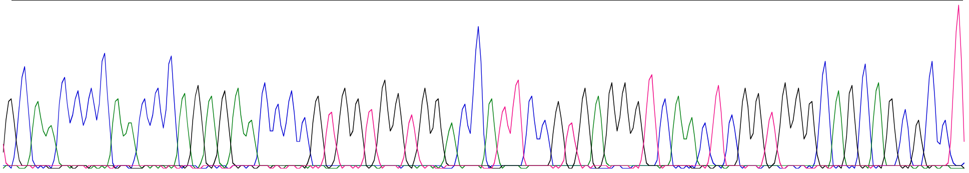

GCAACCCCAACCCAGAGAACCCCGTGGTGGTGGACCATTCCGTGAGGGTCAACTCGGTGGGCAGCAGCGCCT

940 950 960 970 980 990 1000

CTTCTACGCAGCCATTGCTCGTGCACGAAGATGCCGCCACGAACTTCTCTCTGTTAAAGCAAGCAGGAGACG

kit.seq(1>5740) cttctacgcagccattgctcgtgcacgaagatgccgccacgaacttctctctgttaaagcaagcaggagacg
360-CKIT-SEQR1-A07.ab1(1>825)

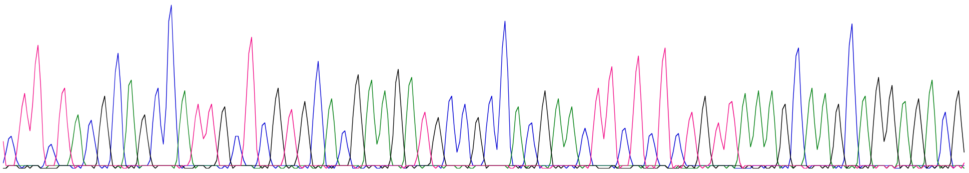

CTTCTACGCAGCCATTGCTCGTGCACGAAGATGCCGCCACGAACTTCTCTCTGTTAAAGCAAGCAGGAGACG

1010 1020 1030 1040 1050 1060 1070 1080

TGGAAGAAAACCCCGGTCCTATGAGAGGCGCTCGCGGCGCCTGGGATCTGCTCTGCGTCCTGTTGGTCCTGC

kit.seq(1>5740) tggaagaaaaccccggtcctatgagaggcgctcgcggcgcctgggatctgctctgcgtcctgttggtcctgc
360-CKIT-SEQR1-A07.ab1(1>825)

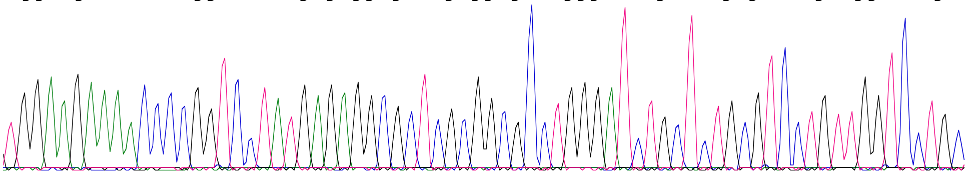

TGGAAGAAAACCCCGGTCCTATGAGAGGCGCTCGCGGCGCCTGGGATCTGCTCTGCGTCCTGTTGGTCCTGC

1090 1100 1110 1120 1130 1140 1150

TCCGTGGCCAGACAGCCACGTCTCAGCCATCTGCAAGTCCAGGGGAGCCGTCTCCGCCATCCATCCATCCAG

kit.seq(1>5740) tccgtggccagacagccacgtctcagccatctgcaagtccaggggagccgtctccgccatccatccatccag
360-CKIT-SEQR1-A07.ab1(1>825)

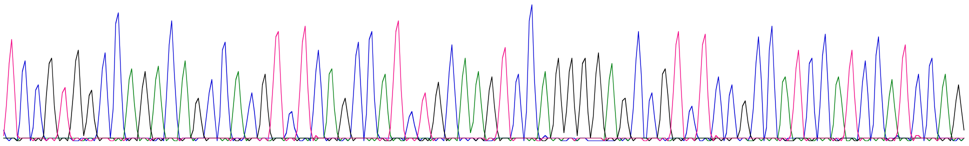

TCCGTGGCCAGACAGCCACGTCTCAGCCATCTGCAAGTCCAGGGGAGCCGTCTCCGCCATCCATCCATCCAG

1160 1170 1180 1190 1200 1210 1220

CACAATCAGAGTTAATAGTTGAAGCTGGCGACACCCTCAGCCTGACGTGCATTGATCCCGACTTTGTCAGAT

kit.seq(1>5740) cacaatcagagttaatagttgaagctggcgacaccctcagcctgacgtgcattgatcccgactttgtcagat

360-CKIT-SEQR1-A07.ab1(1>825)

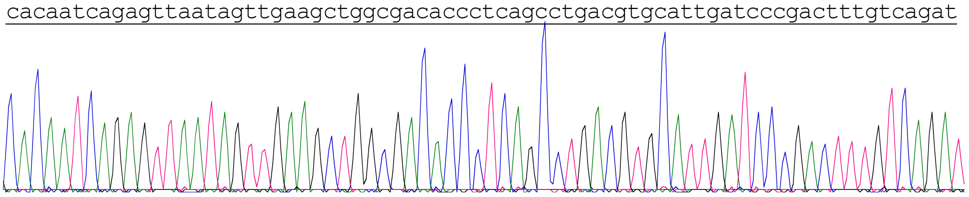

CACAATCAGAGTTAATAGTTGAAGCTGGCGACACCCTCAGCCTGACGTGCATTGATCCCGACTTTGTCAGAT

1230 1240 1250 1260 1270 1280 1290

GGACTTTCAAGACCTATTTCAATGAAATGGTTGAGAATAAAAAAAATGAATGGATCCAGGAAAAAGCCGAGG

kit.seq(1>5740) ggactttcaagacctatttcaatgaaatggttgagaataaaaaaaatgaatggatccaggaaaaagccgagg

360-CKIT-SEQR1-A07.ab1(1>825)

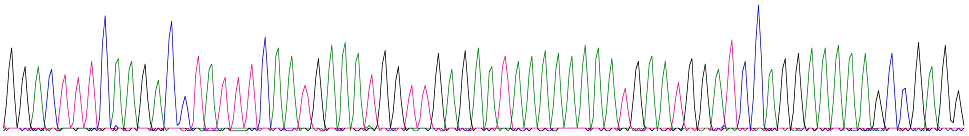

GGACTTTCAAGACCTATTTCAATGAAATGGTTGAGAATAAAAAAAATGAATGGATCCAGGAAAAAGCCGAGG

1300 1310 1320 1330 1340 1350 1360

CCACTCGCACGGGCACATACACGTGCAGCAACAGCAATGGCCTCACGAGTTCTATTTACGTGTTTGTTAGAG

kit.seq(1>5740) ccactcgcacgggcacatacacgtgcagcaacagcaatggcctcacgagttctatttacgtgtttgttagag

360-CKIT-SEQR1-A07.ab1(1>825)

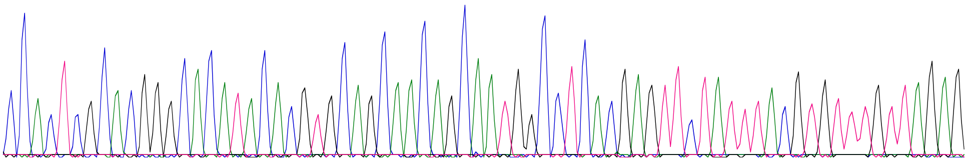

CCACTCGCACGGGCACATACACGTGCAGCAACAGCAATGGCCTCACGAGTTCTATTTACGTGTTTGTTAGAG

1370 1380 1390 1400 1410 1420 1430 1440

ATCCTGCCAAACTTTTCCTGGTTGGCCTTCCCTTGTTTGGCAAAGAAGACAGCGACGCGCTGGTCCGCTGCC

kit.seq(1>5740) atcctgccaaacttttcctggttggccttcccttgtttggcaaagaagacagcgacgcgctggtccgctgcc

360-CKIT-SEQR1-A07.ab1(1>825)

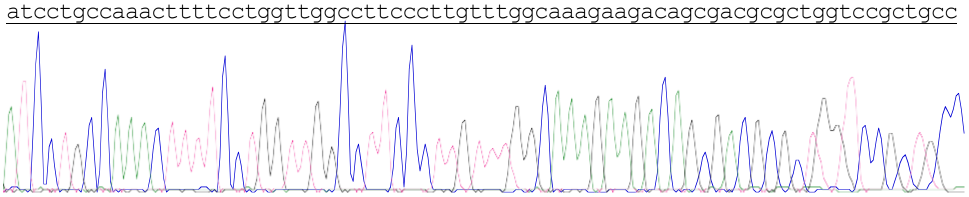

ATCCTGCCAAACTTTTCCTGGTTGGCCTTCCCTTGTTTGGCAAAGAAGACAGCGACGCGCTGGTCCGCTGCC

360-CKIT-SEQF1-A09.ab1(13>865)

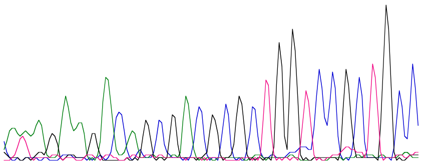

AAAGAAGACAGCGACGCGCTGGTCCGCTGCC

1450 1460 1470 1480 1490 1500 1510

CTCTGACAGACCCACAGGTGTCCAATTATTCCCTCATCGAGTGTGATGGGAAATCTCTCCCCACGGACCTGA

kit.seq(1>5740) ctctgacagacccacaggtgtccaattattccctcatcgagtgtgatgggaaatctctccccacggacctga

360-CKIT-SEQR1-A07.ab1(1>825)

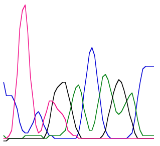

CTCTGACAGACC

360-CKIT-SEQF1-A09.ab1(13>865)

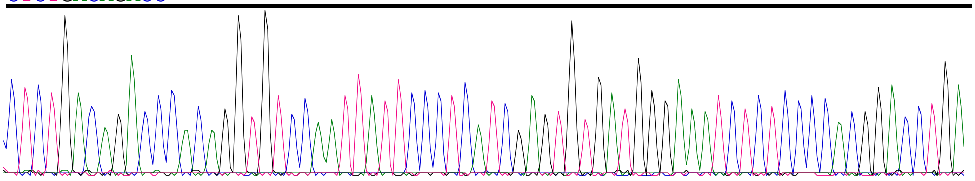

CTCTGACAGACCCACAGGTGTCCAATTATTCCCTCATCGAGTGTGATGGGAAATCTCTCCCCACGGACCTGA

1520 1530 1540 1550 1560 1570 1580

CGTTTGTCCCAAACCCCAAGGCTGGCATCACCATCAAAAACGTGAAGCGCGCCTACCACCGGCTCTGTGTCC

kit.seq(1>5740) → cgtttgtcccaaaccccaaggctggcatcaccatcaaaaacgtgaagcgcgcctaccaccggctctgtgtcc
360-CKIT-SEQF1-A09.ab1(13>865) →

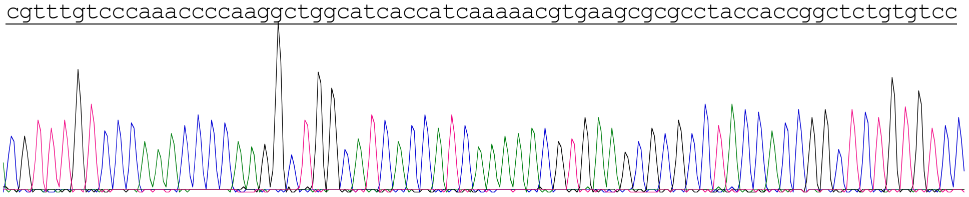

CGTTTGTCCCAAACCCCAAGGCTGGCATCACCATCAAAAACGTGAAGCGCGCCTACCACCGGCTCTGTGTCC

1590 1600 1610 1620 1630 1640 1650

GCTGTGCTGCTCAGCGTGACGGTACATGGCTGCATTCTGACAAATTCACCCTCAAAGTGCGGGCAGCCATCA

kit.seq(1>5740) → gctgtgctgctcagcgtgacggtacatggctgcattctgacaaattcaccctcaaagtgcgggcagccatca
360-CKIT-SEQF1-A09.ab1(13>865) →

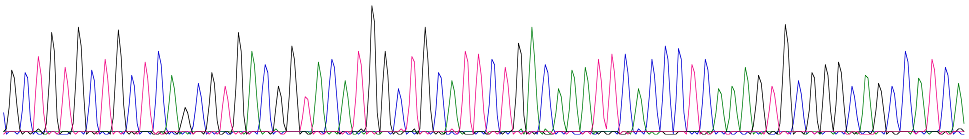

GCTGTGCTGCTCAGCGTGACGGTACATGGCTGCATTCTGACAAATTCACCCTCAAAGTGCGGGCAGCCATCA

1660 1670 1680 1690 1700 1710 1720

AGGCTATCCCTGTTGTGTCTGTGCCTGAAACAAGTCACCTCCTCAAGAAAGGGGACACATTTACGGTGGTGT

kit.seq(1>5740) → aggctatccctgttgtgtctgtgcctgaaacaagtcacctcctcaagaaaggggacacatttacggtggtgt
360-CKIT-SEQF1-A09.ab1(13>865) →

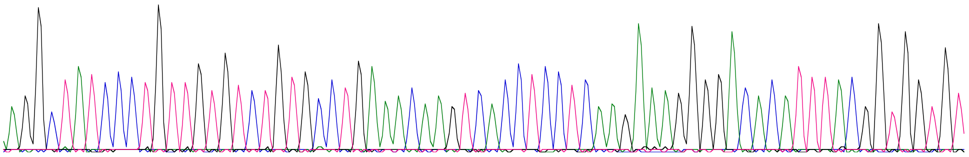

AGGCTATCCCTGTTGTGTCTGTGCCTGAAACAAGTCACCTCCTCAAGAAAGGGGACACATTTACGGTGGTGT

1730 1740 1750 1760 1770 1780 1790 1800

GCACCATAAAAGATGTGTCTACATCCGTGAACTCCATGTGGCTAAAGATGAACCCTCAGCCTCAGCACATAG

kit.seq(1>5740) → gcaccataaaagatgtgtctacatccgtgaactccatgtggctaaagatgaaccctcagcctcagcacatag
360-CKIT-SEQF1-A09.ab1(13>865) →

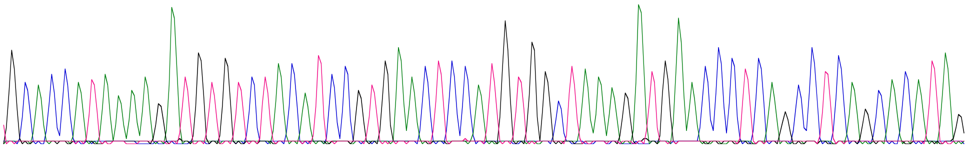

GCACCATAAAAGATGTGTCTACATCCGTGAACTCCATGTGGCTAAAGATGAACCCTCAGCCTCAGCACATAG

1810 1820 1830 1840 1850 1860 1870

CCCAGGTAAAGCACAATAGCTGGCACCGGGGTGACTTCAATTATGAACGCCAGGAGACGCTGACTATCAGCT

kit.seq(1>5740) → cccaggtaaagcacaatagctggcaccggggtgacttcaattatgaacgccaggagacgctgactatcagct
360-CKIT-SEQF1-A09.ab1(13>865) →

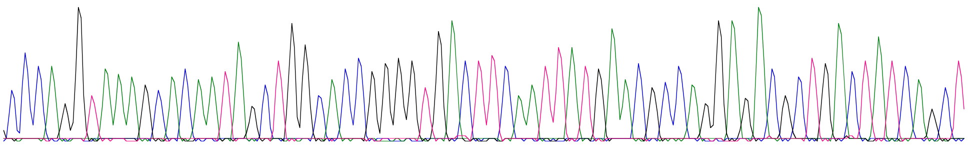

CCCAGGTAAAGCACAATAGCTGGCACCGGGGTGACTTCAATTATGAACGCCAGGAGACGCTGACTATCAGCT

1880 1890 1900 1910 1920 1930 1940

CGGCAAGAGTTGACGATTCTGGAGTGTTCATGTGTTATGCCAATAATACTTTTGGATCAGCAAATGTCACAA

kit.seq(1>5740) → cggcaagagttgacgattctggagtgttcatgtgttatgccaataatacttttggatcagcaaatgtcacaa
360-CKIT-SEQF1-A09.ab1(13>865) →

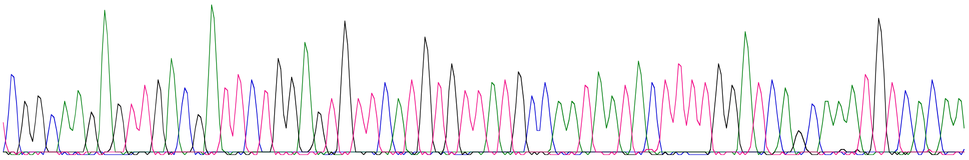

CGGCAAGAGTTGACGATTCTGGAGTGTTCATGTGTTATGCCAATAATACTTTTGGATCAGCAAATGTCACAA

1950 1960 1970 1980 1990 2000 2010

CAACCTTGAAAGTAGTAGAAAAAGGATTCATCAACATCTCCCCTGTGAAGAACACTACAGTATTTGTAACCG

kit.seq(1>5740) caaccttgaaagtagtagaaaaaggattcatcaacatctcccctgtgaagaacactacagtatttgtaaccg

360-CKIT-SEQF1-A09.ab1(13>865)

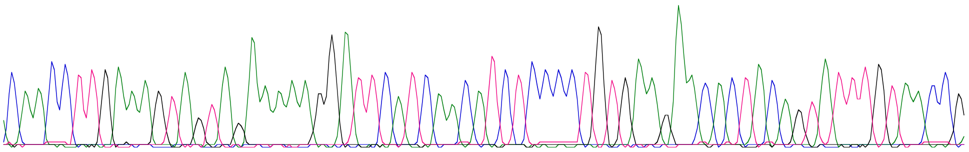

CAACCTTGAAAGTAGTAGAAAAAGGATTCATCAACATCTCCCCTGTGAAGAACACTACAGTATTTGTAACCG

2020 2030 2040 2050 2060 2070 2080

ATGGAGAAAACGTAGATTTGGTTGTTGAATACGAGGCCTACCCCAAACCCGAGCACCAGCAGTGGATATATA

kit.seq(1>5740) atggagaaaacgtagatttggttgttgaatacgaggcctaccccaaacccgagcaccagcagtggatatata

360-CKIT-SEQF1-A09.ab1(13>865)

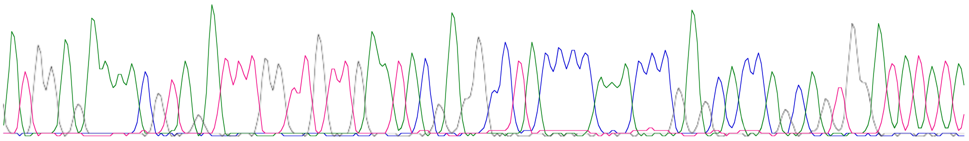

ATGGAGAAAACGTAGATTTGGTTGTTGAATACGAGGCCTACCCCAAACCCGAGCACCAGCAGTGGATATATA

2090 2100 2110 2120 2130 2140 2150 2160

TGAACAGGACCTCGGCTAACAAAGGGAAGGATTATGTCAAATCTGATAACAAAAGCAACATCAGATATGTGA

kit.seq(1>5740) tgaacaggacctcggctaacaaagggaaggattatgtcaaatctgataacaaaagcaacatcagatatgtga

360-CKIT-SEQF1-A09.ab1(13>865)

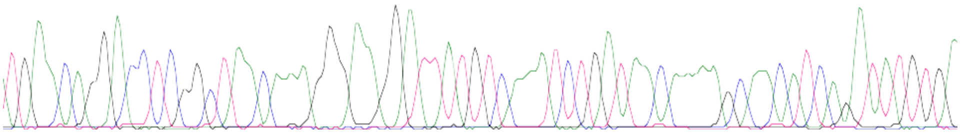

TGAACAGGACCTCGGCTAACAAAGGGAAGGATTATGTCAAATCTGATAACAAAAGCAACATCAGATATGTGA

2170 2180 2190 2200 2210 2220 2230

ACCAACTTCGCCTGACCAGATTAAAAGGCACAGAAGGAGGCACTTATACCTTTCTGGTGTCCAACTCTGATG

kit.seq(1>5740) accaacttcgcctgaccagattaaaaggcacagaaggaggcacttatacctttctggtgtccaactctgatg

360-CKIT-SEQF1-A09.ab1(13>865)

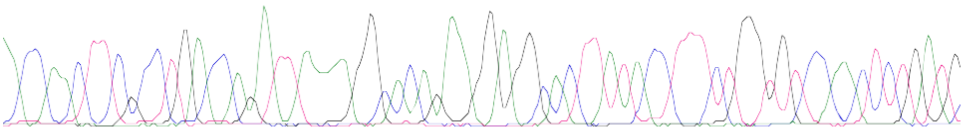

ACCAACTTCGCCTGACCAGATTAAAAGGCACAGAAGGAGGCACTTATACCTTTCTGGTGTCCAACTCTGATG

360-CKIT-SEQF2-A10.ab1(56>849)

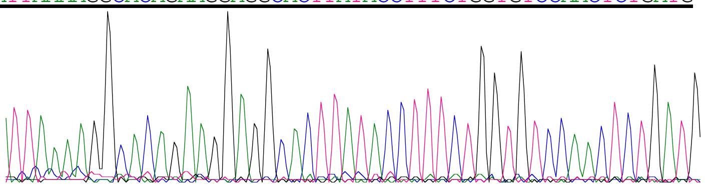

TTAAAAGGCACAGAAGGAGGCACTTATACCTTTCTGGTGTCCAACTCTGATG

2240 2250 2260 2270 2280 2290 2300

CCAGTGCTTCCGTGACATTCAACGTTTACGTGAACACAAAACCAGAAATCCTGACGTACGACAGGCTCATAA

kit.seq(1>5740) ccagtgcttccgtgacattcaacgtttacgtgaacacaaaaccagaaatcctgacgtacgacaggctcataa

360-CKIT-SEQF1-A09.ab1(13>865)

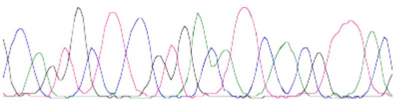

CCAGTGCTTCCGTGACATTCAACGTTTACG

360-CKIT-SEQF2-A10.ab1(56>849)

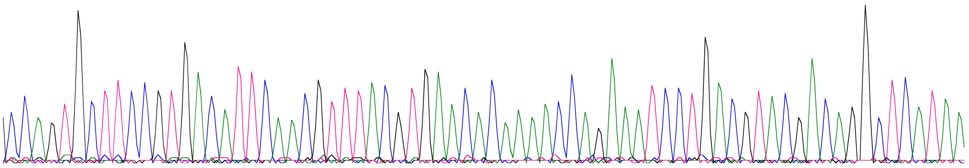

CCAGTGCTTCCGTGACATTCAACGTTTACGTGAACACAAAACCAGAAATCCTGACGTACGACAGGCTCATAA

2310 2320 2330 2340 2350 2360 2370

ATGGCATGCTCCAGTGTGTGGCAGAGGGATTCCCGGAGCCCACAATAGATTGGTATTTTTGTACAGGAGCAG

kit.seq(1>5740) atggcatgctccagtgtgtggcagagggattcccggagcccacaatagattggtatttttgtacaggagcag
360-CKIT-SEQF2-A10.ab1(56>849)

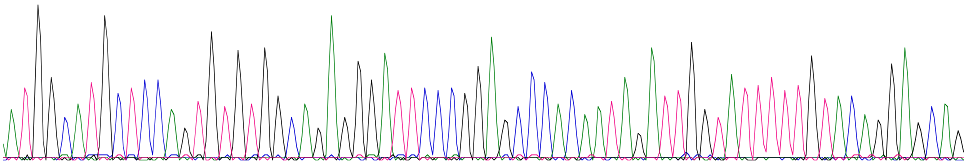

ATGGCATGCTCCAGTGTGTGGCAGAGGGATTCCCGGAGCCCACAATAGATTGGTATTTTTGTACAGGAGCAG

2380 2390 2400 2410 2420 2430 2440

AGCAAAGGTGTACCACTCCTGTCTCACCAGTGGACGTACAGGTCCAGAATGTATCTGTGTCACCATTTGGAA

kit.seq(1>5740) agcaaaggtgtaccactcctgtctcaccagtggacgtacaggtccagaatgtatctgtgtcaccatttggaa
360-CKIT-SEQF2-A10.ab1(56>849)

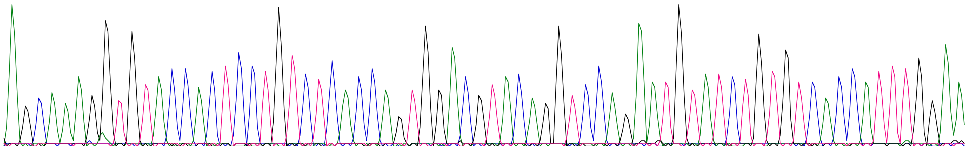

AGCAAAGGTGTACCACTCCTGTCTCACCAGTGGACGTACAGGTCCAGAATGTATCTGTGTCACCATTTGGAA

2450 2460 2470 2480 2490 2500 2510 2520

AACTGGTGGTTCAGAGTTCCATAGACTCCAGCGTCTTCCGGCACAACGGCACGGTGGAGTGTAAGGCCTCCA

kit.seq(1>5740) aactggtggttcagagttccatagactccagcgtcttccggcacaacggcacggtggagtgtaaggcctcca
360-CKIT-SEQF2-A10.ab1(56>849)

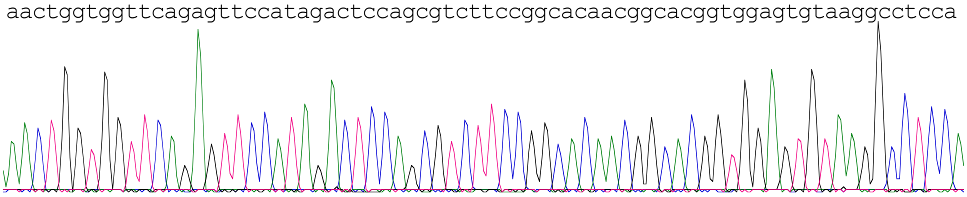

AACTGGTGGTTCAGAGTTCCATAGACTCCAGCGTCTTCCGGCACAACGGCACGGTGGAGTGTAAGGCCTCCA

2530 2540 2550 2560 2570 2580 2590

ACGATGTGGGCAAGAGTTCCGCCTTCTTTAACTTTGCATTTAAAGGTAACAACAAAGAGCAAATCCAGGCCC

kit.seq(1>5740) acgatgtgggcaagagttccgccttctttaactttgcatttaaaggtaacaacaaagagcaaatccaggccc
360-CKIT-SEQF2-A10.ab1(56>849)

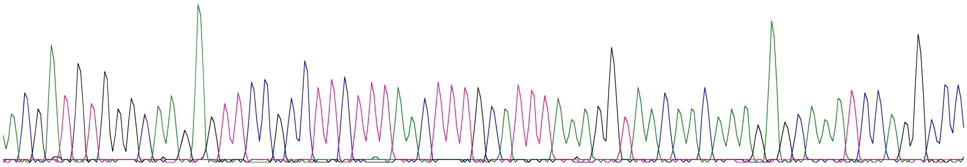

ACGATGTGGGCAAGAGTTCCGCCTTCTTTAACTTTGCATTTAAAGGTAACAACAAAGAGCAAATCCAGGCCC

2600 2610 2620 2630 2640 2650 2660

ACACTCTGTTCACGCCGCTGCTCATTGGCTTTGTGGTTGCAGCTGGCGCGATGGGGATCATTGTGATGGTGC

kit.seq(1>5740) acactctgttcacgccgctgctcattggctttgtggttgcagctggcgcgatggggatcattgtgatggtgc
360-CKIT-SEQF2-A10.ab1(56>849)

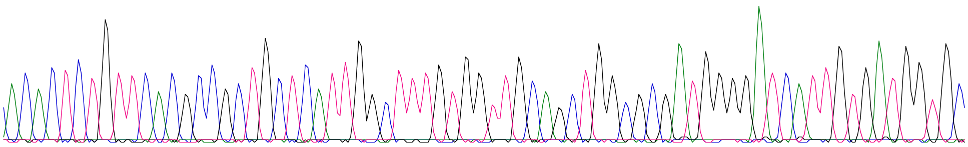

ACACTCTGTTCACGCCGCTGCTCATTGGCTTTGTGGTTGCAGCTGGCGCGATGGGGATCATTGTGATGGTGC

2670 2680 2690 2700 2710 2720 2730

TCACCTACAAATATTTGCAGAAACCCATGTATGAAGTACAATGGAAGGTTGTCGAGGAGATAAATGGAAACA

kit.seq(1>5740) tcacctacaaatatttgcagaaacccatgtatgaagtacaatggaaggttgtcgaggagataaatggaaaca
360-CKIT-SEQF2-A10.ab1(56>849)

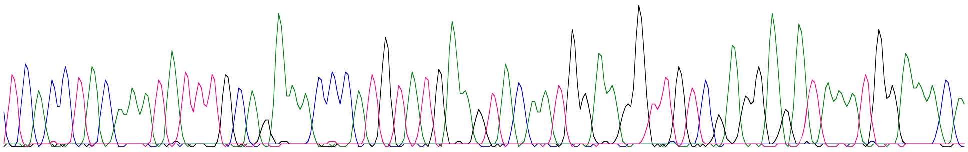

TCACCTACAAATATTTGCAGAAACCCATGTATGAAGTACAATGGAAGGTTGTCGAGGAGATAAATGGAAACA

2740 2750 2760 2770 2780 2790 2800

ATTATGTTTACATAGACCCGACGCAACTTCCTTATGATCACAAATGGGAGTTTCCCAGAAACAGGCTGAGTT

kit.seq(1>5740) attatgtttacatagacccgacgcaacttccttatgatcacaaatgggagtttcccagaaacaggctgagtt

360-CKIT-SEQF2-A10.ab1(56>849)

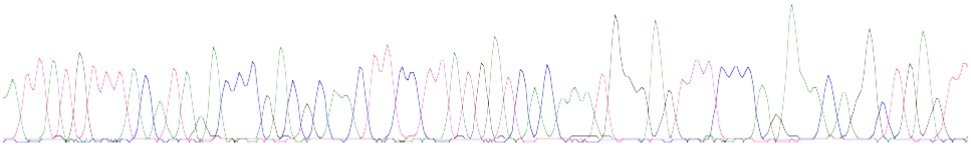

ATTATGTTTACATAGACCCGACGCAACTTCCTTATGATCACAAATGGGAGTTTCCCAGAAACAGGCTGAGTT

2810 2820 2830 2840 2850 2860 2870 2880

TTGGAAAGACATTGGGAGCTGGTGCCTTCGGGAAGGTCGTTGAGGCCACTGCATATGGCTTGATTAAGTCGG

kit.seq(1>5740) ttggaaagacattgggagctggtgccttcgggaaggtcgttgaggccactgcatatggcttgattaagtcgg

360-CKIT-SEQF2-A10.ab1(56>849)

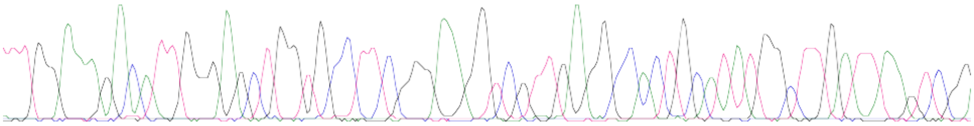

TTGGAAAGACATTGGGAGCTGGTGCCTTCGGGAAGGTCGTTGAGGCCACTGCATATGGCTTGATTAAGTCGG

360-CKIT-SEQF3-A11.ab1(14>947)

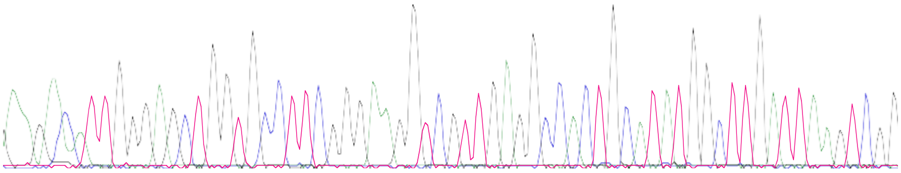

AAGACATTGGGAGCTGGTGCCTTCGGGAAGGTCGTTGAGGCCACTGCATATGGCTTGATTAAGTCGG

2890 2900 2910 2920 2930 2940 2950

ATGCTGCCATGACAGTTGCCGTGAAGATGCTCAAACCAAGTGCCCATTTAACAGAAAGAGAGGCCCTAATGT

kit.seq(1>5740) atgctgccatgacagttgccgtgaagatgctcaaaccaagtgcccatttaacagaaagagaggccctaatgt

360-CKIT-SEQF2-A10.ab1(56>849)

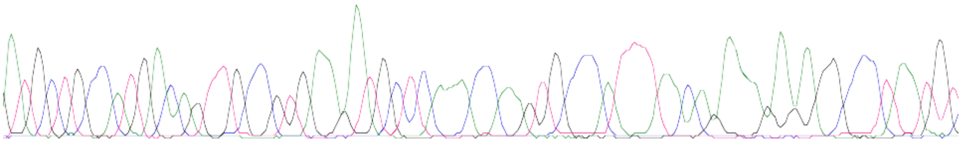

ATGCTGCCATGACAGTTGCCGTGAAGATGCTCAAACCAAGTGCCCATTTAACAGAAAGAGAGGCCCTAATGT

360-CKIT-SEQF3-A11.ab1(14>947)

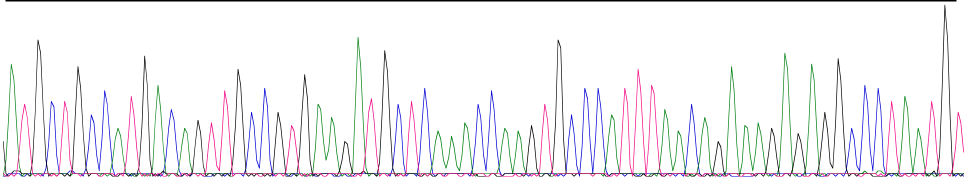

ATGCTGCCATGACAGTTGCCGTGAAGATGCTCAAACCAAGTGCCCATTTAACAGAAAGAGAGGCCCTAATGT

2960 2970 2980 2990 3000 3010 3020

CGGAACTGAAGGTCCTGAGCTACCTGGGCAATCACATGAATATTGTGAACCTGCTTGGCGCATGCACGGTGG

kit.seq(1>5740) cggaactgaaggtcctgagctacctgggcaatcacatgaatattgtgaacctgcttggcgcatgcacggtgg

360-CKIT-SEQF2-A10.ab1(56>849)

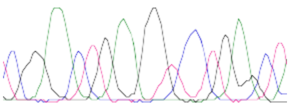

CGGAACTGAAGGTCCTGAGCTA

360-CKIT-SEQF3-A11.ab1(14>947)

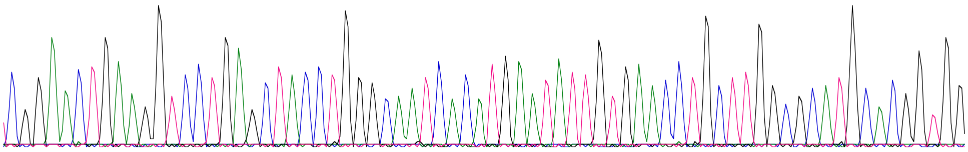

CGGAACTGAAGGTCCTGAGCTACCTGGGCAATCACATGAATATTGTGAACCTGCTTGGCGCATGCACGGTGG

3030 3040 3050 3060 3070 3080 3090

GAGGGCCCACCCTGGTCATTACAGAATATTGTTGCTATGGTGATCTTTTGAATTTTTTGAGAAGGAAGCGTG

kit.seq(1>5740) → gagggcccaccctggtcattacagaatattgttgctatggtgatcttttgaattttttgagaaggaagcgtg

360-CKIT-SEQF3-A11.ab1(14>947) →

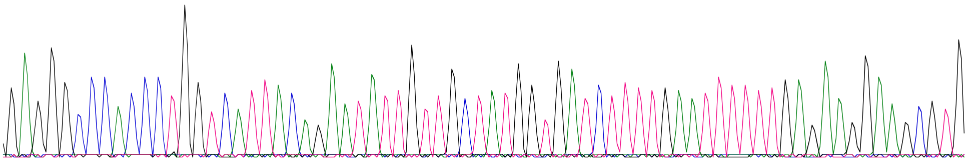

GAGGGCCCACCCTGGTCATTACAGAATATTGTTGCTATGGTGATCTTTTGAATTTTTTGAGAAGGAAGCGTG

3100 3110 3120 3130 3140 3150 3160

ACTCGTTTATTTTCTCAAAGCAAGAAGAGCAGGCAGAAGCGGCACTTTATAAGAACCTTCTGCACTCAACGG

kit.seq(1>5740) → actcgtttattttctcaaagcaagaagagcaggcagaagcggcactttataagaaccttctgcactcaacgg

360-CKIT-SEQF3-A11.ab1(14>947) →

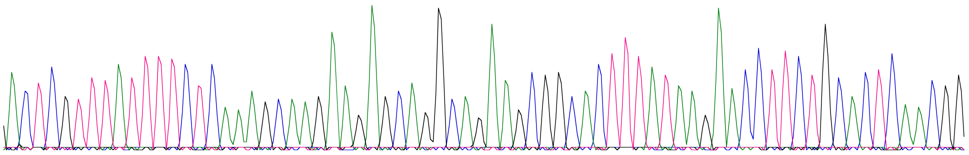

ACTCGTTTATTTTCTCAAAGCAAGAAGAGCAGGCAGAAGCGGCACTTTATAAGAACCTTCTGCACTCAACGG

3170 3180 3190 3200 3210 3220 3230 3240

AGCCTTCCTGTGACAGTTCAAATGAATATATGGACATGAAGCCTGGCGTTTCCTACGTGGTGCCAACCAAGA

kit.seq(1>5740) → agccttcctgtgacagttcaaatgaatatatggacatgaagcctggcgtttcctacgtggtgccaaccaaga

360-CKIT-SEQF3-A11.ab1(14>947) →

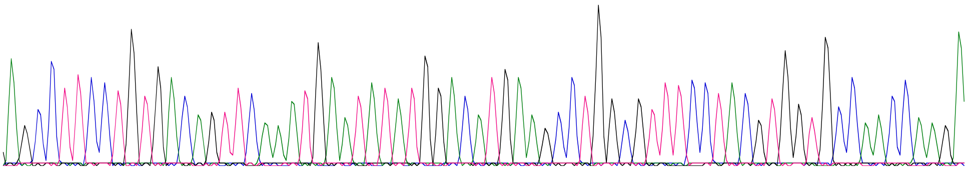

AGCCTTCCTGTGACAGTTCAAATGAATATATGGACATGAAGCCTGGCGTTTCCTACGTGGTGCCAACCAAGA

3250 3260 3270 3280 3290 3300 3310

CAGACAAGAGGAGATCCGCAAGAATAGACTCGTACATAGAAAGAGACGTGACTCCTGCCATCATGGAAGATG

kit.seq(1>5740) → cagacaagaggagatccgcaagaatagactcgtacatagaaagagacgtgactcctgccatcatggaagatg

360-CKIT-SEQF3-A11.ab1(14>947) →

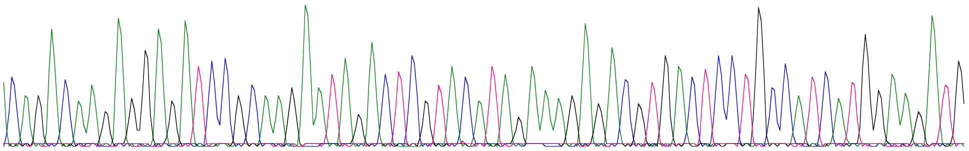

CAGACAAGAGGAGATCCGCAAGAATAGACTCGTACATAGAAAGAGACGTGACTCCTGCCATCATGGAAGATG

3320 3330 3340 3350 3360 3370 3380

ACGAGCTGGCTCTGGACCTGGATGATTTGCTGAGCTTCTCCTACCAGGTGGCCAAGGGCATGGCGTTCCTCG

kit.seq(1>5740) → acgagctggctctggacctggatgatttgctgagcttctcctaccaggtggccaagggcatggcgttcctcg

360-CKIT-SEQF3-A11.ab1(14>947) →

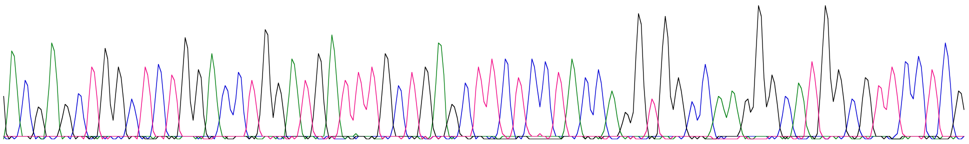

ACGAGCTGGCTCTGGACCTGGATGATTTGCTGAGCTTCTCCTACCAGGTGGCCAAGGGCATGGCGTTCCTCG

3390 3400 3410 3420 3430 3440 3450

CCTCCAAGAATTGTATTCACAGAGATTTGGCAGCCAGGAATATCCTCCTCACTCACGGGCGGATCACAAAGA

kit.seq(1>5740) → cctccaagaattgtattcacagagatttggcagccaggaatatcctcctcactcacgggcggatcacaaaga

360-CKIT-SEQF3-A11.ab1(14>947) →

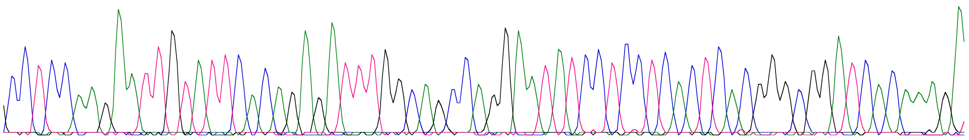

CCTCCAAGAATTGTATTCACAGAGATTTGGCAGCCAGGAATATCCTCCTCACTCACGGGCGGATCACAAAGA

3460 3470 3480 3490 3500 3510 3520

TTTGCGATTTCGGGCTAGCCAGAGACATCAGGAATGATTCGAATTACGTGGTCAAAGGAAATGCACGACTGC

kit.seq(1>5740) → tttgcgatttcgggctagccagagacatcaggaatgattcgaattacgtggtcaaaggaaatgcacgactgc

360-CKIT-SEQF3-A11.ab1(14>947) →

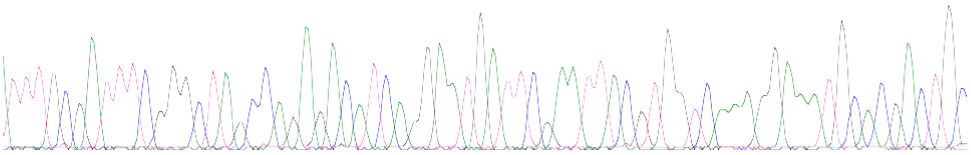

TTTGCGATTTCGGGCTAGCCAGAGACATCAGGAATGATTCGAATTACGTGGTCAAAGGAAATGCACGACTGC

360-CKIT-SEQF4-A12.ab1(23>769) →

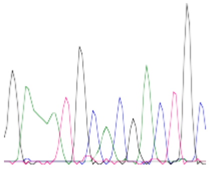

GAAATGCACGACTGC

3530 3540 3550 3560 3570 3580 3590 3600

CCGTGAAGTGGATGGCACCAGAGAGCATTTTCAGCTGCGTGTACACATTTGAAAGTGATGTCTGGTCCTATG

kit.seq(1>5740) → ccgtgaagtggatggcaccagagagcattttcagctgcgtgtacacatttgaaagtgatgtctggtcctatg

360-CKIT-SEQF3-A11.ab1(14>947) →

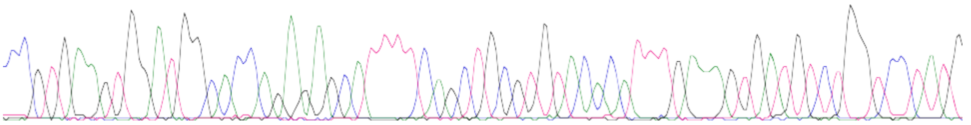

CCGTGAAGTGGATGGCACCAGAGAGCATTTTCAGCTGCGTGTACACATTTGAAAGTGATGTCTGGTCCTATG

360-CKIT-SEQF4-A12.ab1(23>769) →

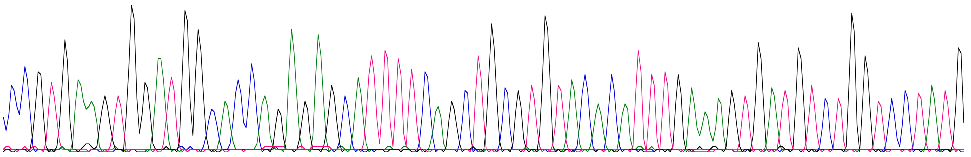

CCGTGAAGTGGATGGCACCAGAGAGCATTTTCAGCTGCGTGTACACATTTGAAAGTGATGTCTGGTCCTATG

3610 3620 3630 3640 3650 3660 3670

GGATTTTCCTCTGGGAGCTCTTCTCCTTAGGAAGCAGCCCCTACCCAGGGATGCCGGTCGACTCCAAGTTCT

kit.seq(1>5740) → ggattttcctctgggagctcttctccttaggaagcagcccctacccagggatgccggtcgactccaagttct

360-CKIT-SEQF3-A11.ab1(14>947) →

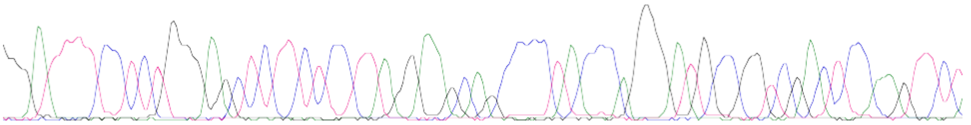

GGATTTTCCTCTGGGAGCTCTTCTCCTTAgGAAGCAGCCCCTACCCAGGGATGCCGGTCGACTCCAAGTTCT

360-CKIT-SEQF4-A12.ab1(23>769) →

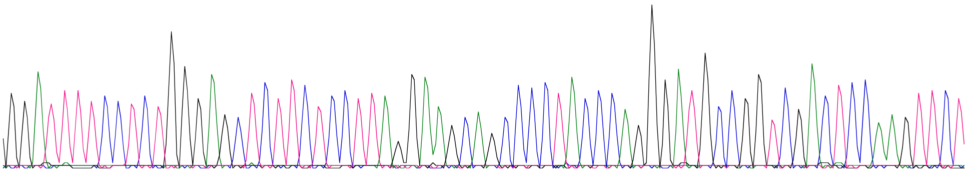

GGATTTTCCTCTGGGAGCTCTTCTCCTTAGGAAGCAGCCCCTACCCAGGGATGCCGGTCGACTCCAAGTTCT

3680 3690 3700 3710 3720 3730 3740

ACAAGATGATCAAGGAAGGCTTCCGGATGGTCAGCCCGGAGCACGCGCCTGCCGAAATGTATGACGTCATGA

kit.seq(1>5740) → acaagatgatcaaggaaggcttccggatggtcagcccggagcacgcgcctgccgaaatgtatgacgtcatga

360-CKIT-SEQF3-A11.ab1(14>947) →

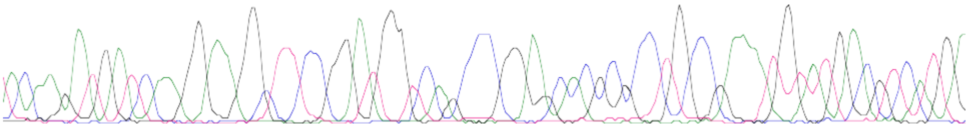

ACAAGATGATCAAgGAAgGCTTCCGGATGGTCAGCCCGGAGCACGCGCCTGCCGAAATGTATGACGTCATGA

360-CKIT-SEQF4-A12.ab1(23>769) →

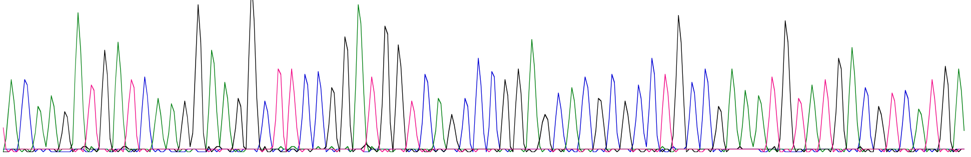

ACAAGATGATCAAGGAAGGCTTCCGGATGGTCAGCCCGGAGCACGCGCCTGCCGAAATGTATGACGTCATGA

3750 3760 3770 3780 3790 3800 3810

AGACTTGCTGGGACGCTGACCCCTTGAAAAGGCCAACATTCAAGCAGGTTGTCCAACTTATTGAGAAGCAGA

kit.seq(1>5740) → agacttgctgggacgctgaccccttgaaaaggccaacattcaagcaggttgtccaacttattgagaagcaga

360-CKIT-SEQF3-A11.ab1(14>947) →

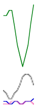

AGA

360-CKIT-SEQF4-A12.ab1(23>769) →

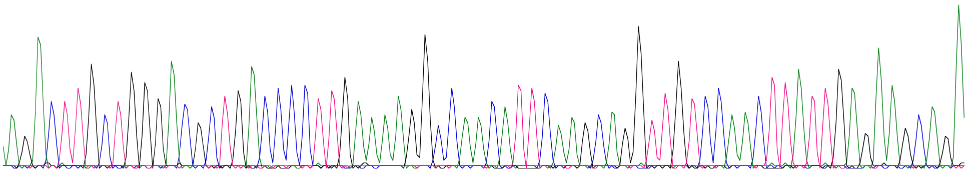

AGACTTGCTGGGACGCTGACCCCTTGAAAAGGCCAACATTCAAGCAGGTTGTCCAACTTATTGAGAAGCAGA

3820 3830 3840 3850 3860 3870 3880

TCTCGGACAGCACCAAGCACATTTACTCCAACTTGGCAAACTGCAACCCCAACCCAGAGAACCCCGTGGTGG

kit.seq(1>5740) → tctcggacagcaccaagcacatttactccaacttggcaaactgcaaccccaacccagagaaccccgtggtgg

360-CKIT-SEQF4-A12.ab1(23>769) →

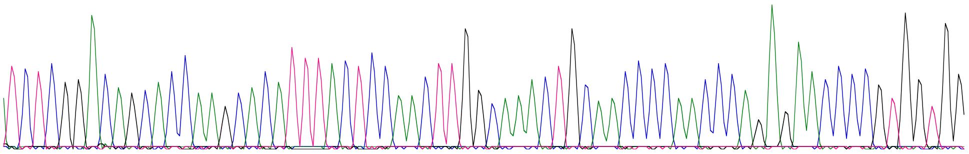

TCTCGGACAGCACCAAGCACATTTACTCCAACTTGGCAAACTGCAACCCCAACCCAGAGAACCCCGTGGTGG

3890 3900 3910 3920 3930 3940 3950 3960

TGGACCATTCCGTGAGGGTCAACTCGGTGGGCAGCAGCGCCTCTTCTACGCAGCCATTGCTCGTGCACGAAG

kit.seq(1>5740) → tggaccattccgtgagggtcaactcggtgggcagcagcgcctcttctacgcagccattgctcgtgcacgaag

360-CKIT-SEQF4-A12.ab1(23>769) →

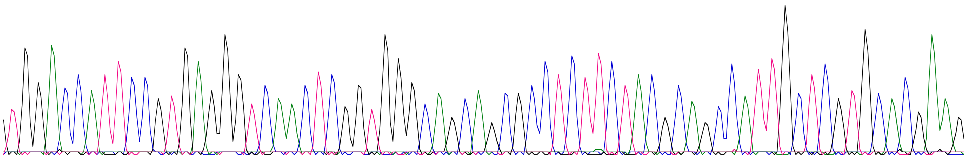

TGGACCATTCCGTGAGGGTCAACTCGGTGGGCAGCAGCGCCTCTTCTACGCAGCCATTGCTCGTGCACGAAG

3970 3980 3990 4000 4010 4020 4030

ATGCCGCCACGAACTTCTCTCTGTTAAAGCAAGCAGGAGACGTGGAAGAAAACCCCGGTCCTATGGTGAGCA

kit.seq(1>5740) → atgccgccacgaacttctctctgttaaagcaagcaggagacgtggaagaaaaccccggtcctatggtgagca

360-CKIT-SEQF4-A12.ab1(23>769) →

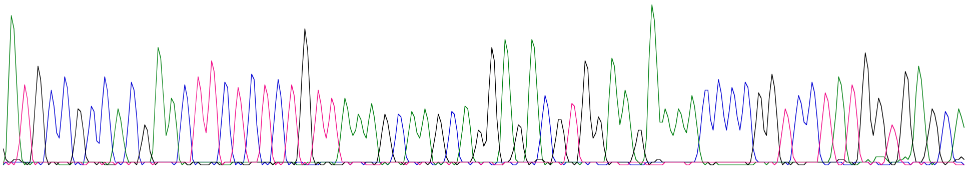

ATGCCGCCACGAACTTCTCTCTGTTAAAGCAAGCAGGAGACGTGGAAGAAAACCCCGGTCCTATGGTGAGCA

4040 4050 4060 4070 4080 4090 4100

AGGGCGAGGAGCTGTTCACCGGGGTGGTGCCCATCCTGGTCGAGCTGGACGGCGACGTAAACGGCCACAAGT

kit.seq(1>5740) → agggcgaggagctgttcaccggggtggtgcccatcctggtcgagctggacggcgacgtaaacggccacaagt

360-CKIT-SEQF4-A12.ab1(23>769) →

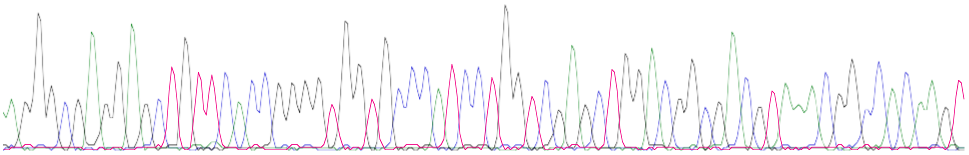

AGGGCGAGGAGCTGTTCACCGGGGTGGTGCCCATCCTGGTCGAGCTGGACGGCGACGTAAACGGCCACAAGT

3-360-CKIT-K1-TR2-D01.ab1(1>712) ←

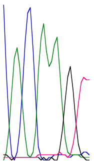

CACAAGT

4110 4120 4130 4140 4150 4160 4170

TCAGCGTGTCCGGCGAGGGCGAGGGCGATGCCACCTACGGCAAGCTGACCCTGAAGTTCATCTGCACCACCG

kit.seq(1>5740) → tcagcgtgtccggcgagggcgagggcgatgccacctacggcaagctgaccctgaagttcatctgcaccaccg

360-CKIT-SEQF4-A12.ab1(23>769) →

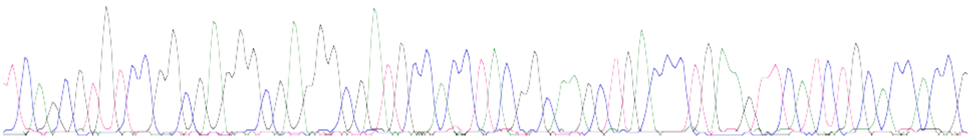

TCAGCGTGTCCGGCGAGGGCGAGGGCGATGCCACCTACGGCAAGCTGACCCTGAAGTTCATCTGCACCACCG

3-360-CKIT-K1-TR2-D01.ab1(1>712) ←

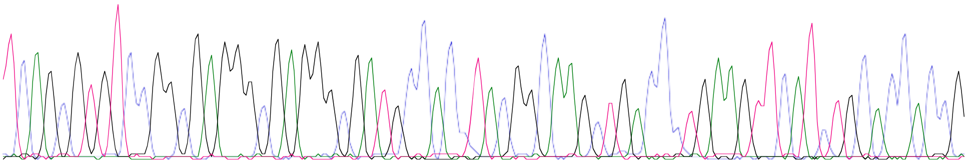

TCAGCGTGTCCGGCGAGGGCGAGGGCGATGCCACCTACGGCAAGCTGACCCTGAAGTTCATCTGCACCACCG

4180 4190 4200 4210 4220 4230 4240

GCAAGCTGCCCGTGCCCTGGCCCACCCTCGTGACCACCCTGACCTACGGCGTGCAGTGCTTCAGCCGCTACC

kit.seq(1>5740) → gcaagctgcccgtgccctggcccaccctcgtgaccaccctgacctacggcgtgcagtgcttcagccgctacc

360-CKIT-SEQF4-A12.ab1(23>769) →

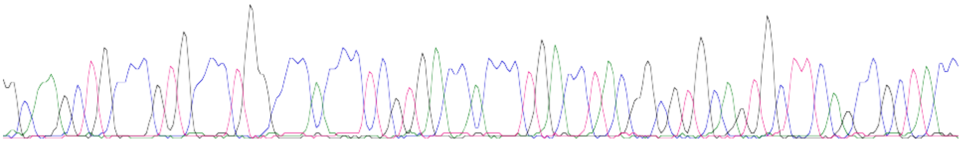

GCAAGCTGCCCGTGCCCTGGCCCACCCTCGTGACCACCCTGACCTACGGCGTGCAGTGCTTCAGCCGCTACC

3-360-CKIT-K1-TR2-D01.ab1(1>712) ←

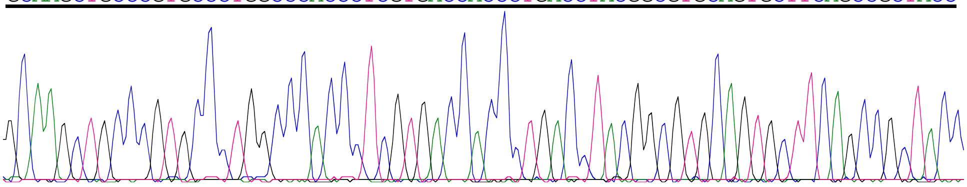

GCAAGCTGCCCGTGCCCTGGCCCACCCTCGTGACCACCCTGACCTACGGCGTGCAGTGCTTCAGCCGCTACC

4250 4260 4270 4280 4290 4300 4310 4320

CCGACCACATGAAGCAGCACGACTTCTTCAAGTCCGCCATGCCCGAAGGCTACGTCCAGGAGCGCACCATCT

kit.seq(1>5740) → ccgaccacatgaagcagcacgacttcttcaagtccgccatgcccgaaggctacgtccaggagcgcaccatct

360-CKIT-SEQF4-A12.ab1(23>769) →

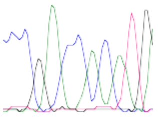

CCGACCACATGA

3-360-CKIT-K1-TR2-D01.ab1(1>712) ←

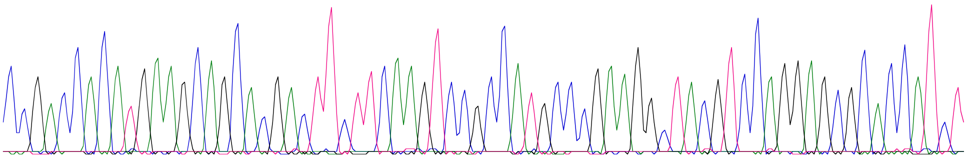

CCGACCACATGAAGCAGCACGACTTCTTCAAGTCCGCCATGCCCGAAGGCTACGTCCAGGAGCGCACCATCT

4330 4340 4350 4360 4370 4380 4390

TCTTCAAGGACGACGGCAACTACAAGACCCGCGCCGAGGTGAAGTTCGAGGGCGACACCCTGGTGAACCGCA

kit.seq(1>5740) → tcttcaaggacgacggcaactacaagacccgcgccgaggtgaagttcgagggcgacaccctggtgaaccgca

3-360-CKIT-K1-TR2-D01.ab1(1>712) ←

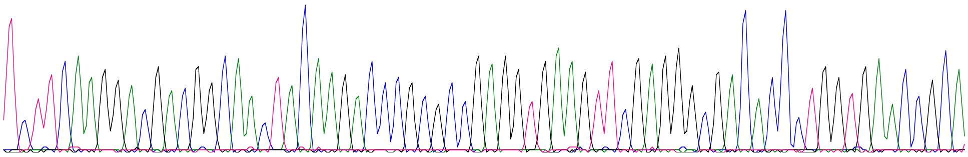

TCTTCAAGGACGACGGCAACTACAAGACCCGCGCCGAGGTGAAGTTCGAGGGCGACACCCTGGTGAACCGCA

4400 4410 4420 4430 4440 4450 4460

TCGAGCTGAAGGGCATCGACTTCAAGGAGGACGGCAACATCCTGGGGCACAAGCTGGAGTACAACTACAACA

kit.seq(1>5740)
3-360-CKIT-K1-TR2-D01.ab1(1>712)

tcgagctgaagggcatcgacttcaaggaggacggcaacatcctggggcacaagctggagtacaactacaaca

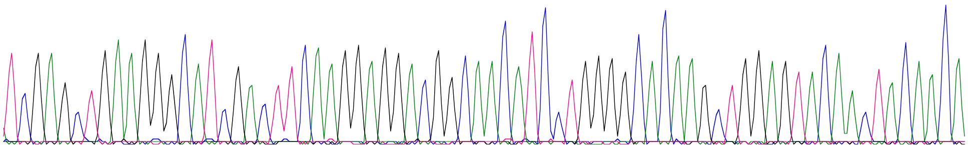

TCGAGCTGAAGGGCATCGACTTCAAGGAGGACGGCAACATCCTGGGGCACAAGCTGGAGTACAACTACAACA

4470 4480 4490 4500 4510 4520 4530

GCCACAACGTCTATATCATGGCCGACAAGCAGAAGAACGGCATCAAGGTGAACTTCAAGATCCGCCACAACA

kit.seq(1>5740)
3-360-CKIT-K1-TR2-D01.ab1(1>712)

gccacaacgtctatatcatggccgacaagcagaagaacggcatcaaggtgaacttcaagatccgccacaaca

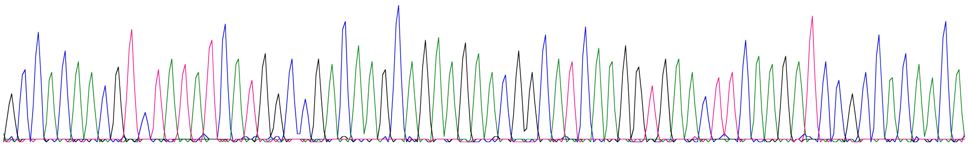

GCCACAACGTCTATATCATGGCCGACAAGCAGAAGAACGGCATCAAGGTGAACTTCAAGATCCGCCACAACA

4540 4550 4560 4570 4580 4590 4600

TCGAGGACGGCAGCGTGCAGCTCGCCGACCACTACCAGCAGAACACCCCCATCGGCGACGGCCCCGTGCTGC

kit.seq(1>5740)
3-360-CKIT-K1-TR2-D01.ab1(1>712)

tcgaggacggcagcgtgcagctcgccgaccactaccagcagaacacccccatcggcgacggccccgtgctgc

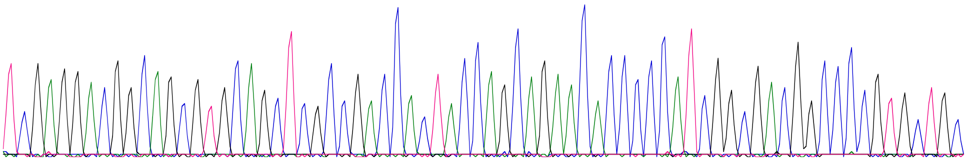

TCGAGGACGGCAGCGTGCAGCTCGCCGACCACTACCAGCAGAACACCCCCATCGGCGACGGCCCCGTGCTGC

4610 4620 4630 4640 4650 4660 4670 4680

TGCCCGACAACCACTACCTGAGCACCCAGTCCGCCCTGAGCAAAGACCCCAACGAGAAGCGCGATCACATGG

kit.seq(1>5740)
3-360-CKIT-K1-TR2-D01.ab1(1>712)

tgcccgacaaccactacctgagcacccagtccgccctgagcaaagaccccaacgagaagcgcgatcacatgg

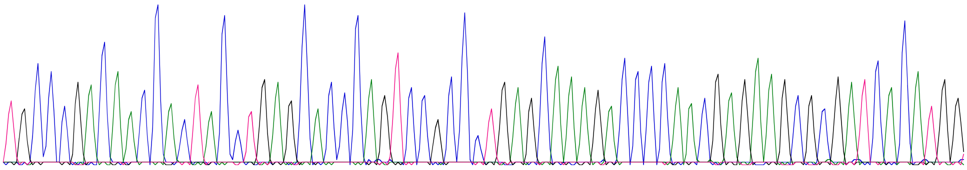

TGCCCGACAACCACTACCTGAGCACCCAGTCCGCCCTGAGCAAAGACCCCAACGAGAAGCGCGATCACATGG

4690 4700 4710 4720 4730 4740 4750

TCCTGCTGGAGTTCGTGACCGCCGCCGGGATCACTCTCGGCATGGACGAGCTGTACAAGTGAGCAGAAACCC

kit.seq(1>5740)
3-360-CKIT-K1-TR2-D01.ab1(1>712)

tcctgctggagttcgtgaccgccgccgggatcactctcggcatggacgagctgtacaagtgagcagaaaccc

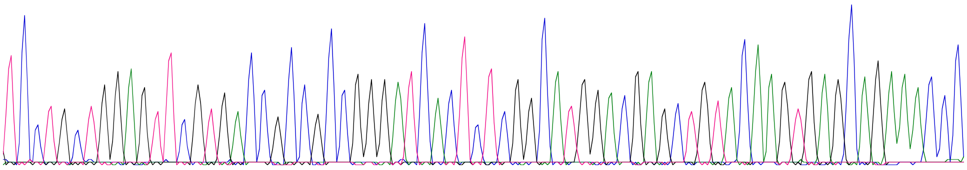

TCCTGCTGGAGTTCGTGACCGCCGCCGGGATCACTCTCGGCATGGACGAGCTGTACAAGTGAGCAGAAACCC

4760 4770 4780 4790 4800 4810 4820

AAGTCCAACAGGCTTTGCTGCTGTCTCCGACCCCGTCCTTCTGGCTTCTGTGATGGTTACTTGGTTTCCCTT

kit.seq(1>5740)
3-360-CKIT-K1-TR2-D01.ab1(1>712)

aagtccaacaggctttgctgctgtctccgaccccgtccttctggcttctgtgatggttacttggtttccctt

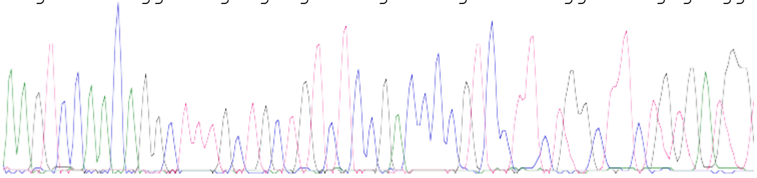

AAGTCCAACAGGCTTTGCTGCTGTCTCCGACCCCGTCCTTCTGGCTTCTGTGATGGT

4760 4770 4780 4790 4800 4810 4820

AAGTCCAACAGGCTTTGCTGCTGTCTCCGACCCCGTCCTTCTGGCTTCTGTGATGGTTACTTGGTTTCCCTT

3-360-CKIT-SEQF7-D02.ab1(12>935) →

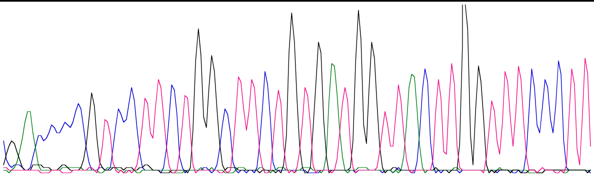

GACCCCGTCCTTCTGGCTTCTGTGATGGTTACTTGGTTTCCCTT

4830 4840 4850 4860 4870 4880 4890

TGACTTGCATCCTATTCCAGGGTAGCGAGTTCCCCACCCCACCTCCAACCCCACTGTGATTCCGCCTTTACG

kit.seq(1>5740) →
3-360-CKIT-SEQF7-D02.ab1(12>935) →

tgacttgcatcctattccagggtagcgagttccccaccccacctccaaccccactgtgattccgcctttacg

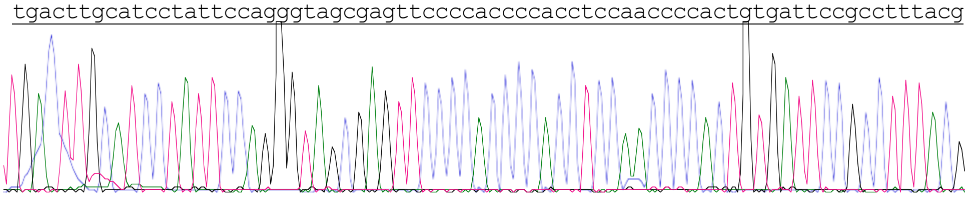

TGACTTGCATCCTATTCCAGGGTAGCGAGTTCCCCACCCCACCTCCAACCCCACTGTGATTCCGCCTTTACG

4900 4910 4920 4930 4940 4950 4960

AGCACACACTTTAGTGGCCGATGGCCTTTTCTTTTCTGCCATCAGCCACCGTCCCGCTGCGAAGGTCCGAAC

kit.seq(1>5740) →
3-360-CKIT-SEQF7-D02.ab1(12>935) →

agcacacactttagtggccgatggccttttcttttctgccatcagccaccgtcccgctgcgaaggtccgaac

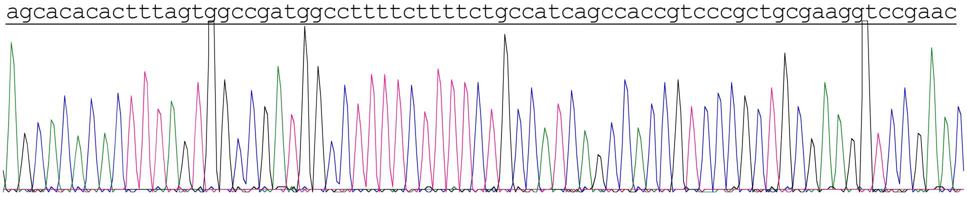

AGCACACACTTTAGTGGCCGATGGCCTTTTCTTTTCTGCCATCAGCCACCGTCCCGCTGCGAAGGTCCGAAC

3-360-CKIT-D-3R1-D03.ab1(1>784) ←

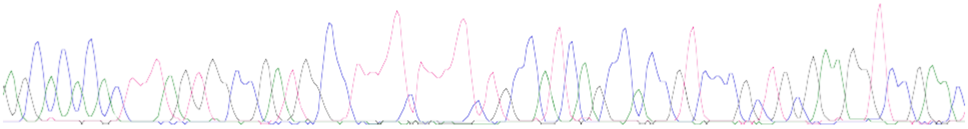

AGCACACACTTTAGTGGCCGATGGCCTTTTCTTTTCTGCCATCAGCCACCGTCCCGCTGCGAAGGTCCGAAC

4970 4980 4990 5000 5010 5020 5030 5040

TGTATGTATATATTTTCCCAATAGCAAAGTAGCTCCTACTGTAAACAGAAGGACTCCTCCTGCTTTAGAGGA

kit.seq(1>5740) →
3-360-CKIT-SEQF7-D02.ab1(12>935) →

tgtatgtatatattttcccaatagcaaagtagctcctactgtaaacagaaggactcctcctgctttagagga

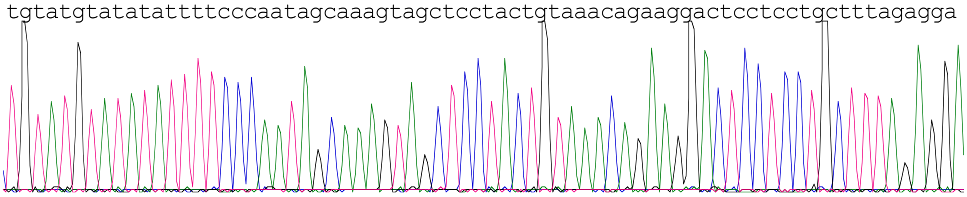

TGTATGTATATATTTTCCCAATAGCAAAGTAGCTCCTACTGTAAACAGAAGGACTCCTCCTGCTTTAGAGGA

3-360-CKIT-D-3R1-D03.ab1(1>784) ←

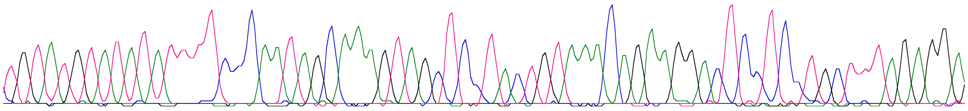

TGTATGTATATATTTTCCCAATAGCAAAGTAGCTCCTACTGTAAACAGAAGGACTCCTCCTGCTTTAGAGGA

5050 5060 5070 5080 5090 5100 5110

GAAGGGAAGGGCGGGGTGAAACTGGATGCCCAGAGTTCTTCCCCCAGTGCTCCCCTGAGTGTATTTGAAAAG

kit.seq(1>5740) →
3-360-CKIT-SEQF7-D02.ab1(12>935) →

gaagggaagggcggggtgaaactggatgcccagagttcttcccccagtgctcccctgagtgtatttgaaaag

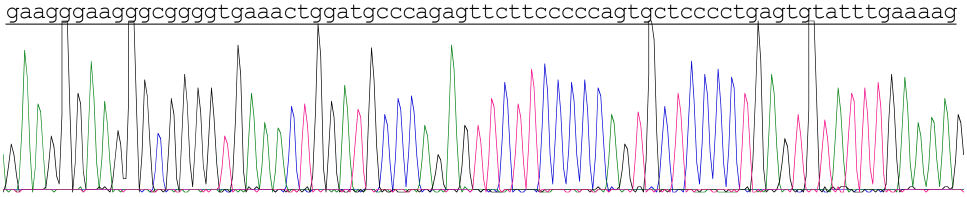

GAAGGGAAGGGCGGGGTGAAACTGGATGCCCAGAGTTCTTCCCCCAGTGCTCCCCTGAGTGTATTTGAAAAG

5050 5060 5070 5080 5090 5100 5110

GAAGGGAAGGGCGGGGTGAAACTGGATGCCCAGAGTTCTTCCCCCAGTGCTCCCCTGAGTGTATTTGAAAAG

3-360-CKIT-D-3R1-D03.ab1(1>784) ←

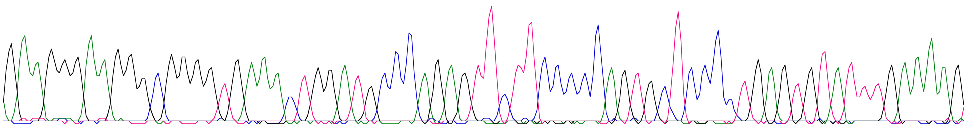

GAAGGGAAGGGCGGGGTGAAACTGGATGCCCAGAGTTCTTCCCCCAGTGCTCCCCTGAGTGTATTTGAAAAG

5120 5130 5140 5150 5160 5170 5180

TATGGCCAGTAGTTCACTTGAAGAATAGATGTAGTCCCATTTGGCCCTGAGAGCCATCCTTAATGATGGGAG

kit.seq(1>5740) → tatggccagtagttcacttgaagaatagatgtagtcccatttggccctgagagccatccttaatgatgggag
3-360-CKIT-SEQF7-D02.ab1(12>935) →

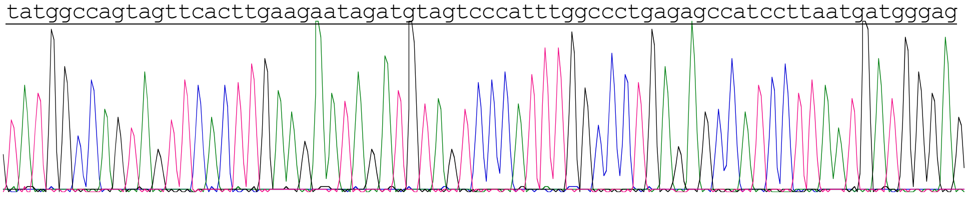

TATGGCCAGTAGTTCACTTGAAGAATAGATGTAGTCCCATTTGGCCCTGAGAGCCATCCTTAATGATGGGAG

3-360-CKIT-D-3R1-D03.ab1(1>784) ←

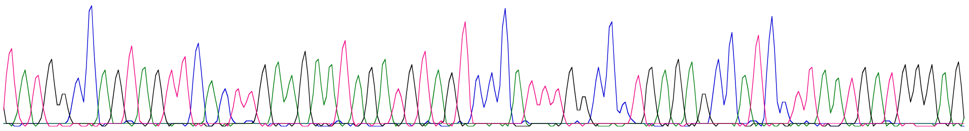

TATGGCCAGTAGTTCACTTGAAGAATAGATGTAGTCCCATTTGGCCCTGAGAGCCATCCTTAATGATGGGAG

5190 5200 5210 5220 5230 5240 5250

ATATATGTAGCAAGACTAGAAAAGGAAAGCCAAGCCCTTTGTGTAGAAAGCAGACCATTCTTAGAACAGAGG

kit.seq(1>5740) → atatatgtagcaagactagaaaaggaaagccaagccctttgtgtagaaagcagaccattcttagaacagagg
3-360-CKIT-SEQF7-D02.ab1(12>935) →

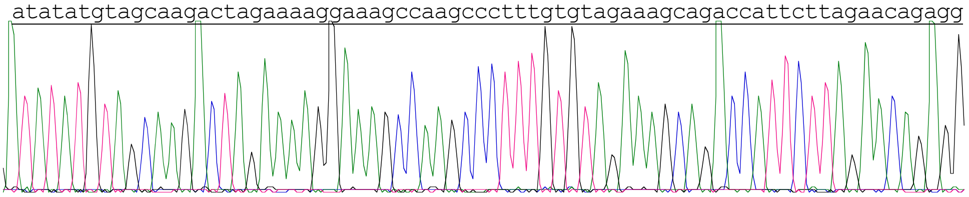

ATATATGTAGCAAGACTAGAAAAGGAAAGCCAAGCCCTTTGTGTAGAAAGCAGACCATTCTTAGAACAGAGG

3-360-CKIT-D-3R1-D03.ab1(1>784) ←

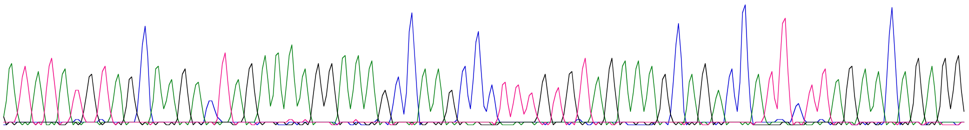

ATATATGTAGCAAGACTAGAAAAGGAAAGCCAAGCCCTTTGTGTAGAAAGCAGACCATTCTTAGAACAGAGG

5260 5270 5280 5290 5300 5310 5320

GCAACGGGGCATCGGAAGTCTGGTCACGCTAAGAAGACCGAGGCTGAGAAGGAACAAGCCAGGGGAAGCGTG

kit.seq(1>5740) → gcaacggggcatcggaagtctggtcacgctaagaagaccgaggctgagaaggaacaagccaggggaagcgtg
3-360-CKIT-SEQF7-D02.ab1(12>935) →

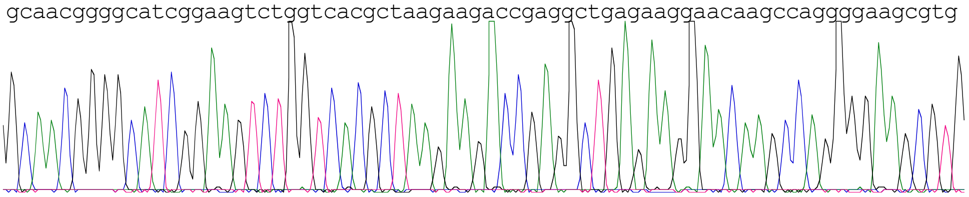

GCAACGGGGCATCGGAAGTCTGGTCACGCTAAGAAGACCGAGGCTGAGAAGGAACAAGCCAGGGGAAGCGTG

3-360-CKIT-D-3R1-D03.ab1(1>784) ←

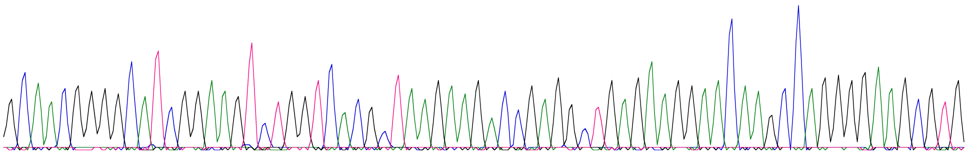

GCAACGGGGCATCGGAAGTCTGGTCACGCTAAGAAGACCGAGGCTGAGAAGGAACAAGCCAGGGGAAGCGTG

5330 5340 5350 5360 5370 5380 5390 5400

AACAATGATGCTCTGCTCTGGGCTGCCGCTCGGGCTTCTGTACAACTGACCTGGTTTCTCAGTACTTTGCTG

kit.seq(1>5740) → aacaatgatgctctgctctgggctgccgctcgggcttctgtacaactgacctggtttctcagtactttgctg

3-360-CKIT-SEQF7-D02.ab1(12>935) →

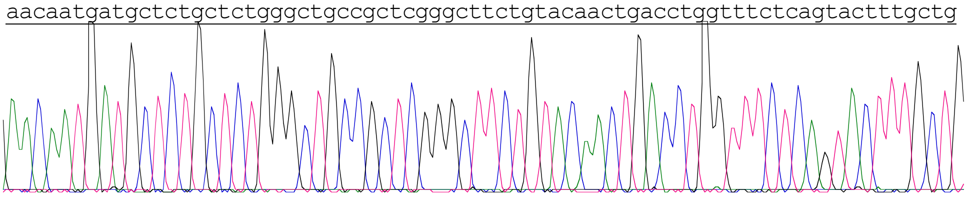

AACAATGATGCTCTGCTCTGGGCTGCCGCTCGGGCTTCTGTACAACTGACCTGGTTTCTCAGTACTTTGCTG

3-360-CKIT-D-3R1-D03.ab1(1>784) ←

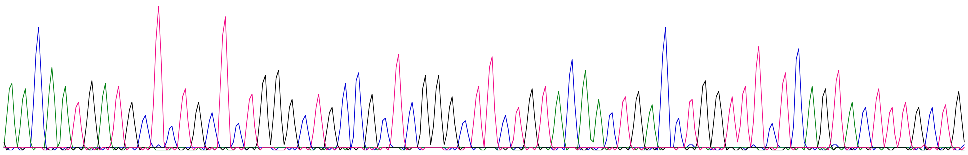

AACAATGATGCTCTGCTCTGGGCTGCCGCTCGGGCTTCTGTACAACTGACCTGGTTTCTCAGTACTTTGCTG

5410 5420 5430 5440 5450 5460 5470

TCTGGGAGTAGCATTGGAATCAAGGCCTCCTCCCTAGTCAGCCTTTGTATATACTCATCTATACGTTGTATG

kit.seq(1>5740) → tctgggagtagcattggaatcaaggcctcctccctagtcagcctttgtatatactcatctatacgttgtatg

3-360-CKIT-SEQF7-D02.ab1(12>935) →

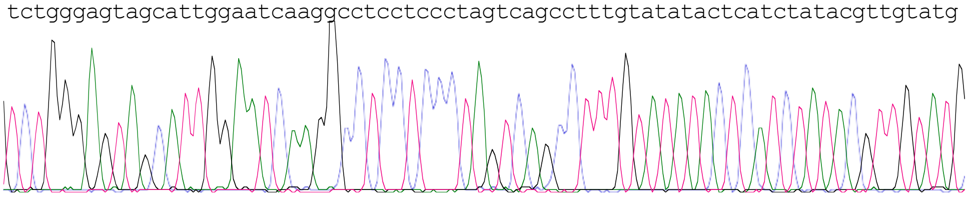

TCTGGGAGTAGCATTGGAATCAAGGCCTCCTCCCTAGTCAGCCTTTGTATATACTCATCTATACGTTGTATG

3-360-CKIT-D-3R1-D03.ab1(1>784) ←

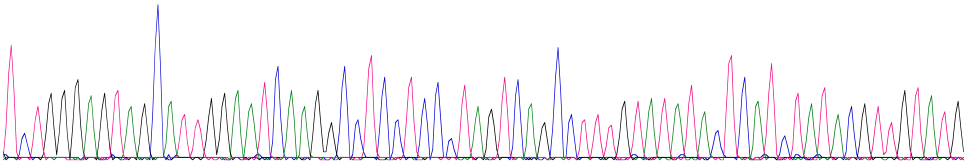

TCTGGGAGTAGCATTGGAATCAAGGCCTCCTCCCTAGTCAGCCTTTGTATATACTCATCTATACGTTGTATG

5480 5490 5500 5510 5520 5530 5540

CGTTCATACTTTGGAGGAGGGATTTCCCACAAGCTTTCGTTTCTGTGTACAGCCCTGGCATTAGACCTACTG

kit.seq(1>5740) → cgttcatactttggaggagggatttcccacaagctttcgtttctgtgtacagccctggcattagacctactg

3-360-CKIT-SEQF7-D02.ab1(12>935) →

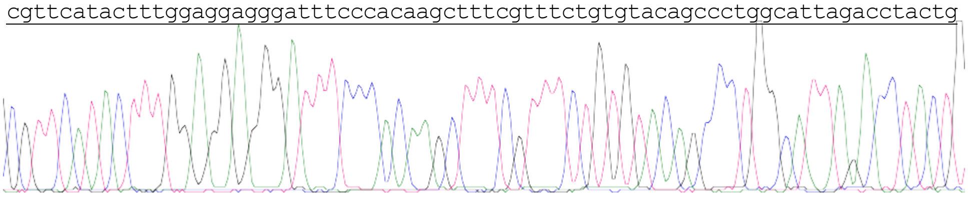

CGTTCATACTTTGGAGGAGGGATTTCCCACAAGCTTTCGTTTCTGTGTACAGCCCTGGCATTAGACCTACTG

3-360-CKIT-D-3R1-D03.ab1(1>784) ←

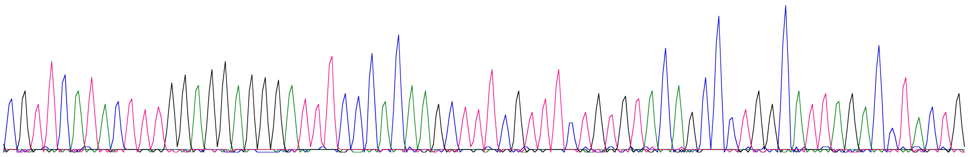

CGTTCATACTTTGGAGGAGGGATTTCCCACAAGCTTTCGTTTCTGTGTACAGCCCTGGCATTAGACCTACTG

5550 5560 5570 5580 5590 5600 5610

TGTGTAAGAATAGATTAAGAGCCATACATATTTGAAGGAAACAGTTAAATGTTTTTTGGTTGTGGTTGTTGT

kit.seq(1>5740) → tgtgtaagaatagattaagagccatacatatttgaaggaaacagttaaatgttttttggttgtggttgttgt

3-360-CKIT-SEQF7-D02.ab1(12>935) →

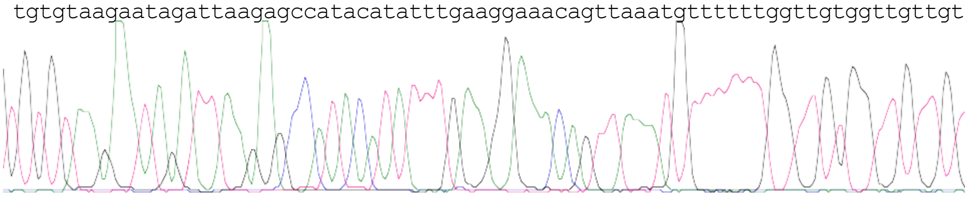

TGTGTAAGAATAGATTAAGAGCCATACATATTTGAAGGAAACAGTTAAATGTTTTTTGGTTGTGGTTGTTGT

3-360-CKIT-D-3R1-D03.ab1(1>784) ←

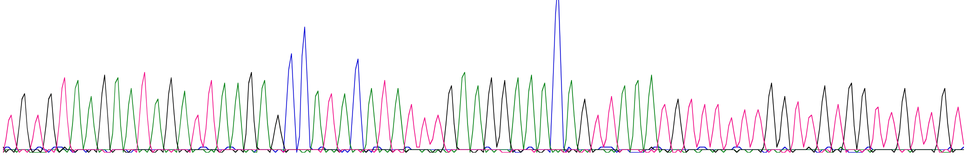

TGTGTAAGAATAGATTAAGAGCCATACATATTTGAAGGAAACAGTTAAATGTTTTTTGGTTGTGGTTGTTGT

5620 5630 5640 5650 5660 5670 5680

TGTTGTTGTTTTAAAGAAAAAAATGTATATGCTAAGCACAATCTTTATAAGACCTCTTAGCCAACATACTTG

kit.seq(1>5740) tgttgttgttttaaagaaaaaaatgtatatgctaagcacaatctttataagacctcttagccaacatacttg

3-360-CKIT-SEQF7-D02.ab1(12>935)

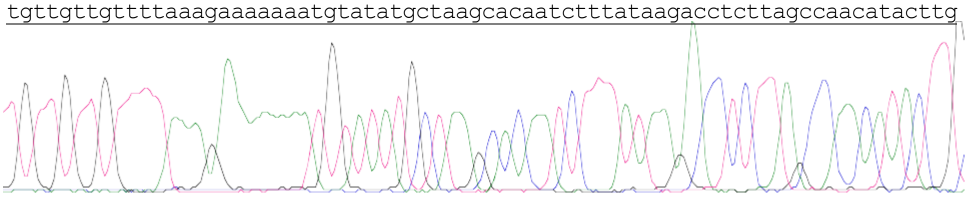

TGTTGTTGTTTTAAAGAAAAAAATGTATATGCTAAGCACAATCTTTATAAGACCTCTTAGCCAACATACTTG

3-360-CKIT-D-3R1-D03.ab1(1>784)

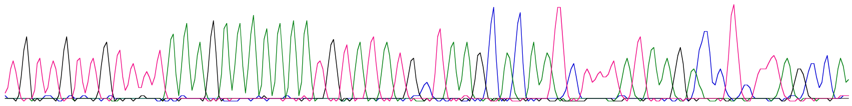

TGTTGTTGTTTTAAAGAAAAAAATGTATATGCTAAGCACAATCTTTATAAGACCTCTTAGCCAA

5690 5700 5710 5720 5730 5740

CTCTGTCTACACTTCGgaacaagccttccatgtcagagtggctttgcaggca

kit.seq(1>5740) ctctgtctacacttcggaacaagccttccatgtcagagtggctttgcaggca

3-360-CKIT-SEQF7-D02.ab1(12>935)

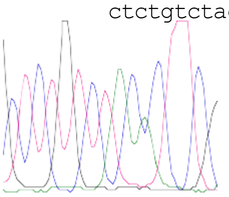

CTCTGTCTACACTTCG
