# Supplementary figures and images for: Functional Analysis of KIT Gene Structural Mutations Causing the Porcine Dominant White Phenotype Using Genome Edited Mouse Models
Source: Front Genet. 2020 Mar 3;11:138. doi: 10.3389/fgene.2020.00138 (PMC7063667; doi:10.3389/fgene.2020.00138)

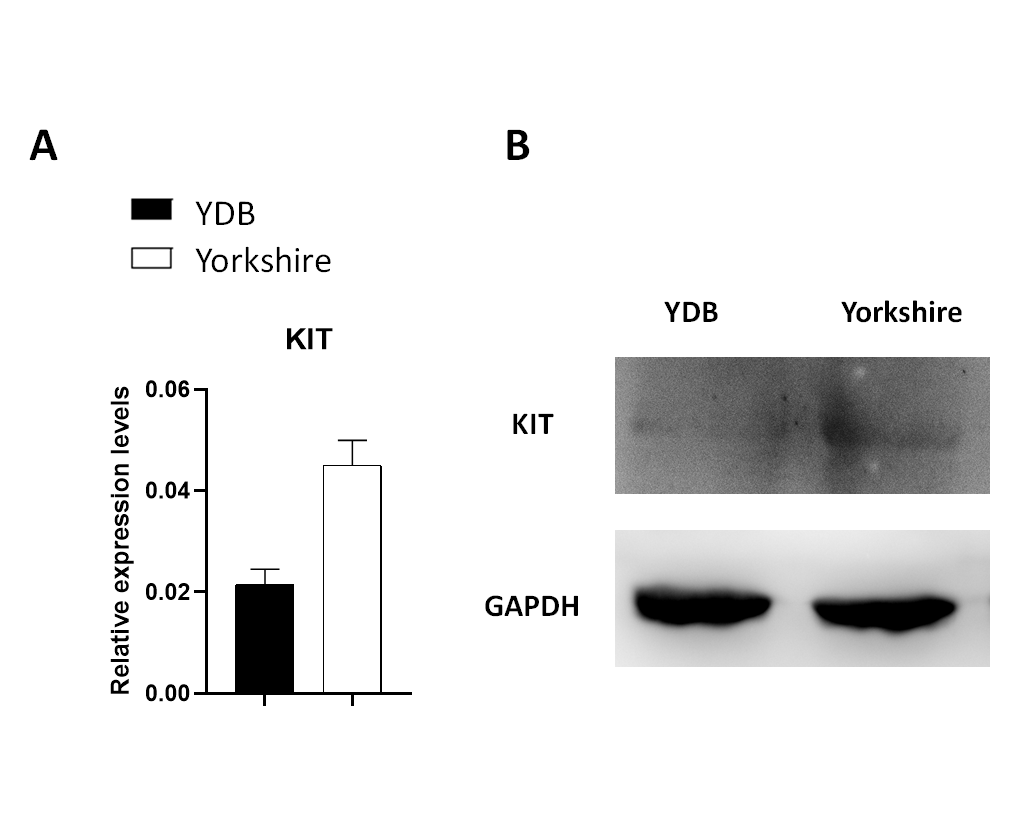

Supplement: Supplementary file 2 [file Image_1.tif]

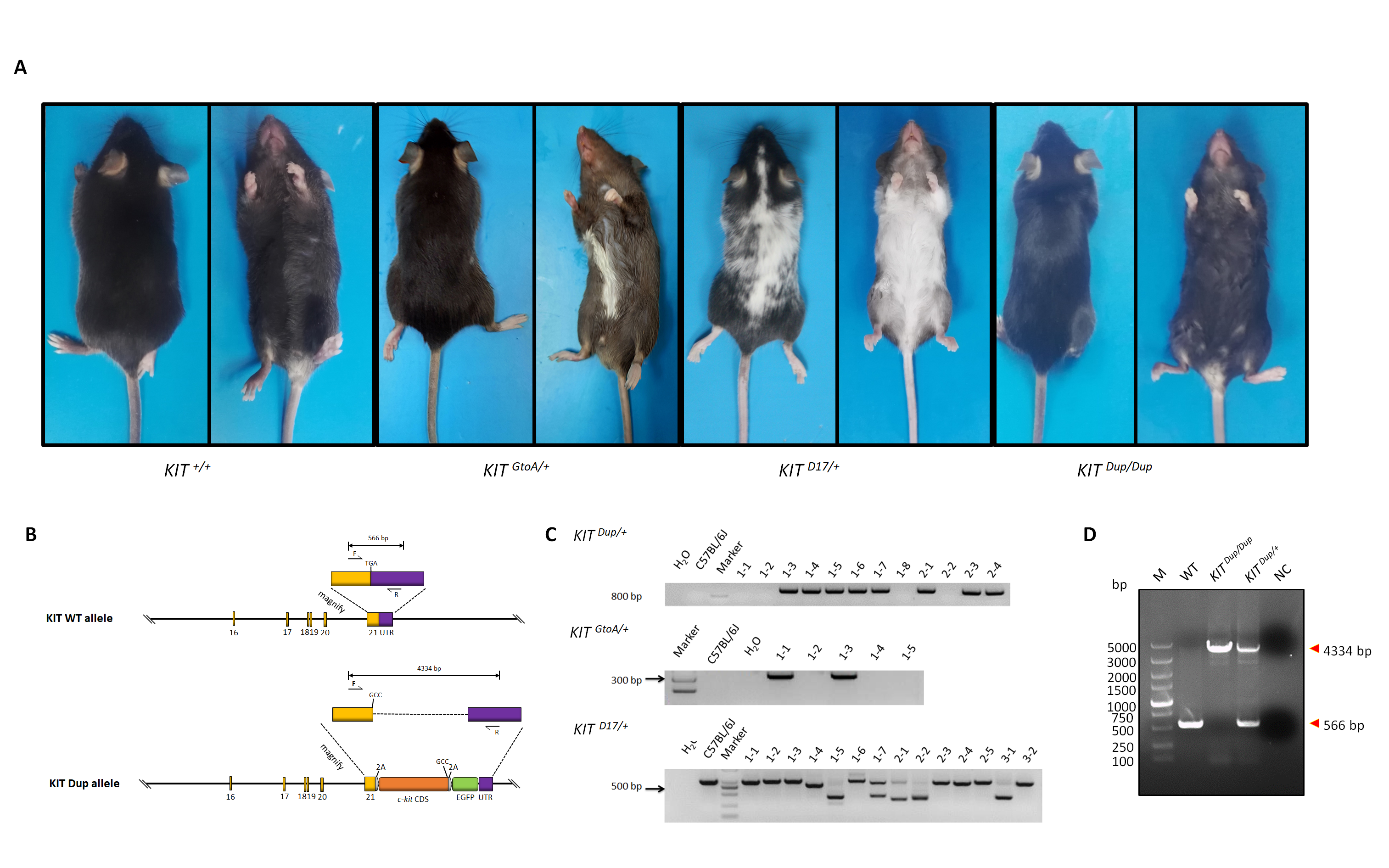

Supplement: Supplementary file 3 [file Image_2.tif]

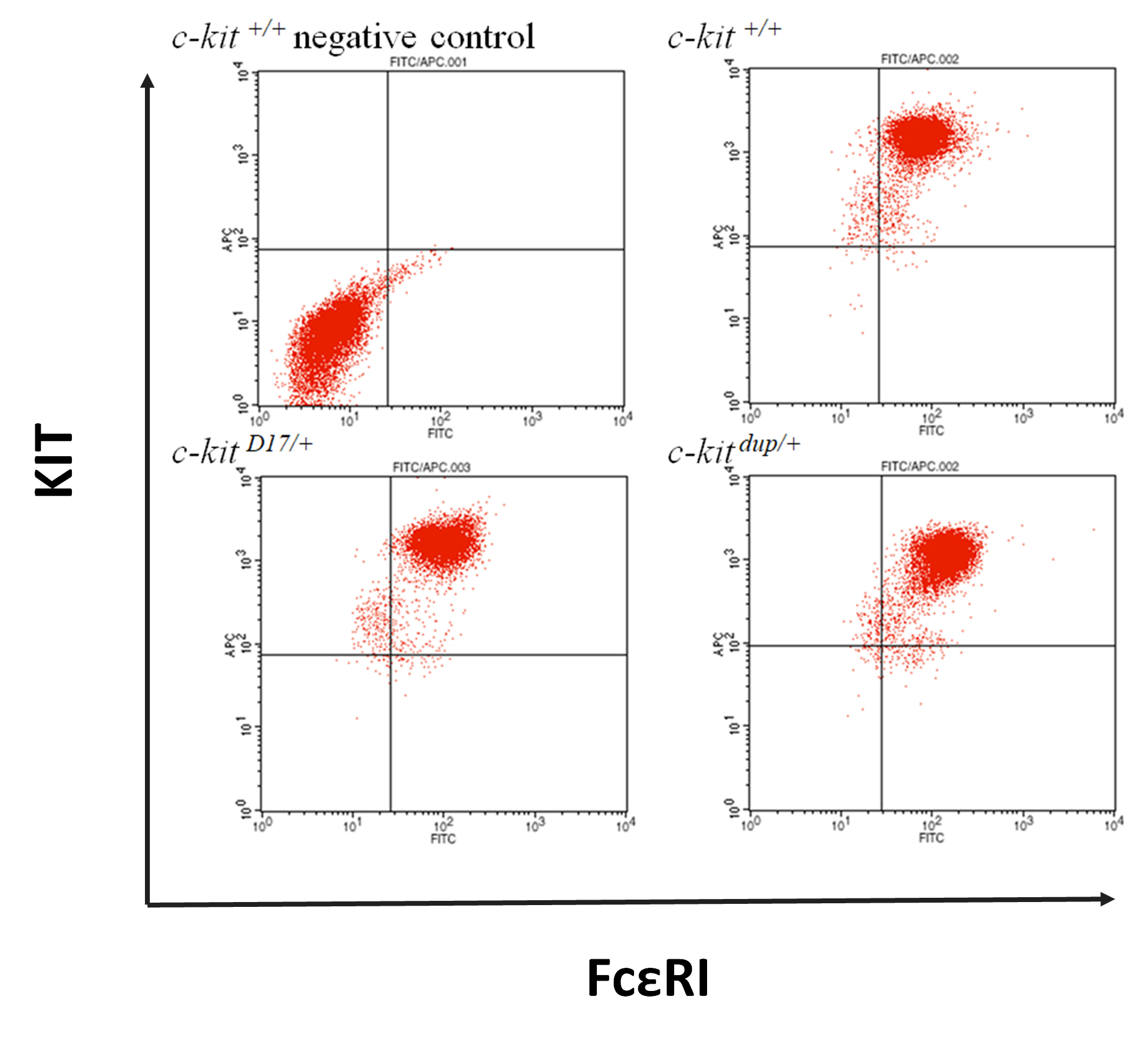

Supplement: Supplementary file 4 [file Image_3.tif]

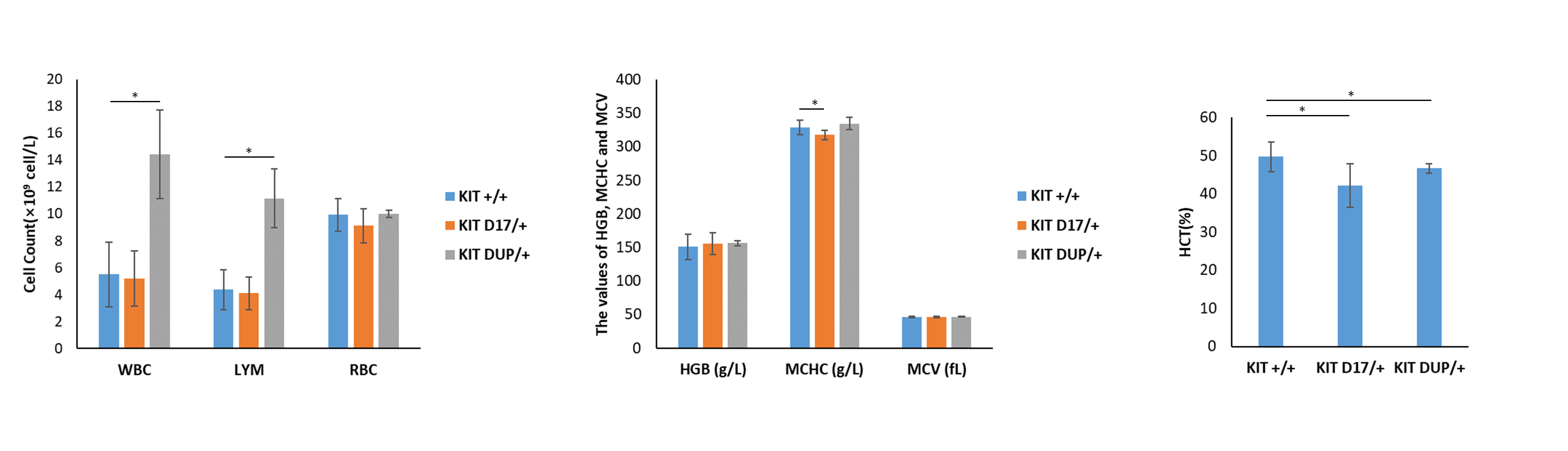

Supplement: Supplementary file 5 [file Image_4.tif]

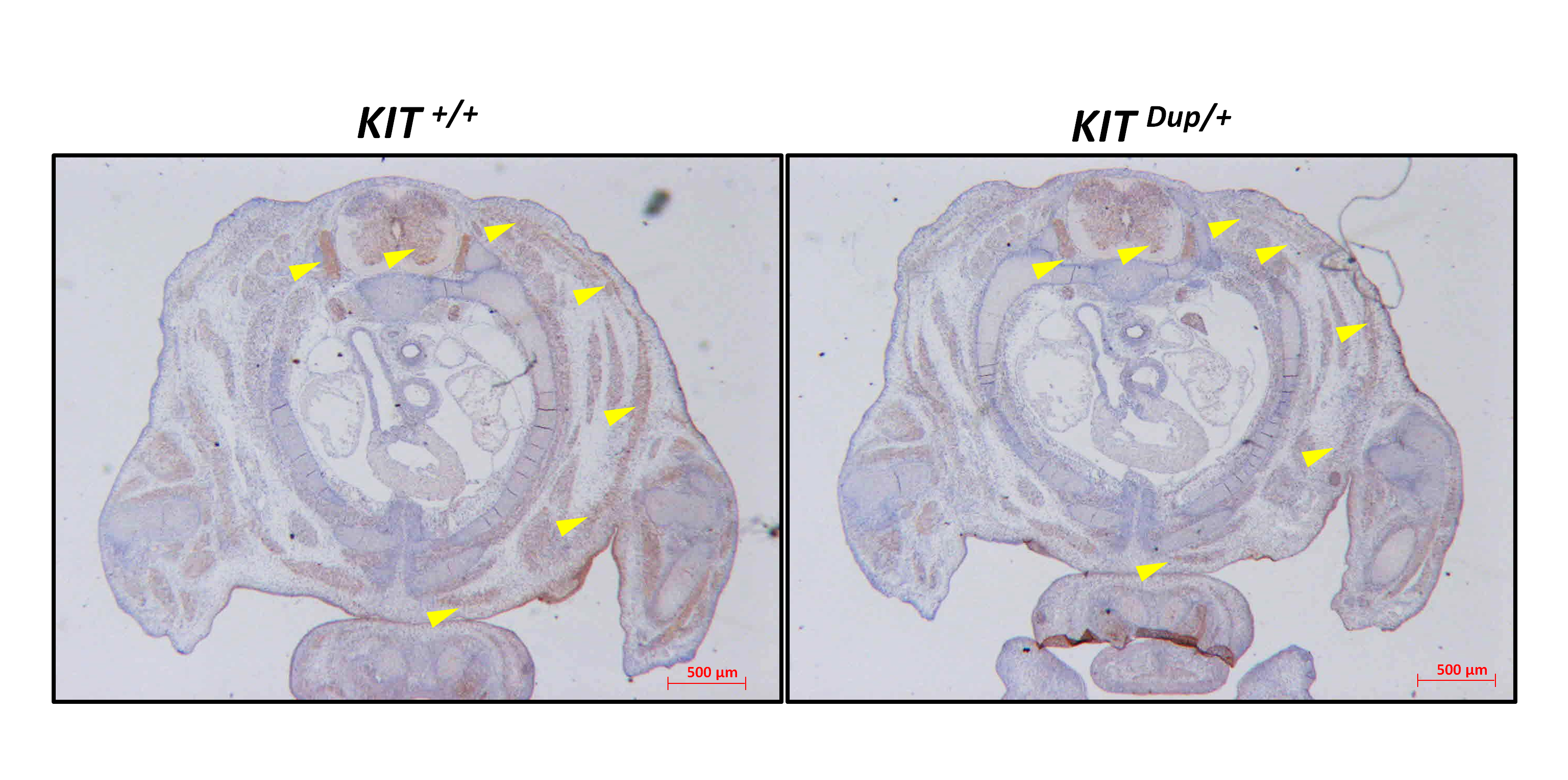

Supplement: Supplementary file 6 [file Image_5.tif]

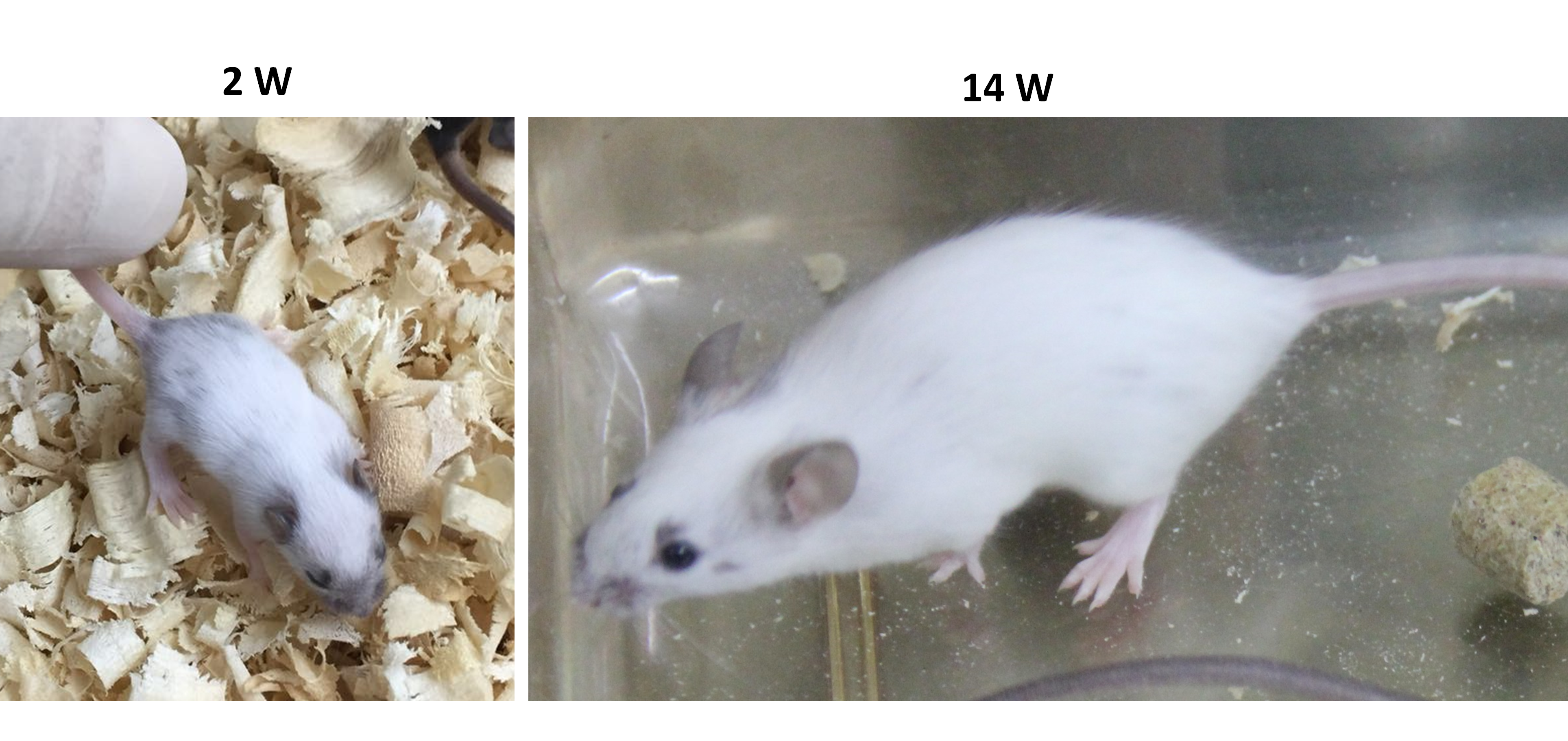

Supplement: Supplementary file 7 [file Image_6.tif]
