# Supplementary material for: Functional Analysis of KIT Gene Structural Mutations Causing the Porcine Dominant White Phenotype Using Genome Edited Mouse Models
Source: Front Genet. 2020 Mar 3;11:138. doi: 10.3389/fgene.2020.00138 (PMC7063667; doi:10.3389/fgene.2020.00138)
Supplement: Supplementary file 8 [file Table_1.docx]

Supplementary Material

**Supplementary Figure S1. Comparison of KIT expression levels in the skin of a dominant white pig breed and black pig breed.** (A) KIT mRNA levels in the skin of Yorkshire (*I*/*I* genotype) and Yuedong black pigs (YDB, black pig with *i*/*i* genotype) as determined by qPCR analysis. (B) Expression levels of KIT in the skin of Yorkshire and YDB were determined by western blot analysis.

**Supplementary Figure S2. Phenotypes of *KIT ^GtoA/+^, KIT ^D17/+^* and *KIT ^Dup/Dup^* mice and identification of gene-edited individuals.** (A) White spots appeared on the abdomen of *KIT ^GtoA/+^* mice*. KIT ^D17/+^* mice presented with a piebald coat on their head and trunk, a vertical white stripe on their forehead, a half loop of white hair on their shoulder blades, and dominant white over their abdominal region. There was no apparent difference between the coat colors of *KIT^+/+^* and *KIT ^Dup/Dup^* mice. The mice were 14 W old. (B) Schematic diagram of the primers for *KIT ^Dup/Dup^* mice identification. (C) PCR identification of gene edited individuals from the offspring of each mouse model. The positive amplification result of *KIT ^Dup/+^* was approximately 870 bp, and the WT had no product. The positive amplification result of *KIT ^GtoA/+^* was approximately 350 bp in size, and the WT had no product. The positive amplification result of *KIT ^D17/+^* was approximately 350 bp, and the WT amplification product was approximately 610 bp. (D) RT-PCR analysis of the *KIT ^Dup/+^* and *KIT ^Dup/Dup^* mice. The truncated PCR product is indicated by an arrowhead.

**Supplementary Figure S3. Identification of mouse peritoneal mast cells through flow cytometry analysis.** KIT (stained by anti-Mouse CD117 APC) and FcεRI (stained by anti-mouse Fc epsilon receptor I alpha) were used as mast cell markers.

**Supplementary Figure S4. Haematological parameters analysis of the tn *KIT ^D17/+^and KIT ^Dup/+^* mouse models.** WBC, white blood cells; LYM, lymphocyte; RBC, red blood cells; HCT, hematocrit; HGB, hemoglobin; MCHC, meancorpuscular hemoglobin concentration; MCV, mean corpuscular volume.

**Supplementary Figure S5. Using KIT as marker to detect melanoblast migration in *KIT ^+/+^* and *KIT ^Dup/+^* mice embryos.** KIT was used as a marker to detect melanoblast migration in *KIT ^+/+^* and *KIT ^Dup/+^* mice embryos (E14.5).

**Supplementary Figure S6. Change in *KIT ^Dup/D17^* mouse coat color over time.** With the increase of age, the gray hairs of the eyelids and hips gradually disappeared.

**Supplementary Figure S7. The sequencing results of *KIT ^D17/+^, KIT ^GtoA/+^* and *KIT ^Dup/+^* mice in the** **F1 generation.**

**Supplementary Table S1. The homozygous *KIT* splice mutation could be lethal.** Oocytes from superovulated *KIT ^D17/+^* females were *in vitro* fertilized with sperm collected from 5 *KIT ^D17/+^* males, and transferred to 10 surrogate females to generate offspring. No *KIT ^D17/D17^* pups were born but 13 *KIT ^+/+^* and 24 *KIT ^D17/+^* pups were obtained.

**Supplementary Table S2. Oligos and primers used in this study.**

**Supplementary Table S3. Haematological parameters measured for the different KIT mouse models.** WBC, white blood cells; LYM, lymphocyte; RBC, red blood cells; HCT, hematocrit; HGB, hemoglobin; MCHC, meancorpuscular hemoglobin concentration; MCV, mean corpuscular volume; PLT, platelet. Data was presented as mean ± SD. Values with different letters within rows are significantly different by at least the 5% level by the Duncan test.
