# Supplementary material for: Functional Analysis of KIT Gene Structural Mutations Causing the Porcine Dominant White Phenotype Using Genome Edited Mouse Models
Source: Front Genet. 2020 Mar 3;11:138. doi: 10.3389/fgene.2020.00138 (PMC7063667; doi:10.3389/fgene.2020.00138)
Supplement: Supplementary file 9 [file Table_2.docx]

| **Class** | **Number** | **Percent (%)** |
| --- | --- | --- |
| Male *KIT ^D17/+^* (sperm donor) | 5 |  |
| Female *KIT ^D17/+^* (oocyte donor) | 10 |  |
| Female ICR (surrogate) | 10 |  |
| offspring | 37 | 100.0 |
| - *KIT ^+/+^* | 13 | 35.1 |
| - *KIT ^D17/+^* | 24 | 64.9 |
| - *KIT ^D17/D17^* | 0 | 0.0 |
