# Supplementary material for: Functional Analysis of KIT Gene Structural Mutations Causing the Porcine Dominant White Phenotype Using Genome Edited Mouse Models
Source: Front Genet. 2020 Mar 3;11:138. doi: 10.3389/fgene.2020.00138 (PMC7063667; doi:10.3389/fgene.2020.00138)
Supplement: Supplementary file 10 [file Table_3.docx]

| # | name | Sequence (5’ – 3’) | Purpose |
| --- | --- | --- | --- |
| 1 | kit-CDS-KI | AGGCATCTTCGTGCACGAGC | sgRNA for *KIT* duplication model |
| 2 | 2651-kit-3S1 | CACGGGATGAAAGTGTTCCG | sgRNA for *KIT* intron 17 G to A model |
| 3 | 2651-kit-5S1 | CAGGAATGATTCGAATTACG | sgRNA for *KIT* intron 17 G to A model |
| 4 | 2098-kit-S1 | GACGTTTATTAAAATTGGGT | sgRNA for *KIT* exon 17 deletion model |
| 5 | 2098-kit-S2 | GATCCTGTGAAACACAAAAC | sgRNA for *KIT* exon 17 deletion model |
| 6 | 2098-kit-S3 | ATAGTATTTCAAACGAAGCG | sgRNA for *KIT* exon 17 deletion model |
| 7 | 2098-kit-S4 | AGAAAGCCTACCGTGACCTTC | sgRNA for *KIT* exon 17 deletion model |
| 8 | kit-dup-ps-F | CAAGATCCGCCACAACATCG | *KIT Dup/+* mice identification primers |
| 9 | kit-dup-ps-R | GGAACTCGCTACCCTGGAATAGGAT |  |
| 10 | kit-d17-ps-F | ACATTCCCAGGTAACTACCAA | *KIT D17/+* mice identification primers |
| 11 | kit-d17-ps-R | TGGCACTTTGAAAACTTCACT |  |
| 12 | kit-GA-ps-F | TCTGAGGCGGAAAGAACCAG | *KIT dup/+* mice identification primers |
| 13 | kit-GA-ps-R | TGGATTGCTAGTTTCAGCCTATCG |  |
| 14 | kit-d17-NT-F | GATTTGACTATTTATAATGCAT | *KIT* D17 mice heterozygotes identification primers |
| 15 | kit-d17-NT-R | GAGGAGGATATTCCTGGCTGC |  |
| 16 | Mouse KI F | CAAGATCCGCCACAACATCG | *KIT* dup mice heterozygotes identification primers |
| 17 | Mouse KI R | GGAACTCGCTACCCTGGAATAGGAT |  |
| 18 | Mouse KI homo F | ATTCTCGAAATGATTTACCTGGGGA | *KIT* dup mice heterozygotes identification (WT allele) primers |
| 19 | Mouse KI homo R | CTGTTTACAGTAGGAGCTACTTTGC |  |
| 20 | kit-QPCR-F | GCCACGTCTCAGCCATCTG | KIT Q-PCR primers |
| 21 | kit-QPCR-R | GTCGCCAGCTTCAACTATTAACT |  |
| 22 | GAPDH-QPCR-F | CCTGAGGCTCTTTTCCAGCC | GAPDH QPCR primers |
| 23 | GAPDH-QPCR-R | TAGAGGTCTTTACGGATGTCAACGT |  |
