# Supplementary material for: Functional Analysis of KIT Gene Structural Mutations Causing the Porcine Dominant White Phenotype Using Genome Edited Mouse Models
Source: Front Genet. 2020 Mar 3;11:138. doi: 10.3389/fgene.2020.00138 (PMC7063667; doi:10.3389/fgene.2020.00138)
Supplement: Supplementary file 11 [file Table_4.docx]

|  | **KIT^+/+^** | **KIT^D17/+^** | **KIT^Dup/+^** |
| --- | --- | --- | --- |
|  | **Mean ± SD (n = 12)** | **Mean ± SD (n =8)** | **Mean ± SD (n = 4)** |
| **WBC (×10^9^/L)** | 5.51±2.41^b^ | 5.21±2.05^b^ | 14.45±3.3^a^ |
| **LYM (×10^9^/L)** | 4.36±1.5^b^ | 4.1±1.21^b^ | 11.15±2.2^a^ |
| **RBC (×10^9^/L)** | 9.92±1.22 | 9.11±1.26 | 10.01±0.29 |
| **HCT (%)** | 49.72±3.92^a^ | 42.18±5.7^b^ | 46.68±1.29^ab^ |
| **HGB (g/L)** | 151.17±19.02 | 155.88±16.11 | 156.25±3.42 |
| **MCHC (g/L)** | 328.75±10.94^ab^ | 317.63±7.19^b^ | 334.75±9.47^a^ |
| **MCV (fL)** | 46.34±0.8 | 46.45±1.12 | 46.63±0.38 |
| **PLT (×10^9^/L)** | 387.25±155.85 | 497.38±196.76 | 541.5±204.58 |
