# Supplementary material for: Functional Analysis of KIT Gene Structural Mutations Causing the Porcine Dominant White Phenotype Using Genome Edited Mouse Models
Source: Front Genet. 2020 Mar 3;11:138. doi: 10.3389/fgene.2020.00138 (PMC7063667; doi:10.3389/fgene.2020.00138)
Supplement: Supplementary file 12 [file Table_5.docx]

**Functional analysis of *KIT* gene structural mutations causing the porcine dominant white phenotype using genome edited mouse models**

**Guangjie Sun, Xinyu Liang, Ke Qin, Yufeng Qin, Xuan Shi, Peiqing Cong, Delin Mo, Xiaohong Liu, Yaosheng Chen^*^, and Zuyong He^*^**

State Key Laboratory of Biocontrol, School of Life Sciences, Sun Yat-Sen University, Guangzhou 510006, China

*** Correspondence:**

Yaosheng Chen

chyaosh@mail.sysu.edu.cn

Zuyong He

zuyonghe@foxmail.com

**Keywords: KIT, Porcine coat color, Melanocyte, Mouse model, Exon 17 deletion, CDS duplication**

**Abstract**

The dominant white phenotype in pigs is thought to be mainly due to a structural mutation in the *KIT* gene, a splice mutation (G > A) at the first base in intron 17 which leads to the deletion of exon 17 in the mature *KIT* mRNA. However, this hypothesis has not yet been validated by functional studies. Here, we created two mouse models, *KIT ^D17/+^* to mimic the splice mutation, and *KIT ^Dup/+^* to partially mimic the duplication mutation of *KIT* gene in dominant white pigs using CRISPR/Cas9 technology. We found that the splice mutation homozygote is lethal and the heterozygous mice have a piebald coat. Slightly increased expression of KIT in *KIT ^Dup/+^*mice did not confer the patched phenotype and had no obvious impact on coat color. Interestingly, the combination of these two mutations reduced the phosphorylation of PI3K and MAPK pathway associated proteins, which may be related to the impaired migration of melanoblasts observed during embryonic development that eventually leads to the dominant white phenotype.

1. **Introduction**

Due to domestication and long term selection, dominant white is a widespread coat color among domestic pig breeds, such as Landrace and Yorkshire (Wiseman, 1986). The dominant white phenotype in domestic pigs is thought to be caused by two structural mutations in the *KIT* gene, (1) a ~450-kb tandem duplication that encompasses the entire *KIT* gene body and the ~150 kb upstream region and (2) a splice mutation at the first nucleotide of intron 17 in one of the *KIT* copies, which leads to the skipping of exon 17 and the production of KIT protein lacking a critical region in the kinase catalytic domain. (Marklund et al., 1998; Giuffra et al., 2002; Pielberg et al., 2002; Andersson, 2010; Rubin et al., 2012).

KIT is a class III tyrosine kinase receptor, encoded by the *KIT* gene. The KIT receptor is expressed on several cell types, including mast cells, hematopoietic progenitors, melanoblasts and differentiated melanocytes (Lennartsson and Rönnstrand, 2012). The binding of its ligand - stem cell factor (SCF), causes KIT to homodimerize, leading to the activation of its intrinsic kinase activity through autophosphorylation of tyrosine residues. KIT has a number of potential tyrosine phosphorylation sites, which interact with multiple downstream signaling pathways, including the PI3K, MAPK, and Src family kinase pathways (Roskoski, 2005; Lennartsson and Rönnstrand, 2012). These pathways are involved in the regulation of cell growth, survival, migration and differentiation (Imokawa, 2004).

The previously mentioned 450-kb large duplication that encompasses the entire *KIT* gene was speculated to confer the patch phenotype in pigs by causing abnormal KIT expression (Giuffra et al., 2002). Based on this, it has been proposed that the duplication occurred first and resulted in a white-spotted phenotype that was selected for by humans. Subsequently the splice mutation occurred and produced a completely white phenotype due to the skipping of exon 17 in the mature transcript removing a crucial part of the tyrosine kinase domain, thus enhancing the defect in *KIT* signaling function (Andersson, 2010), and disturbing the migration of melanocyte precursors, leading to a dominant white coat color (Marklund et al., 1998). This seems reasonable, as normal migration and survival of neural crest-derived melanocyte precursors is dependent on KIT expression and the availability of its ligand (Wehrle‐Haller and Weston, 1997). Loss of function mutations in the *KIT* gene could lead to white coat color in mice, as documented in homozygous *KIT* ^K641E^ mice (Rubin et al., 2005) and the KIT-deficient mouse model W^v^/W^v^ (Moniruzzaman et al., 2007). However, functional analysis of the structural mutations of the *KIT* gene of dominant white pigs still needs to be carried out to confirm this hypothesis. We created mouse models mimicking the splice mutation and *KIT* upregulation mutation using CRISPR/Cas9 technology to investigate the underlying genetic mechanism of the dominant white phenotype (Bernex et al., 1996).

1. **Article types**

Original Research

1. **Materials and Methods**

**3.1 Establishment of mouse models**

All mouse models were established in the C57BL/6 background by the Model Animal Research Center of Nanjing University (China) as described in a previous report (Tao et al., 2009), with minor modifications. Briefly, C57BL/6 mice were kept in a 12/12 h light/dark cycle. To produce zygotes for pronuclear injection, female mice were injected with 5 IU pregnant mare's serum gonadotropin (PMSG), and 46–48 h later were injected with 5 IU hCG to induce ovulation in 10-12 h. Following the hCG injection, the females were put together with the male mice in single cages overnight. Fertilized oocytes were isolated from the oviducts for pronuclear injection. To generate the *KIT ^Dup/+^* and *KIT ^GtoA/+^*mouse models, Cas9 mRNA, sgRNA and the appropriate donor plasmid (Figure 1B) were injected into the zygote pronuclei. To generate the *KIT ^D17/+^*mouse model, Cas9 mRNA and a pair of sgRNAs (Figure 1B) were injected. All sgRNA sequences are listed in S2 table. The injected zygotes were transferred into the oviducts of surrogate recipient female mice to deliver genome-edited pups. *KIT ^Dup/D17^* mice were obtained by mating *KIT ^D17/+^*females with *KIT ^Dup/+^* males because *KIT ^D17/+^*males are infertile.

All procedures were performed in strict accordance with the recommendations of the Guide for the Care and Use of Laboratory Animals of the National Institutes of Health. The protocol was approved by the Institutional Animal Care and Use Committee (IACUC), Sun Yat-sen University (Approval Number: IACUC-DD-16-0901).

**3.2 Mouse genotyping**

Mice genotypes are identified by polymerase chain reaction (PCR). The tail of 1 week old mice are cut off to extract DNA by using a tissue DNA extraction kit (OMEGA). Primers used in the PCR were summarized in table S2.

The PCRs were carried out using the GeneStar^TM^ PCR Mix system. Each PCR reaction mix contained 1× GeneStar^TM^ PCR Mix buffer, 1.0 µM of each primer and approximately 100 ng DNA template. The thermocycler program was an initial 5 min hold at 95 °C, followed by 35 cycles of 30 sec at 95 °C, 30 sec at 60 °C, and 30 sec at 72 °C, finishing with 10 min extension at 72 °C.

In order to determine the mutant sequences, the PCR products were recovered with an OMEGA DNA Gel Recovery Kit, cloned into the pMD-18T vector (TAKARA) and transformed into DH5α competent cells. The plasmids then were purified from *E. coli* cells for Sanger sequencing.

**3.3 Isolation and culture of peritoneal cell derived mast cells**

Isolation and culture of peritoneal cell derived mast cells was performed as previously described (Meurer et al., 2016) with minor modifications. Briefly, 5 mL of PBS and 2 mL of air was injected into the peritoneal cavity of 14 week old mice. The injected mice were then carefully shaken in the palm for 5 mins and the cell-containing fluid of the peritoneal cavity was gently collected in a plastic Pasteur pipette. After centrifugation, cells were resuspended and cultured in DMEM medium supplemented with serum, cytokines IL-3 (CP39; novoprotein), and SCF (C775; novoprotein). After culture for 10 days, CD117 and FceR1 makers were used to determine whether cultured cells are mast cells by flow cytometry analysis using a Beckman Coulter Gallios™ Flow Cytometer. For surface staining, the cells were stained with APC anti-Mouse CD117 (Thermo Fisher Scientific Cat# 17-1171-81, RRID:AB_469429) and FITC anti-mouse Fc epsilon receptor I alpha (FceR1) (Thermo Fisher Scientific Cat# 11-5898-82, RRID:AB_465308) at room temperature for 30 minutes, washed with PBS, and then re-suspended in PBS.

**3.4 Histological and immunohistochemical analysis of tissue sections**

Skin tissues and embryos were fixed overnight in 10% (w/v) paraformaldehyde with 0.02 MPBS (pH 7.2) at 4 °C, processed, and mounted in paraffin, they were then serially cut into 5μm-thick sections with a Rotary Microtome (MICROM). The histological sections were stained with hematoxylin and eosin (H&E) and then observed and photographed with a fluorescent microscope (Zeiss). For immunohistochemistry experiments, sections were treated with 3% H_2_O_2_ to quench endogenous peroxidase activity and then treated with 5% bovine serum albumin to block nonspecific protein binding sites. Sections were incubated with a primary antibody overnight at 4 °C and then stained with an anti-Rabbit HRP-DAB Cell and tissue staining kit (R&D, CTS005). Detection was followed by TSA plus Fliorescein (Perkinelemer, NEL741001KT). All antibodies including KIT (Abcam Cat# ab47587, RRID:AB_868771), Phospho-KIT (Try719) (Cell Signaling Technology Cat# 3391, RRID:AB_2131153), Green Fluorescent Protein (Millipore Cat# AB3080P, RRID:AB_2630379), DCT (Abcam Cat# ab74073, RRID:AB_1524517), Erk1/2 (Cell Signaling Technology Cat# 4695, RRID:AB_390779), Akt (Cell Signaling Technology Cat# 9272, RRID:AB_329827) Phospho-Akt (Cell Signaling Technology Cat# 4060, RRID:AB_2315049) and Phospho-Erk1/2 (Cell Signaling Technology Cat# 8544, RRID:AB_11127856) were diluting 1:200 with PBS.

**3.5 qPCR**

To detect levels of gene expression, total RNA was prepared using TRIzol (Invitrogen) extraction followed by DNase (Ambion) treatment, and reverse transcribed with a Reverse Transcription System (Promega) following the manufacturer’s instructions. The resulting total cDNA was analyzed quantitatively using a FastStart Universal SYBR Green Master kit (Roche) with primers for *KIT, ERK, AKT, PLCG* and *DCT*. Expression profiles were tested in triplicate on at least three mice on an LC480 instrument (Roche). Data were analyzed using the comparative Ct (ΔΔCt) method and one-tail, unpaired student T test (significance cutoff p<0.01). Gene expression levels were normalized to the housekeeping gene glyceraldehyde 3- phosphate dehydrogenase (GAPDH).

**3.6 Western blot analysis**

Proteins were extracted using Lysis Buffer (Key GEN), and protein concentration was determined with a PierceTM BCA Protein Assay Kit (Thermo). Protein (300 ng) was run on a 10% SDS gel and electrotransferred onto a PVDF membrane (Roche). After blocking for 1 h with 3% BSA in PBS, the membrane was incubated with primary antibodies overnight at 4 °C.

The rabbit anti-KIT antibody (Abcam Cat# ab47587, RRID:AB_868771), rabbit anti-Phospho-KIT (Try719) antibody (Cell Signaling Technology Cat# 3391, RRID:AB_2131153), rabbit anti-Green Fluorescent Protein antibody (Millipore Cat# AB3080P, RRID:AB_2630379), rabbit anti-DCT antibody (Abcam Cat# ab74073, RRID:AB_1524517), rabbit anti-Erk1/2 antibody (Cell Signaling Technology Cat# 4695, RRID:AB_390779) and rabbit anti-Akt antibody (Cell Signaling Technology Cat# 9272, RRID:AB_329827) were diluted 1:1000, rabbit anti-Phospho-Akt antibody (Cell Signaling Technology Cat# 4060, RRID:AB_2315049) and rabbit anti-Phospho-Erk1/2 antibody (Cell Signaling Technology Cat# 8544, RRID:AB_11127856) were diluted 1:2000, rabbit anti-GAPDH antibody (Bioworld Technology Cat# AP0063, RRID:AB_2651132) was diluted 1:5000 with PBS. Following three 10 min washes with TBST, the membrane was incubated with 1:5000 goat anti-rabbit secondary antibodies (Abcam Cat# ab6721, RRID:AB_955447) for 1 h at room temperature. Protein bands were visualized using a Kodak image station 4000MM/Pro (Kodak), according to the manufacturer’s instructions, and exposed to FD bio-Dura ECL (FD, FD8020). Protein levels were standardized by comparison with GAPDH.

**3.7 Blood parameters analysis**

Approximately 100 μL blood collected from the tails of each mouse was subjected to cell counting by XT-1800iV (Sysmex) haematology analyzer.

**3.8 Statistical analysis**

All experiments consisted of at least three independent replicates. Quantitative data are shown as the mean± SEM. Statistically significant differences were analyzed with the t-test or one way analysis of variance, followed by Duncan’s post hoc test. A p-value < 0.05 indicates statistical significance.

1. **Results**

**4.1 Splice mutation but not the CDS duplication of *KIT* gene leads to altered coat color**

In pigs, the wild-type KIT allele is recessive and denoted as *i*. Previous studies have claimed that the semidominant *I^P^* and the dominant *I* alleles of KIT confer the patch and dominant white phenotype, respectively (Figure 1A). The *I* allele is a 450-kb large duplication (two to three copies) that encompasses the entire *KIT* gene and at least one of the *KIT* copies carries a splice mutation (G>A at the first base in intron 17), causing exon skipping and the expression of a KIT protein lacking an essential part of the tyrosine kinase domain. *I^P^* allele consists of the 450-kb duplication but not the splice mutation (Rubin et al., 2012). To investigate the effects of *KIT* gene mutations on coat color, we created genome edited mouse models to mimic the structural mutations of porcine KIT in the C57BL/6 strain, which is black, and broadly used in coat color study (Slominski et al., 1994).

Through qPCR and Western blot analysis, we found that the expression of KIT in the skin of Yorkshire pigs (*I*/*I*) is significantly higher than that in Yuedong black pig (*i*/*i*) (Figure S1). In order to investigate the effect of increased KIT expression on coat color, we knocked in the CDS of the *KIT* gene linked with an enhanced green fluorescent protein (EGFP) reporter via a self-cleaving 2A peptide to facilitate its subsequent identification (Figure 1B). The heterozygous *KIT* duplication mouse model was denoted as *KIT ^Dup/+^*. Western blot analysis demonstrated that EGFP was extensively expressed in the skin of *KIT ^Dup/+^* mice, as compared with the wild-type control *KIT^+/+^*, which implies that the inserted *KIT* CDS was correctly expressed, as the 2A peptide strategy allows the co-expression of KIT proteins and EGFP from an integrated single vector (Figure 1D). We found that *KIT ^Dup/+^* mice did not have the patch phenotype, as no obvious difference was observed in the coat color of *KIT ^Dup/+^* and *KIT^+/+^* mice (Figure 1C).

To mimic the splice mutation, we substituted the first nucleotide (G) of *KIT* gene intron 17 with A through CRISPR/Cas9 mediated homologous recombination (Figure 1B). The heterozygous mouse model was denoted as *KIT ^GtoA/+^*. Extensive screening of the offspring implies that the homozygous *KIT* with this single mutation could be lethal, as no surviving *KIT ^GtoA/GtoA^* individual was identified (data not shown). Unlike *KIT^+/+^* mice big white spots appeared on the abdomen of *KIT ^GtoA/+^* mice (S2 Figure). To determine whether the G to A mutation at the first nucleotide of intron 17 of *KIT* gene leads to the skipping of exon 17, RT-PCR was carried out on RNA isolated from the skin of *KIT ^GtoA/+^* mice. To our surprise, the results showed that exon 17 was not removed from the mRNA transcript, and that a small percent of the transcripts contained part of intron 17, as determined by Sanger sequencing (Figure 1F & G). As this model does not mimic the splice mutation well, we did not use it in subsequent studies.

Therefore, in order to create a mouse model that mimics exon 17 skipping, we directly deleted exon 17 in the genome using paired sgRNAs targeting intron 16 and intron 17 (Figure 1B). The heterozygotes were denoted as *KIT ^D17/+^*. An extensive screening of the offspring implies that homozygous *KIT* gene with exon 17-deletion could be lethal, as no surviving *KIT ^D17/D17^* individuals were identified. In addition, IVF fertilization of oocytes from *KIT ^D17/+^* females with sperm from *KIT ^D17/+^* males no surviving *KIT ^D17/D17^* individuals were obtained (S1 Table). This confirms the previous speculation that the *I^L^* allele (single copy of the *KIT* gene with the splice mutation) could be lethal in pigs, as it is not found in the global pig population (Rubin et al., 2012). RT-PCR analysis of the skin tissue from *KIT ^D17/+^* mice indicates that exon 17 is removed from the mature transcript (Figure 1E), and this was confirmed by Sanger sequencing (Figure 1G). Interestingly, compared with *KIT^+/+^*, *KIT ^D17/+^* mice presented with a piebald head and trunk, a vertical white stripe on the forehead, a half loop of white hair on the shoulder blade area, and their entire abdomens (Figure 1D & S2 Figure).

The results of sequencing the F1 generation of the three mouse models and PCR identification of the edited individuals from the offspring of those mouse models is shown in the supplementary materials (S1 Figure & S7 Figure).

**4.2 The *KIT* splice mutation significantly reduces melanin** **accumulation**

Histological analysis (Fontana-Masson staining) of the back skin of 5-week old mice, revealed that similarly to *KIT ^+/+^* control mice, the hair follicles of both *KIT ^Dup/+^*and *KIT ^D17/+^* mice are long and the dermal papilla in the hair bulb is completely coated by the matrix, the keratogenous zone region is clearly visible and is connected to the hair shaft and dermal papilla, and fibrous tract is substantially invisible in the skin (Figure 2). These results indicate that similar to the *KIT ^+/+^* control mice, the hair follicles of 5-week-old *KIT ^Dup/+^*and *KIT ^D17/+^* mice are in the growing stage (anagen V or VI), which is advantageous for observations of hair follicle shape, and melanin distribution since melanin synthesis is more active during this stage (Hou et al., 2016). The hair follicle shape implies that both the splice mutation and CDS duplication mutation did not impair the hair follicle development significantly.

No obvious difference was observed in the hair follicle melanin content of *KIT ^Dup/+^* and *KIT ^+/+^* mice (Figure 2). Almost no melanin is observed in the hair follicles in the white coat area, and the melanin level of hair follicles in the black coat area of *KIT ^D17/+^* is significantly lower then that in *KIT ^+/+^* mice (indicated by the yellow arrow head). Therefore, slightly increased expression (approximately 1.1-fold) of KIT in *KIT ^Dup/+^* did not impair the melanin accumulation, whereas the splice mutation significantly impaired melanin accumulation in hair follicles.

**4.3 The piebald coat color of *KIT ^D17/+^* mice is caused by the reduction of melanocytes**

The reduction in the melanin content of hair follicles may be due to a reduction in the number of melanocytes or their synthesis of melanin. To determine which factor causes the piebald coat of *KIT ^D17/+^* mice, we used the KIT protein as a marker to detect the distribution and amounts of melanocytes in the hair follicles of 5-week-old mice. Compared with the *KIT ^+/+^* control mice, we found that immunohistochemical staining of KIT was decreased in the hair follicles of black coat area of *KIT ^D17/+^*mice, and decreased even more significantly in the white coat region (Figure 3A). This was confirmed by qPCR and Western blot analysis of the skin tissues (Figure 3B and 3C). In theory, the deletion of exon 17 should not affect the expression level of *KIT*, thus the reduced *KIT* expression level in skin tissue should be due to reduced melanocyte number. qPCR analysis of the isolated peritoneal cell derived mast cells indicated that deletion of exon 17 did not affect the expression level of *KIT* (Figure 3B & S3 Figure). The reduced expression of *KIT* together with another two marker genes (DCT and Melan A) by melanocytes in *KIT ^D17/+^* mice (Figure 3C) suggested that the *KIT* splice mutation could lead to a reduction in number of melanocytes in mice hair follicles.

Unlike the *KIT ^D17/+^*mice, the *KIT ^Dup/+^* mice contained an additional copy of the *KIT* CDS, which in theory could lead to increased *KIT* expression. qPCR analysis of the isolated mast cells confirmed that the *KIT* expression was higher in *KIT ^Dup/+^* mice than that in *KIT ^+/+^* control mice (Figure 3B). However, the expression level of KIT in the skin of *KIT ^Dup/+^* mice was not significantly different from that in the *KIT ^+/+^* control mice as revealed by immunohistochemical and qPCR analysis, the expression of KIT mRNA in KIT Dup/+ mice was 1.4-fold increased than KIT +/+ , the expression of KIT in KIT Dup/+ mice was 1.1-fold increased than KIT +/+ (Figure 3A & B), but was higher as measured by Western blot analysis (Figure 3C). In addition, the expression level of melanocyte marker gene DCT was not affected, but another melanocyte marker gene MelanA was significantly increased in the skin of *KIT ^Dup/+^* mice (Figure 3C). These results suggested that the slightly increased expression of KIT in *KIT ^Dup/+^* did not significantly affect the number of melanocytes in the skin, but may affect melanin synthesis.

Interestingly, we observed that the distribution of melanocytes in hair bulbs was broader in *KIT ^D17/+^*mice than that in *KIT ^+/+^* control mice. This phenomenon was more obvious in the white coat than that in the black coat area (Figure 3A). In addition, we found that the distribution of melanocytes was relatively broader in the hair bulbs of *KIT ^Dup/+^* mice than that in *KIT ^+/+^* control mice (Figure 3A). Previous studies considered only melanocytes close to the dermal papilla capable of secreting and providing melanin to the hair (Botchkareva et al., 2003). We speculate that the altered distribution of melanocytes in both *KIT* splice mutation and CDS duplication mutation mice may have an impact on melanin accumulation.

**4.4 The *KIT* splice mutation impairs the kinase activity of the KIT protein and affects embryonic melanoblast migration and** **hematological parameters**

Previous studies speculated that *KIT* mutations in dominant white pigs could disturb the migration of melanocyte precursors melanoblasts during embryonic development (Marklund et al., 1998). In order to determine whether the splice mutation or the CDS duplication impairs the migration of melanoblasts during embryonic period, we stained the *KIT* protein as a marker of melanoblasts in the transverse section mice at E14.5. We found no obvious differences in the location of the melanoblasts of *KIT ^Dup/+^*and *KIT ^+/+^* mice (S5 Figure). Though there was no difference in the distribution of melanoblasts near the neural tube in *KIT ^D17/+^* and *KIT ^+/+^* mice, the number of melanoblasts in the dorsolateral migration pathway, near the forelimb and the abdomen epidermis was significantly reduced (Figure 4A). This indicates that the slightly increased expression of KIT alone did not impair the migration of melanoblasts, in contrast, the splice mutation significantly impaired the migration of melanoblasts at the embryonic stage. However, it does not completely block the migration process, so a certain number of melanoblasts could migrate to their destination positions causing the piebald phenotype.

We observed that, compared with the *KIT ^+/+^* control mice, the color of the black hair of *KIT ^D17/+^* mice became significantly lighter as the mice grew older. Measurement of the blackness of mouse hair showed that the blackness of the black hair of *KIT ^D17/+^* mice was comparable to that of *KIT ^+/+^*mice at 2 W, however, the blackness of the black hair of *KIT ^D17/+^* mice decreased dramatically by 14 W, and was close to that of the white hair of the *KIT ^D17/+^* mice (Figure 4B). Interestingly, we found that the blackness of the hair of *KIT ^Dup/+^*mice was relatively lower than that of *KIT ^+/+^*mice at 2 W, but became comparable to them at 14 W (Figure 4B). Thus, we speculate that the *KIT* splice mutation may affect the renewal or melanin synthesis function of melanocytes in mice, which in turn causes the blackness of hair to decrease rapidly with age. Western blot analysis of the skin tissue was carried out to examine whether the impaired melanoblast migration and melanocyte renewal is caused by altered kinase activity of the KIT protein receptor. We found that the splice mutation significantly reduced KIT protein phosphorylation (Figure 3C & D), indicating this mutation could lead to impaired autophosphorylation of the KIT protein. However, both the expression level and phosphorylation of AKT, a key protein of the PI3K pathway, was not impaired by the *KIT* splice mutation. Additionally, the expressions of ERK1/2, key proteins of the MAPK pathway, were not affected by the splice mutation, but ERK1/2 phosphorylation was slightly increased (Figure 3C & D). This result is confusing. Therefore, we further analyzed the expression and phosphorylation levels of AKT and ERK1/2 in follicles by immunohistochemical (IHC) analysis. The amount of the target protein was determined using the Combined Positive Score (CPS), which is the number of target protein stained cells divided by the total number of viable cells, multiplied by 100. The results revealed that the expression levels of both AKT and ERK1/2 in hair follicles were not affected by the *KIT* splice mutation (Figure 4C), however, the phosphorylation levels of these proteins decreased significantly in follicle from the black coat region of *KIT ^D17/+^*mice, and phosphorylated AKT and ERK1/2 could barely be detected in follicles from the white coat region of *KIT ^D17/+^*mice (Figure 4C). Western blot results indicate that the *KIT* CDS duplication increased phosphorylation of the KIT protein approximately 1.2-fold (Figure 3C). This is probably due to the level of the KIT protein has increased 1.1-fold. Similar to the splice mutation, the CDS duplication mutation did not affect either the expression or phosphorylation level of AKT. It also did not affect the expression level of ERK1/2, but slightly increased their phosphorylation (Figure 3C & D). IHC analysis revealed that neither the expression level nor the phosphorylation levels of AKT and ERK1/2 in the follicle of *KIT ^Dup/+^*mice was significantly affected (Figure 4C). As AKT and ERK1/2 are involved in the PI3K and MAPK pathways, respectively, which are responsible for melanoblast migration and differentiation, and melanin synthesis in melanocytes, the impaired melanoblast migration and accelerated hair greying in *KIT ^D17/+^*mice is likely related to impaired KIT kinase function caused by the splice mutation of *KIT*.

Impaired signaling by the PI3K and MAPK pathways could affect haematological parameters. Through blood parameters analysis, we found that the number of red blood cells (RBCs) and the mean corpuscular hemoglobin concentration (MCHC) decreased slightly in KIT^D17/+^ mice, with hematocrit (HCT) decreasing significantly (Table S3 and Figure S4), implying the possibility of mild anemia, which is similar to the results found in a mouse model with a KIT loss of function mutation (Ruan et al., 2005). Our results thus indicate that the KIT splice mutation could impair erythropoiesis in mice, implying altered PI3K and MAPK signaling in RBC progenitors (Kent et al., 2008). In addition, we found that the number of white blood cells (WBCs) and lymphocytes (LYMs) was over 2-fold higher in *KIT^Dup/+^* mice than that in WT mice (Supplementary Table S3 and Figure S4). A previous study found that a gain-of-function KIT mutation promoted myeloid progenitor expansion, and lead to an increased number of WBCs (Deshpande et al., 2013). Therefore, unlike the situation in skin tissue, the KIT CDS duplication may have a stronger effect on PI3K and MAPK signaling in the haematological system, thus leading to an increase in the number of WBCs.

**4.5 The combination of the splice mutation and CDS duplication of *KIT* severely impaired melanoblast migration during embryonic development, and produced the dominant white phenotype**

*KIT ^Dup/+^*males and *KIT ^D17/+^*females were crossed to produce *KIT ^Dup/D17^* offspring as determined by PCR analysis of the deleted exon 17 and integrated EGFP reporter (Figure 5A). Interestingly, the *KIT ^Dup/D17^* mice had a coat color resembling the porcine dominant white phenotype: except for a few gray hairs appearing near the eyelids and hips, the whole body was covered with white hairs (Figure 5B). With increasing age, the gray hairs of the eyelids and hips gradually disappeared (S6 Figure). Through histological observation of the back skin of *KIT ^Dup/D17^* mice, we found that melanin is hardly visible in the hair follicles (Figure 5C). However, no morphological differences were observed between the follicles of *KIT ^Dup/D17^* and *KIT ^+/+^* control mice. The hair follicles of 5-week-old *KIT ^Dup/D17^*mice showed characteristics typical of the growing stage (anagen V or VI) (Figure 5C). This indicates that the compound mutation did not affect the development of the hair follicles, but severely impaired the accumulation of melanin in hair follicles.

We suspected that similar to *KIT ^D17/+^*mice, the decreased melanin accumulation in the hair follicles of *KIT ^Dup/D17^* mice may be caused by a reduction in the number of melanocytes in the hair follicles. qPCR analysis of mast cells isolated from *KIT ^Dup/D17^* mice showed that the compound mutation led to improved expression levels of *KIT* (Figure 5D), mainly due to integration of an additional copy of the *KIT* CDS. In contrast, the expression of *KIT* in skin tissue of *KIT ^Dup/D17^* mice was significantly decreased as determined by qPCR analysis (Figure 5D), this was further confirmed by Western blot analysis (Figure 5E). The reduced expression of *KIT* together with that of three melanocyte marker genes (DCT, MelanA and S100) in the skin tissue of *KIT ^Dup/D17^* mice (Figure 5E) suggested that the *KIT* compound mutation could lead to fewer melanocytes in mice hair follicles, which may contribute to the decreased melanin accumulation.

IHC analysis of the skin tissue confirmed that there are significantly fewer melanocytes in the hair follicles of *KIT ^Dup/D17^* mice than that in *KIT ^+/+^* control mice, with only a few layers of melanocytes visible in close proximity to the dermal papilla (Figure 5C).

A few gray hairs were found in the whole white background of *KIT ^Dup/D17^* mice at young age, but they disappeared gradually as shown by the hair blackness analysis (Figure 5G & S6 Figure). This implies that the compound mutations of *KIT* gene may affect the renewal of melanocytes in hair follicles.

To investigate the molecular mechanism underlying the effect of the compound *KIT* mutation gene on coat color, the kinase function of the phosphorylation of KIT protein was determined by Western blot analysis of the skin tissue of *KIT ^Dup/D17^* mice. The results showed that the phosphorylation KIT in the skin of *KIT ^Dup/D17^* mice was significantly lower than that in *KIT ^+/+^* control mice (Figure 5E). Both the expression and phosphorylation levels of AKT, a key protein of the PI3K pathway, decreased significantly. Though the expressions of ERK1/2, key proteins of the MAPK pathway, were not affected, the phosphorylation of ERK1/2 decreased dramatically (Figure 5E). These results indicate that the *KIT* compound mutation substantially impaired the signaling function of KIT protein receptor on PI3K and MAPK pathways. However, to our surprise, further IHC analysis of the hair follicles showed that phosphorylation of AKT and ERK1/2 in *KIT ^Dup/D17^* as measured by CPS does not seem to be affected by the compound mutations (Figure 5F). This may imply a complex interaction between the splice mutation and CDS duplication of the *KIT* gene.

The compound *KIT* mutation impaired the signaling function of the KIT protein, which in turn could affect embryonic melanoblast migration. Therefore, we conducted IHC analysis of the distribution of melanoblasts in the transverse section of *KIT ^Dup/D17^* mice at E14.5 by staining the marker protein KIT. The results showed that compared with the *KIT ^+/+^*control mice, the number of melanoblasts in the embryo of *KIT ^Dup/D17^* mice increased significantly in the neural tube, and the number of melanoblasts in the dorsolateral migration pathway, the forelimb epidermis and the abdomen epidermis decreased significantly (Figure 5H). This indicates that the compound *KIT* mutation severely blocked embryonic melanoblast migration, leading to increased accumulation of melanoblasts in the neural tube of the *KIT ^Dup/D17^* mice, and a coat color resembling the porcine dominant white phenotype.

1. **Discussion**

In our study, we used CRISPR/Cas9 technology to create mice models to mimic the structural *KIT* mutations of dominant white pigs. We used the *KIT ^D17/+^* mice model to study the effect of the splice mutation, which caused exon 17 skipping, on coat color and the *KIT ^Dup/+^* mice model to partially examine the impact of KIT overexpression caused by *KIT* gene duplication in the porcine *I* allele. We found that the slightly overexpression (approximately 1.1-fold ) KIT caused by *KIT* CDS duplication did not influence mouse coat color but deletion of *KIT* exon 17 turned the black mouse hair piebald. Molecular experiments demonstrated that the *KIT* exon 17 deletion reduced the kinase function of KIT and impairs its signaling transduction to the PI3K and MAPK pathways, which are involved in melanoblast migration, blocking the migration of some melanoblasts from the dorsal to ventral regions during embryo development, resulting in a piebald coat in mice. Interestingly, the combination of these two mutations leads to a nearly complete white phenotype resembling the dominant white phenotype in pigs. This is probably due to severely blocked migration of melanoblasts from the neural tube to other regions of the body (Figure 6).

KIT plays key roles in driving the migration of melanocytes from the neural crest along the dorsolateral pathway to their final destination in the skin (Besmer et al., 1993). Mutations of *KIT* are associated with the dominant white coat color of several mammalian species. In pig, the dominant white coat color is determined by the duplication of an approximately 450-kb region encompassing the entire *KIT* gene (copy number variation, CNV) and by the presence of a splice mutation in intron 17 in one of the duplicated copies, which causes the skipping of exon 17 (Pielberg et al., 2002). The *KIT* allele with two normal *KIT* copies has been thought to cause pigmented regions (patches) in white pigs (Pielberg et al., 2002). In addition, the *KIT* allele carries a single copy of a mutated *KIT* gene (with splice mutation) that should be lethal if homozygous (Pielberg et al., 2002; Johansson et al., 2005). However, these perspectives had not been validated by functional studies. Our functional study confirmed that the homozygous splice mutation in *KIT* is lethal, as *KIT ^D17/D17^* mice could not be obtained. However, whether the lethal is due to anemia in the embryonic stage as previously found in a *KIT* defect mouse model needs to be further validated. We also found that the slightly increase in KIT expression (approximately 1.1-fold) due to the CDS duplication may not contribute to the patch phenotype, as both *KIT ^Dup/+^* and *KIT ^Dup/Dup^* mice coat color was basically indistinguishable from that of the *KIT^+/+^* control mice (Figure 1C & S1 Figure).

The expression of KIT was significantly higher in the skin of Yorkshire pigs (*I*/*I*) than that in Yuedong Black pigs (*i*/*i*) (S1 Figure). However, in our *KIT ^Dup/+^* mouse model, the expression of KIT was significantly higher in mast cells (Figure 3B), and had a significant effect on WBC number (Figure S4 and table S3), though it has only slight effects in skin tissue (Figure 3C). The slightly increased level of KIT protein from the additional copy of the *KIT* CDS seems to have minimal effect on the signaling function of the KIT protein receptor on the PI3K and MAPK pathways in skin tissue (Figure 3C), and thus had no obvious effect on melanoblast migration, melanocyte and follicle development, or melanin synthesis. This may indicate that merely slightly up-regulating the expression of KIT does not affect coat color.

It should be noted that our *KIT ^Dup/+^* mice do not fully mimic the *I^P^* allele in pigs. In contrast to the large 450 kb KIT duplication in the *I^P^* allele only the *KIT* CDS was inserted in our KIT duplication mouse model , the large fragment of regulatory regions was not included. The duplicated copy in the *I^P^* allele may lack some regulatory elements located more than 150 kb upstream of the *KIT* gene body (Giuffra et al., 2002). This regulatory mutation may lead to dysregulated expression of one or both copies of KIT and thus contribute to the patch phenotype. In dominant white pigs, the copy number of *KIT* tends to increase by 4 to 6 times. Therefore, whether the patch phenotype is caused by the *I^P^* allele needs to be further validated using other animal models which fully mimic the regulatory mutation and overexpression of *KIT* gene.

Exon 17 of *KIT* encodes 790-831 amino acids of the KIT protein receptor, a highly conserved region of the tyrosine kinase domain, which contains the Tyr 823 residue that is conserved in almost all tyrosine kinases, and which is phosphorylated during KIT activation where it is thought to stabilize KIT tyrosine kinase activity (Roskoski, 2005). The splice mutation leading to lack of this region was previously considered to be responsible for impaired KIT signal transduction, and thus the severe defect in melanocyte precursor migration and survival. Our IHC analysis of the skin tissue confirmed that the splice mutation impaired KIT signal transduction to the PI3K and MAPK pathways (Figure 4C). The PI3K pathway regulates cell growth, proliferation, differentiation and survival, and MAPK regulates cell proliferation and apoptosis. In addition, the MAPK pathway is also responsible for phosphorylating and activating MITF, which in turn activates the transcription of several important genes involved in melanin synthesis, such as Tyrosinase, TRP and TRP2 (Lin and Fisher, 2007). The impaired embryonic melanoblast migration (Figure 4A), and the reduced number of melanocytes and melanin accumulation in the hair follicles (Figure 3A) in *KIT ^D17/+^* mice, could be attributed to the impaired PI3K and MAPK signaling induced by the splice mutation.

A previous study proposed an evolutionary scenario whereby *KIT* duplication occurred first resulting in a white-spotted phenotype, then the splice mutation occurred producing a completely white phenotype. The presence of one normal *KIT* copy in *I* ensures that white pigs have a sufficient amount of KIT signaling to avoid severe pleiotropic effects on hematopoiesis and germ-cell development (Andersson, 2010). We expected that the CDS *KIT* duplication would restore the splice mutation in *KIT ^Dup/D17^* mice. However, the combination of these two mutations leads to severely impaired PI3K and MAPK signaling (Figure 5E & 5F), melanoblast migration (Figure 5H), and a reduction in melanocyte number and melanin accumulation (Figure 5 H) resulting in a nearly completely white coat (Figure 5B). Thus the *KIT* duplication mutation does not seem to rescue the splice mutation. The underlying mechanism of the interaction between these two mutations could be very complicated. As activation of the intrinsic kinase activity of the KIT receptor is dependent on forming a homodimer by binding the SCF ligand. Thus we speculated that increased expression of the normal form of KIT protein in *KIT ^Dup/D17^* mice may increase the chance of formation of a KIT/KIT ^D17^ dimer upon binding SCF compared with that in *KIT ^D17/+^* mice. Given that the amount of SCF ligand is limited, more KIT/KIT ^D17^ dimer presented on the melanoblast cell surface may significantly reduce the activation of the PI3K and MAPK signaling pathways, resulting in more severely impaired melanoblast migration, and a more pronounced change in coat color (Figure 6).

Through observation of the light and electron micrographs of a section of skin, a previous study concluded that melanocytes and their precursors were absent in the hair bulb of dominant white (*I*) pigs, and that the dominant white color in the pigs is due to a defect in the development of melanocytes (Moller et al., 1996). However, we found that the combination of *KIT* CDS duplication and splice mutation did not completely block the melanoblast migration (Figure 5H), and a few melanocytes or their precursors could be detected in the skin hair follicles of *KIT ^Dup/D17^* mice through immunostaining for protein markers of melanocytes (Figure 5C). This was confirmed by q-PCR and Western blot analysis of additional melanocyte protein markers in the skin of *KIT ^Dup/D17^* mice (Figure 5D). In addition, in our unpublished experiments, expression of several melanocyte marker proteins were detected by q-PCR and Western blot in the skin tissue of Yorkshire pigs, implying the existence of melanocytes or their precursors in dominant white pigs. These results indicate that although the combination of the KIT CDS duplication and splice mutation impair the development of melanocytes severely, a few melanocyte precursors still migrate to their destinations.

In conclusion, our study provides further insight into the underlying genetic mechanisms of the porcine dominant white coat color.

1. **Acknowledgement**

This work was jointly supported by the National Transgenic Major Program of China (2016ZX08006003-006), National Key R&D Programs of China (2018YFD0501200) and the Key R&D Programs of Guangdong Province (2018B020203003). This manuscript has been released as a Pre-Print at https://doi.org/10.1101/688697 (Sun et al., 2019).

1. **Author Contributions Statement**

ZH and YC conceived and supervised the project. GS conducted most of the experiments. XYL is responsible for all experiments in pigs, YQ and XS participated in critical molecular experiments, PC, DM, and XHL provided suggestions on experimental design. GS and ZH wrote the manuscript. YC provided critical revisions of the manuscript. All authors read and approved the final version of the manuscript.

1. **Conflict of Interest Statement**

The authors declare that they have no competing interests.

1. Contribution to the Field Statement

KIT plays a critical role in control of coat color in mammals. Two simultaneous KIT mutations are thought to be the cause of the dominant white phenotype in pigs. One mutation is a 450-kb large duplication encompassing the entire *KIT* gene; the other is a splice mutation causing exon 17 to be skipped. The effect of these two KIT mutations on coat color formation has not yet been validated. In this study, by using genome edited mouse models, we found that neither structural mutation individually results in the dominant white phenotype, but the combination of both results in a nearly complete white coat similar to the pig dominant white phenotype. This may be due to inhibition of the kinase activity of the KIT protein, thus its signaling of the PI3K and MAPK pathways, leading to impaired melanoblast migration of during embryonic development, and eventually the dominant white phenotype. Our study provides further insight into the underlying genetic mechanisms of the porcine dominant white coat color.

1. **References**

Andersson, L. (Year). "Studying phenotypic evolution in domestic animals: a walk in the footsteps of Charles Darwin", in: *Cold Spring Harbor symposia on quantitative biology*: Cold Spring Harbor Laboratory Press), sqb. 2009.2074. 2039.

Bernex, F., De Sepulveda, P., Kress, C., Elbaz, C., Delouis, C., and Panthier, J.-J. (1996). Spatial and temporal patterns of c-kit-expressing cells in WlacZ/+ and WlacZ/WlacZ mouse embryos. *Development* 122(10)**,** 3023-3033.

Besmer, P., Manova, K., Duttlinger, R., Huang, E.J., Packer, A., Gyssler, C., et al. (1993). The kit-ligand (steel factor) and its receptor c-kit/W: pleiotropic roles in gametogenesis and melanogenesis. *Dev Suppl***,** 125-137.

Botchkareva, N.V., Botchkarev, V.A., and Gilchrest, B.A. (2003). Fate of melanocytes during development of the hair follicle pigmentary unit. *J Investig Dermatol Symp Proc* 8(1)**,** 76-79. doi: 10.1046/j.1523-1747.2003.12176.x.

Deshpande, S., Bosbach, B., Yozgat, Y., Park, C.Y., Moore, M.A., and Besmer, P. (2013). KIT receptor gain-of-function in hematopoiesis enhances stem cell self-renewal and promotes progenitor cell expansion. *Stem Cells* 31(8)**,** 1683-1695. doi: 10.1002/stem.1419.

Giuffra, E., Törnsten, A., Marklund, S., Bongcam-Rudloff, E., Chardon, P., Kijas, J.M., et al. (2002). A large duplication associated with dominant white color in pigs originated by homologous recombination between LINE elements flanking KIT. *Mammalian Genome* 13(10)**,** 569-577.

Hou, C., Miao, Y., Wang, X., Chen, C., Lin, B., and Hu, Z. (2016). Expression of matrix metalloproteinases and tissue inhibitor of matrix metalloproteinases in the hair cycle. *Experimental and therapeutic medicine* 12(1)**,** 231-237.

Imokawa, G. (2004). Autocrine and paracrine regulation of melanocytes in human skin and in pigmentary disorders. *Pigment cell research* 17(2)**,** 96-110.

Johansson, A., Pielberg, G., Andersson, L., and Edfors-Lilja, I. (2005). Polymorphism at the porcine Dominant white/KIT locus influence coat colour and peripheral blood cell measures. *Anim Genet* 36(4)**,** 288-296. doi: 10.1111/j.1365-2052.2005.01320.x.

Kent, D., Copley, M., Benz, C., Dykstra, B., Bowie, M., and Eaves, C. (2008). Regulation of Hematopoietic Stem Cells by the Steel Factor/KIT Signaling Pathway. *Clinical Cancer Research* 14(7)**,** 1926-1930. doi: 10.1158/1078-0432.ccr-07-5134.

Lennartsson, J., and Rönnstrand, L. (2012). Stem cell factor receptor/c-Kit: from basic science to clinical implications. *Physiological reviews* 92(4)**,** 1619-1649.

Lin, J.Y., and Fisher, D.E. (2007). Melanocyte biology and skin pigmentation. *Nature* 445(7130)**,** 843.

Marklund, S., Kijas, J., Rodriguez-Martinez, H., Rönnstrand, L., Funa, K., Moller, M., et al. (1998). Molecular basis for the dominant white phenotype in the domestic pig. *Genome research* 8(8)**,** 826-833.

Meurer, S.K., Ness, M., Weiskirchen, S., Kim, P., Tag, C.G., Kauffmann, M., et al. (2016). Isolation of Mature (Peritoneum-Derived) Mast Cells and Immature (Bone Marrow-Derived) Mast Cell Precursors from Mice. *PLoS One* 11(6)**,** e0158104. doi: 10.1371/journal.pone.0158104.

Moller, M.J., Chaudhary, R., Hellmen, E., Höyheim, B., Chowdhary, B., and Andersson, L. (1996). Pigs with the dominant white coat color phenotype carry a duplication of the KIT gene encoding the mast/stem cell growth factor receptor. *Mammalian Genome* 7(11)**,** 822-830.

Moniruzzaman, M., Sakamaki, K., Akazawa, Y., and Miyano, T. (2007). Oocyte growth and follicular development in KIT-deficient Fas-knockout mice. *Reproduction* 133(1)**,** 117-125. doi: 10.1530/REP-06-0161.

Pielberg, G., Olsson, C., Syvänen, A.-C., and Andersson, L. (2002). Unexpectedly high allelic diversity at the KIT locus causing dominant white color in the domestic pig. *Genetics* 160(1)**,** 305-311.

Roskoski, R. (2005). Structure and regulation of Kit protein-tyrosine kinase—the stem cell factor receptor. *Biochemical and biophysical research communications* 338(3)**,** 1307-1315.

Ruan, H.B., Zhang, N., and Gao, X. (2005). Identification of a novel point mutation of mouse proto-oncogene c-kit through N-ethyl-N-nitrosourea mutagenesis. *Genetics* 169(2)**,** 819-831. doi: 10.1534/genetics.104.027177.

Rubin, B.P., Antonescu, C.R., Scott-Browne, J.P., Comstock, M.L., Gu, Y., Tanas, M.R., et al. (2005). A knock-in mouse model of gastrointestinal stromal tumor harboring kit K641E. *Cancer research* 65(15)**,** 6631-6639.

Rubin, C.-J., Megens, H.-J., Barrio, A.M., Maqbool, K., Sayyab, S., Schwochow, D., et al. (2012). Strong signatures of selection in the domestic pig genome. *Proceedings of the National Academy of Sciences* 109(48)**,** 19529-19536.

Slominski, A., Paus, R., Plonka, P., Chakraborty, A., Maurer, M., Pruski, D., et al. (1994). Melanogenesis during the anagen-catagen-telogen transformation of the murine hair cycle. *Journal of investigative dermatology* 102(6)**,** 862-869.

Sun, G., Liang, X., Qin, K., Qin, Y., Shi, X., Cong, P., et al. (2019). Functional analysis of KIT gene structural mutations causing porcine dominant white phenotype by using genome edited mouse models. *BioRxiv***,** 688697.

Tao, F.F., Yang, Y.F., Wang, H., Sun, X.J., Luo, J., Zhu, X., et al. (2009). Th1-type epitopes-based cocktail PDDV attenuates hepatic fibrosis in C57BL/6 mice with chronic Schistosoma japonicum infection. *Vaccine* 27(31)**,** 4110-4117. doi: 10.1016/j.vaccine.2009.04.073.

Wehrle‐Haller, B., and Weston, J.A. (1997). Receptor tyrosine kinase‐dependent neural crest migration in response to differentially localized growth factors. *BioEssays* 19(4)**,** 337-345.

Wiseman, J. (1986). *A history of the British pig.* Duckworth.

**FIGURE 1. Generation of three mouse models mimicking the structural *KIT* mutations in dominant white pigs.** (A) A schematic summary of the *KIT* mutations causing the dominant white phenotype in pigs. Dominant white is associated with duplication of two to three copies of *KIT* and at least one of the KIT copies carries a splice mutation (G>A at the first base in intron 17), causing exon skipping and the expression of a KIT protein lacking an essential part of the tyrosine kinase domain. (B) A schematic summary of the *KIT* mutation mice model. To mimic the overexpression of KIT in dominant white pigs, the last exon of the *KIT* gene with its TGA stop codon mutated to GCC and linked with the CDS of another copy of *KIT* via a self-cleaving 2A peptide, and which was in turn linked to an enhanced green fluorescent protein (EGFP) reporter via the 2A peptide was knocked in to the KIT locus through CRISPR/Cas9 mediated homologous recombination. To mimic the splice mutation, two mouse models were established. One has the first nucleotide G of intron 17 of *KIT* substituted with A through CRISPR/Cas9 mediated homologous recombination, while in the other exon 17 was deleted using paired sgRNAs targeting intron 16 and intron 17. (C) Coat color of the wild-type (*KIT^+/+^*), heterologous of KIT Duplication (*KIT ^Dup/+^*) and splice mutation (*KIT ^D17/+^*) mouse models. (D) Western blotting analysis confirmed the presence of EGFP in the skin of *KIT ^Dup/+^* mice, implying that the inserted *KIT* CDS was correctly expressed. (E) RT-PCR analysis of the deletion of exon 17 of *KIT* in *KIT ^D17/+^* mice. The truncated PCR product is indicated by an arrow head. (F) RT-PCR analysis of the transcription product of *KIT* in *KIT ^GtoA/+^* mice. The PCR product with the insertion is indicated by an arrow head. (G) Sanger sequencing of cDNA from F indicates that exon 17 of *KIT* in *KIT ^GtoA/+^* mice is not removed, and that a small percentage of the transcripts contained part of intron 17. Sanger sequencing of cDNA from E indicates exon 17 was removed from the mature transcript in *KIT ^D17/+^* mice.

**FIGURE 2. Histological analysis of the back skin of 5-week old *KIT ^+/+^, KIT ^D17/+^* and *KIT ^Dup/+^* mice.** Melanin is stained with Fontana-Masson, and indicated by an arrow head. Scale bar = 50 μM.

**FIGURE 3. The KIT splice mutation reduces the number of melanocytes.** (A) Expression of KIT protein in the hair follicles of wild-type and mutant mice was determined by immunohistochemical staining. Melanin is stained with Fontana-Masson. Scale bar = 50 μM. (B) The levels of *KIT* transcription in the mast cells and skin tissue of wild-type and mutant mice were determined by qPCR analysis. (C) Expression levels of KIT, DCT, MelanA, AKT, and ERK1/2 and the phosphorylation levels of KIT, AKT, and ERK1/2 in the skin tissue of wild-type and mutant mice were determined by Western blot analysis. Statistical analysis of the relative protein expression levels is based on the intensity of the bands. * indicates p < 0.05.

**FIGURE 4. The *KIT* splice mutation affects embryonic melanoblast migration and melanin accumulation.** (A) KIT is used as marker to detect melanoblast migration in *KIT ^+/+^* and *KIT ^D17/+^* mice embryos (E14.5). Migrating melanoblasts are indicated by arrow heads. Scale bar = 500 μM. (B) Observation of hair from 2-W and 14-W old wild-type and mutant mice under a stereo microscope (left panel). Scale bar = 200 μM. The relative blackness of the hair was quantified based on the intensity of the image (right panel). * indicates p < 0.05. (C) AKT and ERK 1/2 expression and phosphorylation levels in the hair follicles of wild-type and mutant mice were determined by immunochemical staining. Scale bar = 50 μM.

**FIGURE 5. The combination of *KIT* CDS duplication and splice mutation severely impaired KIT signaling function and embryonic melanoblast migration.** (A) Identification of *KIT ^Dup/D17^* mice through PCR analysis. (B) Coat color of *KIT^+/+^* and *KIT ^Dup/D17^* mice. (C) Histological analysis of melanin accumulation in the hair follicles of *KIT^+/+^* and *KIT ^Dup/D17^* mice by Masson Fontana staining, and the presence of melanocytes in hair follicles by immunostaining for KIT. Scale bar = 50 μM. (D) KIT mRNA levels in the mast cells and skin of *KIT^+/+^* and *KIT ^Dup/D17^* mice were determined by qPCR analysis. (E) Expression levels of KIT, DCT, MelanA, S100, AKT, and ERK1/2 and the phosphorylation levels of KIT, AKT, and ERK1/2 in the skin of *KIT^+/+^* and *KIT ^Dup/D17^* mice were determined by Western blot analysis (left panel) and the base intensity of the bands was quantified (right panel). * indicates p < 0.05. (F) Expression and phosphorylation levels of AKT and ERK 1/2 in the hair follicles of *KIT^+/+^* and *KIT ^Dup/D17^* mice were determined by immunohistochemical analysis. Scale bar = 50 μM. (G) Observation of hair from 2-W and 14-W old of *KIT^+/+^* and *KIT ^Dup/D17^* mice under a stereo microscope (upper panel). Scale bar = 200 μM. The relative blackness of the hair was quantified based on the intensity of the image (lower panel). * indicates p < 0.05. (H) KIT was used as marker to detect melanoblast migration in *KIT ^+/+^* and *KIT ^Dup/D17^* mice embryos (E14.5). Migrating melanoblasts are indicated by arrow heads. Scale bar = 500 μM.

**FIGURE 6. Schematic summary of the mechanism by which KIT structural mutations affect coat color.** The SCF ligand may induce the formation of KIT/KIT ^D17^ dimers in the melanoblasts of mice with the KIT splice mutation (*KIT ^D17/+^* mice), and reduce the kinase function of KIT and impair its signaling transduction to the PI3K and MAPK pathways, which are involved in melanoblast migration, blocking the migration of some melanoblasts from the dorsal to ventral region during embryo development, resulting in a piebald mouse coat. Improved expression of the normal form of the KIT protein in mice combined with the KIT splice mutation and duplication mutation (*KIT ^Dup/D17^* mice) may increase the chance of forming a KIT/KIT ^D17^ dimer upon binding SCF from that seen in *KIT ^D17/+^* mice. Given that the amount of SCF ligand is limited, more KIT/KIT ^D17^ dimers presented on the melanoblast cell surface may significantly reduce its signaling functions, resulting in more severely impaired melanoblast migration, with most melanoblasts remaining in the neural tube, and resulting in a completely white coat. DM: dermamyotome; NT: neural tube.
